# Multi-Platform Next-Generation Sequencing of the Domestic Turkey (*Meleagris gallopavo*): Genome Assembly and Analysis

**DOI:** 10.1371/journal.pbio.1000475

**Published:** 2010-09-07

**Authors:** Rami A. Dalloul, Julie A. Long, Aleksey V. Zimin, Luqman Aslam, Kathryn Beal, Le Ann Blomberg, Pascal Bouffard, David W. Burt, Oswald Crasta, Richard P. M. A. Crooijmans, Kristal Cooper, Roger A. Coulombe, Supriyo De, Mary E. Delany, Jerry B. Dodgson, Jennifer J. Dong, Clive Evans, Karin M. Frederickson, Paul Flicek, Liliana Florea, Otto Folkerts, Martien A. M. Groenen, Tim T. Harkins, Javier Herrero, Steve Hoffmann, Hendrik-Jan Megens, Andrew Jiang, Pieter de Jong, Pete Kaiser, Heebal Kim, Kyu-Won Kim, Sungwon Kim, David Langenberger, Mi-Kyung Lee, Taeheon Lee, Shrinivasrao Mane, Guillaume Marcais, Manja Marz, Audrey P. McElroy, Thero Modise, Mikhail Nefedov, Cédric Notredame, Ian R. Paton, William S. Payne, Geo Pertea, Dennis Prickett, Daniela Puiu, Dan Qioa, Emanuele Raineri, Magali Ruffier, Steven L. Salzberg, Michael C. Schatz, Chantel Scheuring, Carl J. Schmidt, Steven Schroeder, Stephen M. J. Searle, Edward J. Smith, Jacqueline Smith, Tad S. Sonstegard, Peter F. Stadler, Hakim Tafer, Zhijian (Jake) Tu, Curtis P. Van Tassell, Albert J. Vilella, Kelly P. Williams, James A. Yorke, Liqing Zhang, Hong-Bin Zhang, Xiaojun Zhang, Yang Zhang, Kent M. Reed

**Affiliations:** 1 Avian Immunobiology Laboratory, Department of Animal and Poultry Sciences, Virginia Tech, Blacksburg, Virginia, United States of America; 2 Animal Biosciences and Biotechnology Laboratory, USDA Agricultural Research Service, Beltsville, Maryland, United States of America; 3 Institute for Physical Science and Technology, University of Maryland, College Park, Maryland, United States of America; 4 Animal Breeding and Genomics Centre, Wageningen University, Wageningen, the Netherlands; 5 European Bioinformatics Institute, Wellcome Trust Genome Campus, Hinxton, Cambridge, United Kingdom; 6 Roche Applied Science, Indianapolis, Indiana, United States of America; 7 The Roslin Institute and Royal (Dick) School of Veterinary Studies, University of Edinburgh, Roslin, Midlothian, United Kingdom; 8 Virginia Bioinformatics Institute, Virginia Tech, Blacksburg, Virginia, United States of America; 9 Chromatin Inc., Champaign, Illinois, United States of America; 10 Department of Veterinary Sciences, Utah State University, Logan, Utah, United States of America; 11 Gene Expression and Genomics Unit, National Institute on Aging, National Institutes of Health, Baltimore, Maryland, United States of America; 12 Department of Animal Science, University of California, Davis, California, United States of America; 13 Department of Microbiology and Molecular Genetics, Michigan State University, East Lansing, Michigan, United States of America; 14 Department of Soil and Crop Sciences, Texas A&M University, College Station, Texas, United States of America; 15 Center for Bioinformatics and Computational Biology, University of Maryland, College Park, Maryland, United States of America; 16 Department of Computer Science and Interdisciplinary Center for Bioinformatics, University of Leipzig, Leipzig, Germany; 17 LIFE Project, University of Leipzig, Leipzig, Germany; 18 Children's Hospital and Research Center at Oakland, Oakland, California, United States of America; 19 Institute for Animal Health, Compton, Berkshire, United Kingdom; 20 Laboratory of Bioinformatics and Population Genetics, Department of Agricultural Biotechnology, Seoul National University, Seoul, Korea; 21 Philipps-Universität Marburg, Pharmazeutische Chemie, Marburg, Germany; 22 Comparative Bioinformatics, Centre for Genomic Regulation (CRG), Universitat Pompeus Fabre, Barcelona, Spain; 23 Department of Computer Science, Virginia Tech, Blacksburg, Virginia, United States of America; 24 Wellcome Trust Sanger Institute, Wellcome Trust Genome Campus, Hinxton, Cambridge, United Kingdom; 25 Center for Bioinformatics and Computational Biology, Department of Computer Science, University of Maryland, College Park, Maryland, United States of America; 26 Department of Animal and Food Sciences, University of Delaware, Newark, Delaware, United States of America; 27 Bovine Functional Genomics Laboratory, USDA Agricultural Research Service, Beltsville Agricultural Research Center, Beltsville, Maryland, United States of America; 28 Max Planck Institute for Mathematics in the Sciences, Leipzig, Germany; 29 Fraunhofer Institut für Zelltherapie und Immunologie, Leipzig, Germany; 30 Department of Theoretical Chemistry University of Vienna, Vienna, Austria; 31 Santa Fe Institute, Santa Fe, New Mexico, United States of America; 32 Department of Biochemistry, Virginia Tech, Blacksburg, Virginia, United States of America; 33 Animal Improvement Programs Laboratory, USDA Agricultural Research Service, Beltsville Agricultural Research Center, Beltsville, Maryland, United States of America; 34 Department of Veterinary and Biomedical Sciences, College of Veterinary Medicine, University of Minnesota, St. Paul, Minnesota, United States of America; New England Biolabs, United States of America

## Abstract

A synergistic combination of two next-generation sequencing platforms with a detailed comparative BAC physical contig map provided a cost-effective assembly of the genome sequence of the domestic turkey (*Meleagris gallopavo*). Heterozygosity of the sequenced source genome allowed discovery of more than 600,000 high quality single nucleotide variants. Despite this heterozygosity, the current genome assembly (∼1.1 Gb) includes 917 Mb of sequence assigned to specific turkey chromosomes. Annotation identified nearly 16,000 genes, with 15,093 recognized as protein coding and 611 as non-coding RNA genes. Comparative analysis of the turkey, chicken, and zebra finch genomes, and comparing avian to mammalian species, supports the characteristic stability of avian genomes and identifies genes unique to the avian lineage. Clear differences are seen in number and variety of genes of the avian immune system where expansions and novel genes are less frequent than examples of gene loss. The turkey genome sequence provides resources to further understand the evolution of vertebrate genomes and genetic variation underlying economically important quantitative traits in poultry. This integrated approach may be a model for providing both gene and chromosome level assemblies of other species with agricultural, ecological, and evolutionary interest.

## Abstract 2

The combined application of next-generation sequencing platforms has provided an economical approach to unlocking the potential of the turkey genome.

## Author Summary

In contrast to the compact sequence of viruses and bacteria, determining the complete genome sequence of complex vertebrate genomes can be a daunting task. With the advent of “next-generation” sequencing platforms, it is now possible to rapidly sequence and assemble a vertebrate genome, especially for species for which genomic resources—genetic maps and markers—are currently available. We used a combination of two next-generation sequencing platforms, Roche 454 and Illumina GAII, and unique assembly tools to sequence the genome of the agriculturally important turkey, *Meleagris gallopavo*. Our draft assembly comprises approximately 1.1 gigabases of which 917 megabytes are assigned to specific chromosomes. Comparisons of the turkey genome sequence with those of the chicken, *Gallus gallus*, and the zebra finch, *Taeniopygia guttata*, provide insights into the evolution of the avian lineage. This genome sequence will facilitate discovery of agriculturally important genetic variants.

## Introduction

The rapid and continuing development of next-generation sequencing (NGS) technologies has made it feasible to contemplate sequencing the genomes of hundreds—if not thousands—of species of agronomic, evolutionary, and ecological importance, as well as biomedical interest [1]. Recently, a draft genome of the giant panda was described, based solely on Illumina short read sequences [2]. Below, we describe the genome sequence of the turkey (*Meleagris gallopavo*) determined using primarily NGS platforms. In this case, however, a combination of Roche 454 and Illumina GAII sequencing was employed. While this approach presented unique assembly challenges, the turkey sequence benefits from the particular advantages of both platforms. In addition, unlike the case for the panda, this novel approach allowed us to use a BAC contig-based physical and comparative map, along with the turkey genetic map [3] and the chicken genome sequence [4], to align the turkey sequence contigs and scaffolds to most of the turkey chromosomes. Such an alignment is essential for making long range evolutionary comparisons and employing the sequence to improve breeding practices using, for example, genome-based selection approaches, where chromosome locations are critical.

The high throughput and low cost of NGS technologies allowed sequencing the turkey genome at a fraction of the cost of other recently reported genomes of agricultural interest (bovine and equine) [5],[6]. The draft turkey genome sequence represents the second domestic avian genome to be sequenced, and this permits a genome-level comparison of the two most economically important poultry species. When added to the recently published zebra finch genome [7], analysis of the three avian genomes reveals new insights into the evolutionary relationships among avian species and their relationships to mammals.

Turkeys, like chickens, are members of the *Phasianidae* within the order *Galliformes*. One estimate [8] is that the last common ancestor of turkeys and chickens lived about 40 million (M) years ago; however, other estimates are more recent [9],[10]. Comparison of the turkey genome to that of the chicken provides the opportunity for high resolution analysis of genome evolution within the *Galliformes*. The turkey has 2n = 80 chromosomes (chicken has 2n = 78) and, as for most avian species, the majority of these are small “microchromosomes” that cannot be distinguished by size alone. Although most turkey chromosomes are syntenic to their chicken orthologues, the chicken chromosome GGA2 is orthologous to two turkey chromosomes, MGA3 (GGA2q) and MGA6 (GGA2p), due to fission at or near the centromere, while GGA4 is orthologous to MGA4 (GGA4q) and MGA9 (GGA4p) [10],[11].

## Results and Discussion

### Sequencing, Assembly, and Sequence Analyses

Generally, DNA from a single inbred animal is preferential for sequencing to minimize polymorphism. For the turkey, however, such an option is not available, and thus we sequenced DNA from “Nici” (Nicholas Inbred), a female turkey, which is also the source DNA for the two BAC libraries that have been characterized [12]. Nici is from a subline (sib-mating for nine generations) originally derived from a commercially significant breeding line, but her genome is still extensively heterozygous. A side benefit of this approach was the concomitant identification of extensive and commercially relevant single nucleotide polymorphism (SNP) data, as discussed below.

With the exception of the BAC end sequences (BES) used only for chromosome alignment, the sequence data used for this assembly came solely from two sequencing platforms: the Roche/454 GS-FLX Titanium platform (454 Life Sciences/Roche Diagnostics, Branford, CT) and the Illumina Genome Analyzer II (GAII; Illumina, Inc., San Diego, CA). The 454 data were generated using the latest “Titanium” protocol at Roche and the Virginia Bioinformatics Institute (Virginia Tech) and included both unpaired shotgun reads and paired-end reads produced from two libraries with estimated 3 kilobase pair (Kbp) and 20 Kbp fragment sizes. The 454 runs yielded approximately 3 M read pairs from the 3 Kbp library (average usable read length 180 bases), 1 M read pairs from the 20 Kbp library (average length 195 bases), and 13 M shotgun reads (average length 366 bases). The Illumina sequencing data were generated at the USDA Beltsville Agricultural Research Center and the NIH National Institute on Aging from both single and paired-end read libraries with a 180 bp fragment size for the paired reads. Details on the sequence data are presented in Table 1. These data represent approximate 5× genome coverage in 454 reads and 25× coverage in GAII reads, assuming a genome size similar to that of the chicken at 1.1 billion bases [4]. In addition, BACs used to generate the 40,000 BES alignment markers by traditional Sanger sequencing spanned ∼6× clone coverage of the genome. Since female DNA was used, coverage of the Z and W sex chromosomes was half that of autosomes; therefore the assembly of both these chromosomes was poor.

**Table 1:** Summary of the Roche 454 and Illumina GAII data used for assembling the turkey genome sequence.

	Number of Reads (Million)	Average Usable Read Length (bp)
454/Roche data:		
Shotgun	13	366
3 Kbp paired end	3	180
20 Kbp paired end	1	195
Illumina data:		
Shotgun	200	74
180 bp paired end	200	74

A modified version of the Celera Assembler 5.3 [13],[14] was used to produce the contigs and scaffolds in the assembly (see Methods for details). The initial assembly contained 931 Mbp of sequence in 27,007 scaffolds with N50 size of 1.5 Mbp. The span of the scaffolds was 1.038 Gbp. The scaffolds contained 145,663 contigs with N50 size of 12.6 Kbp. The assembled scaffolds were then ordered and oriented on turkey chromosomes using a combination of two linkage maps and a comparative BAC contig physical map. The first turkey linkage map [3] had 405 chicken and turkey microsatellite sequences that mapped to the assembled scaffolds. The second linkage map, based on segregation of SNPs in a different population [15], had 442 SNP markers mapped to the scaffolds. The comparative chicken-turkey physical map [16] provided turkey chromosome positions for 30,922 BES found in scaffolds.

Comparison of scaffolds to the marker map resulted in splitting only 39 scaffolds due to inconsistencies between the assembled scaffolds and marker positions on the chromosomes. A total of 28,261 scaffolds containing 917 Mb of sequence were assigned to chromosomes (Table 2). Included in this number were 7,080 single-contig scaffolds that represented repetitive sequences but that could be linked to non-repetitive scaffolds. The remaining 5,858 scaffolds were pooled to form ChrUn (unassigned) which contains 19 Mb of sequence in comparison to about 64 Mb on the current chicken chr_Un.

**Table 2:** Chromosome sizes in the draft turkey genome assembly.

Chromosome	Number of Contigs	Number of Bases (Excluding Gaps)
1	26,557	181,826,552
2	14,384	106,718,223
3	12,649	91,132,767
4	9,170	68,844,569
5	7,553	56,965,239
6	6,534	48,705,183
7	4,755	35,338,084
8	4,751	35,279,744
9	2,286	18,014,631
10	3,733	28,668,829
11	2,720	22,659,912
12	2,372	18,944,919
13	2,354	18,696,996
14	2,367	19,181,786
15	2,265	16,791,072
16	1,967	14,411,805
17	1,635	12,015,459
18	51	139,801
19	1,399	9,478,246
20	1,424	9,943,105
21	1,328	9,405,728
22	1,865	13,252,797
23	937	6,420,024
24	569	3,613,335
25	834	4,963,017
26	1,040	5,925,429
27	161	687,724
28	717	4,244,239
29	803	3,649,262
30	693	3,524,564
W	50	108,225
Z	24,970	47,735,835
Un	7,748	18,627,908
Total	152,641	935,915,009

Analysis of the assembled contigs showed that 4.6% of the sequence was covered only by reads from a single sequencing platform, with 2.3% covered exclusively by each. If the reads covered the genome uniformly, one would expect to have missed only 0.67% of the genome with Roche/454 and 0.0006% with Illumina. The distribution of regions of exclusive coverage for both platforms (Figure S1) shows there was a large number of short (<20 bp) gaps in coverage by Illumina sequencing, whereas the Roche/454 coverage gaps tended to be larger. Mean sequencing gaps were 46 bases for Illumina reads and 72 for the Roche/454 coverage. Coverage biases previously have been shown for both platforms [17], but fortunately, the biases are relatively orthogonal. Therefore, it is definitely beneficial to use data from both platforms in de novo assemblies.

The draft turkey assembly was compared to the chicken genome assembly (2.1), which was sequenced and assembled using traditional Sanger sequencing [4]. Table 3 illustrates that assembly of NGS sequence data, although feasible, does not produce contigs and scaffolds as large as those expected from an assembly based on Sanger sequencing. However, the relatively low cost of NGS sequencing (<$250,000 for the turkey) makes such projects feasible for species with more focused interest groups and facilitates for resources to be directed toward genome analysis and interpretation as opposed to generating raw sequence data. However, chromosome assemblies currently still require the integration of multiple data types including shotgun reads and contigs, genetic linkage maps, BAC maps and BES, and cytogenetic assignments. The challenge was to develop databases and software to achieve this goal.

**Table 3:** Major characteristics of the turkey and chicken genome assemblies.

	Turkey 2.01	Chicken 2.1
Number of scaffolds >1 Kb	26,917	32,767
Number of contigs >1 Kb	128,271	98,612
Scaffolded sequence (excluding gaps)	931 Mb	1,047 Mb
Largest scaffold	9 Mb	33 Mb
N50 scaffold size	1.5 Mb	7.1 Mb
N50 contig size	12,594 b	36,000 b
Largest contig	90 Kb	442 Kb
Contig coverage	17×	7×
Cost of sequencing	<$0.25 M	>$10 M

Integrity of the assembly was validated by mapping the assembled turkey scaffolds to 197 Kbp of finished BAC sequence containing part of the MHC B-locus, GenBank accession DQ993255.2. The average sequence similarity was over 99.5% and no inconsistencies in the 21 scaffolds that mapped to that region were observed. The extent of the genome coverage could be estimated both from the total span of the assembled scaffolds and from portions of the chicken genome with syntenic matches to the turkey scaffolds. Both methods produced consistent estimates of the size of the euchromatic portion of the turkey genome at about 1.05 Gbp. With 936 Mbp of sequence in the final chromosomes, including ChrUn, the assembly encompasses an estimated 89% of the total sequence of the genome.

One of the striking observations in the chicken genome sequencing project was the difficulty obtaining sequences for specific regions, including the 10 smallest microchromosomes [4]. For example, the chicken genome lacks sequence orthologous to human chromosome 19q. Remarkably, these sequences appeared to be absent not only from the shotgun clone libraries used to generate the whole genome shotgun (WGS) reads but also from all available BAC libraries [18]. Although these regions have high GC content, it is unclear why this region of the genome is resistant to cloning in *E. coli*. In general, BAC coverage of microchromosomes is less than macrochromosomes in both chicken and turkey BAC libraries, although the HSA19q orthologues are an extreme example of a missing syntenic region. Since the turkey genome was sequenced without any cloning step, the assembly was tested for representation of HSA19q orthologous sequence. Presence of sequences was verified by performing a BLAT analysis of the complete HSA19q sequence against the turkey and chicken genomes (Table S1). Surprisingly, regions orthologous to HSA19q were not represented at a higher frequency in the turkey assembly versus the chicken assembly. As was observed in the chicken, regions orthologous to HSA19p and a small syntenic region from HSA19q are covered well in the turkey assembly (MGA30 and 13, respectively). These results suggest that absence of HSA19q orthologous sequences is not due to the high GC content, in that Illumina sequences show a bias towards higher coverage of GC rich regions [19],[20]. The identification of a single BAC clone that hybridizes across the entire length of a single microchromosome in chicken [21] suggests that the occurrence of microchromosome-specific repeats might be a more likely explanation for the absence of these sequences using both traditional Sanger sequencing as well as NGS technologies.

### Single Nucleotide Variants (SNVs)

Heterozygous alleles, including both SNPs and single nucleotide insertions and deletions (indels), were detected by scanning the assembled contigs for positions where the underlying reads significantly disagreed with the consensus base [22]. A previous study cataloging heterozygous alleles from assembled shotgun reads within an individual human genome used a similar approach, augmented with a set of quality criteria used to distinguish genuine biological variations from sequencing error [23]. Following this approach, a set of quality criteria was developed and implemented within the assembly forensics toolkit [24]. Two classes of SNVs were catalogued: (1) those with abundant evidence, called strong SNVs (601,490 SNVs), and (2) a more inclusive set called weak SNVs (920,126 SNVs total).

In the turkey genome, transitions were roughly 2.4× more common than transversions: 295,055:122,731 for strong SNVs and 466,629:200,743 for all SNVs. Many single base indel positions were detected: 183,215 of 601,490 strong SNVs, and 249,512 out of all 920,126 SNVs. A very small number of SNVs (489 strong, and 3,242 all) were detected with more than two well-supported variants, suggestive of unfiltered sequencing errors or collapsed repeats. The depth of coverage for strong SNVs ranged between 6 and 30 with mean and standard deviation of 15.3±5.3, while the depth of coverage for all SNVs ranged between 4 and 5,319 with mean and standard deviation of 41.4±134.6. The very high coverage regions are highly likely to be due to collapsed near-identical repeats.

### Annotations of Protein-Coding Genes

Annotation of the turkey genome sequence identified a total of 15,704 genes (Table S2) of which 15,093 were distinct protein coding loci and 611 non-coding RNA genes. In addition, multiple distinct proteins produced by alternative splicing were identified for some loci, giving a total of 16,217 distinct protein sequences. Orthologs between turkey, chicken, and human proteins were defined using sequence homology, phylogenetic trees, and conservation of synteny. All gene annotations are available from the Ensembl genome browser version 57 (http://e57.ensembl.org).

### Nucleotide Diversity across the Turkey Genome

The draft turkey genome assembly was used to test the distribution of nucleotide diversity across the turkey genome by aligning SNPs covering ∼3.97% of the genome identified through resequencing a reduced representation library from commercial turkeys [15]. Substantial deviations were observed between regions in the genome. Chromosome Z showed the lowest nucleotide diversity, about half (θ = 0.000273) that of the autosomes, which is likely the result of a lower effective population size of this chromosome and lower recombination rate (Figure S2) [25]. The five largest chromosomes had similar nucleotide diversities as the microchromosomes. Given the higher recombination rate on the microchromosomes, the ensuing higher mutation rate [26], and lower susceptibility to hitchhiking effects, equal rates of nucleotide diversity between micro- and macrochromosomes may seem unexpected. However, these findings are in line with observations in the chicken [27] and may be explained by higher gene density and higher purifying selection on the microchromosomes. Within chromosomes, extended regions of low nucleotide diversity were detected, many of which coincided with centromeres.

### Comparative Genome Analyses

#### Chromosomal evolution within galliformes

As noted above, low resolution cytogenetic analyses [10],[11] demonstrate that a limited number of chromosomal rearrangements differentiate the turkey and chicken genomes. With the turkey genome assembly, a more detailed comparison is now possible. A list of predicted rearrangements (∼30) between the turkey and chicken genomes identified through alignment of the BAC contig physical map with the chicken genome sequence is provided in Table S3. Since alignment of turkey sequence scaffolds to chromosomes depends on this map (except for MGAZ and W), these rearrangements are also reflected in the sequence assembly. Generally, each predicted rearrangement was detected by multiple BES mate pairs across a given breakpoint, and most have been confirmed by overgo hybridization analysis and/or high resolution fluorescence in situ hybridization (FISH) studies [16]. It is possible that some predicted rearrangements are due to inaccuracies in the chicken sequence assembly, especially on the smallest microchromosomes (e.g., GGA28 [18]). In all cases tested to date, comparative FISH analyses on chicken and turkey chromosomes have confirmed that both the chicken genome sequence and the predicted rearrangement in the turkey genome are correct (e.g., Figure S3).

**Figure 1:**
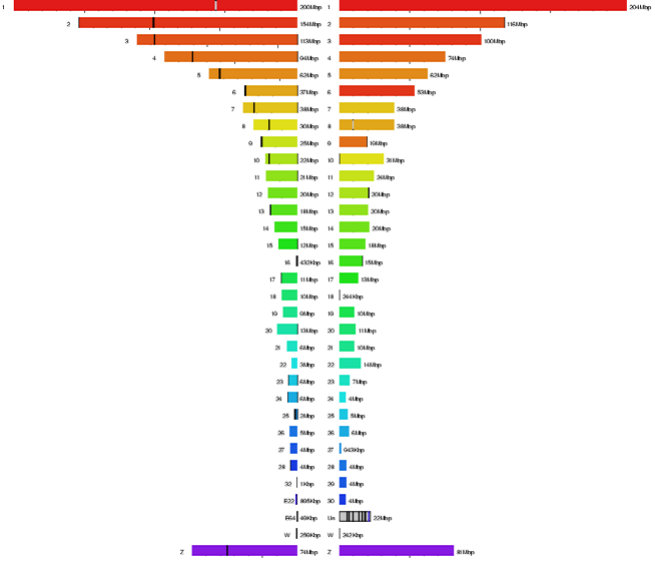
Synteny map of chicken (left) and turkey (right). Each chromosome is assigned a color in the chicken chromosome, ranging from red (Chr 1) through the spectrum to yellow, green, and blue. Turkey chromosomes are shown using the same colors, indicating differences due to chromosome numbering; e.g., turkey Chr 8 matches chicken Chr 6. The figure shows that there have been no large-scale chromosomal rearrangements in either species since their divergence.

The predicted rearrangements between the turkey and chicken genomes exhibit several trends. Based on comparative genetic maps, avian genomes have been relatively stable to rearrangements during the course of avian evolution [28]. This conclusion is consistent at least in *Galliformes* evolution, for both the turkey and chicken genomes. First, as observed in lower resolution cytogenetic studies [10], these two avian genomes are quite similar in overall genome architecture despite up to 40 M years of separate evolution (Figure 1). At the level of resolution of the BES mate pairs, only about 30 rearrangements (Table S3) are detected that distinguish the two genomes (∼0.4 chromosome rearrangements per million years). Although differences in sequencing and assembly approaches make it difficult to compare turkey and chicken to similar pairs of other species, it is notable that the rhesus macaque differs from human by 48 cytogenetically identifiable breakpoints (25 M years to last common ancestor [29]), whereas chicken and turkey differ by only five events at this level of resolution [10]. The estimate of about 30 events (at the resolution provided by the BAC map, 50–100 Kbp) can be compared to 56 events of 50 Kbp or larger between human and macaque [30], with the majority of those events also being inversions (∼1.1 chromosome rearrangement per million years; almost 3 times that found in the two avian species).

Among the identified rearrangements, inversions are most frequent and translocations are rare. Turkey chromosomes show a trend towards shorter p arms versus their chicken orthologs (Table S3). Only two apparent interchromosomal translocations of segments attributed to GGA4 which appear to be on MGA1 were detected. These, however, are small enough that they could represent repeated sequences in the ancestral galliform genome of which turkey retained one copy and chicken another, or possibly translocations due to the action of transposable elements. In one case, a segment on GGA1 is flanked on both sides by *CR1* LINE sequences. Several rearrangements are also observed that are likely due to unequal recombination between members of gene families. For example, data suggest that the inversion of GGA8p in relation to MGA10 [10] may be due to unequal recombination between two duplicate α-amylase loci [31], one adjacent to the p telomere of GGA8 and the other (inverted in orientation) adjacent to the centromere. Another such rearrangement on MGA20/GGA18 between *NME* gene paralogues is demonstrated in Figure S3. There are also suggestions of unequal recombination within the *SEMA3* gene cluster on MGA1/GGA1 being involved in the translocation of an internal segment and within a *KCN* gene cluster on the same chromosome leading to a short inversion. These are probably just a few examples of the wider trend for evolutionary breakpoints to be located at sites of copy number variation (CNV) [32], whether within gene families or other nearby repeats.

#### Three-way avian genome alignments

**Figure 2:**
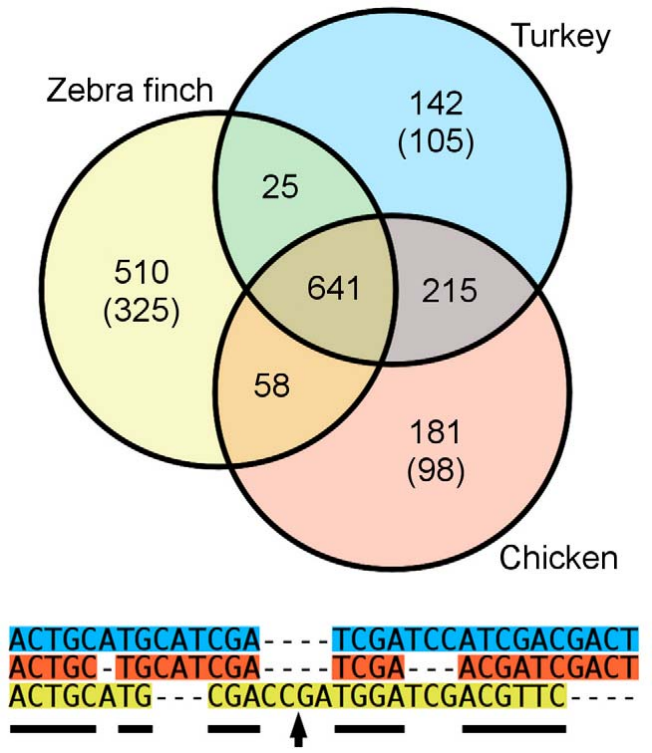
Venn diagram showing the amount of sequence (in Mbp) aligned among the three avian genomes. Numbers in brackets refer to the amount of sequence that is part of the alignments, but as species-specific insertions. For instance, out of the 142 Mbp of the turkey genome not aligned to the other two genomes, 105 Mbp are included in the alignments as turkey-specific insertions. The lower panel shows an example alignment. Regions where all three species are aligned are highlighted with a black line, and species-specific sequence is shown with an arrow.

Multiple (three-way) alignments were built on the turkey, chicken [4], and zebra finch [7] genomes using Pecan [33]. Coverage of the resulting alignments includes 92.39% of the turkey genome, 91.92% of the chicken genome, and 81.51% of the zebra finch genome, although only 641 Mbp of sequence were aligned across all three species (Figure 2). Regions under evolutionary constraint were detected with GERP [34]. While the fraction of constrained regions in placental mammals is around 5% [35],[36], 9.87% of the turkey genome is under constraint (compared to 8.58% of the chicken and 7.50% of the zebra finch genomes). High levels of sequence constraint (40.34%–60.73% of the bases are under evolutionary constraint as defined by GERP, Table 4) in groups of repeats, namely in Eulor, MER, UCONS, X*-LINE, and SINE, are similar for the turkey to those noted for transposable elements in the opossum genome [37]. In contrast, only 7.52% of the same MER repeats in the human genome (at the base level) are conserved in placental mammals. Thus, regardless of the larger percentage in birds, the total amount of constrained sequence is lower because their genomes are more compact than placental mammalian genomes. The span of the neutral tree used in this analysis is roughly 2/3 that of the human, mouse, and rat neutral tree. As additional avian genomes become available, a larger fraction of the turkey genome may be shown to be under constraint.

**Table 4:** Conservation of repetitive DNA.

Repeat Group	Number of Repeats	Total Length of Repeats	Total Number of Conserved Bases	As a Percentage
Eulor	1,581	214,392	130,210	60.73%
UCONS	3,281	508,818	262,553	51.60%
MER	1,686	225,328	127,573	56.62%
X*-LINE	876	125,896	63,185	50.19%
SINE	2,900	413,703	166,890	40.34%

Listed are the numbers of repeats and their conservation for the most conserved repeats.

GERP constrained elements were used to define the set of conserved bases.

### Lineage-Specific Expansion/Contraction of Protein-Coding Gene Families

**Figure 3:**
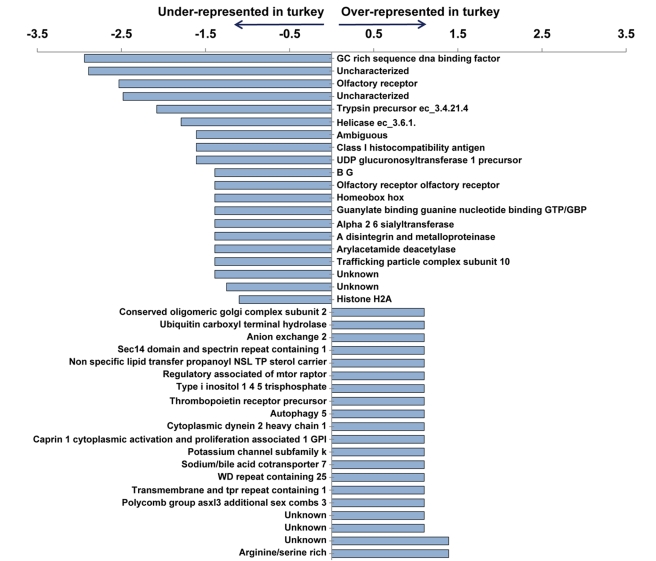
Top 20 most expanded and contracted gene families in turkey genome assembly as compared to the chicken. The axis is the log ratio of copy number in turkey versus copy number in chicken.

Comparisons of gene family assignment statistics for the turkey and chicken genome assemblies are shown in Table S4. Although the draft turkey sequence has fewer genes than the current chicken genome build (2.1), part of the difference may be due to cutoff values used by annotation groups resulting in variation in gene number. Even with this caveat, more than half of the gene families show no change in copy number between them (Table S5a–d). Overall, most families exhibiting variation have general regulatory functions related to transcription, metabolism, cation transport, cell-cell signaling, and cell development or differentiation (Figure 3). Distinct keratin families, encoding major structural proteins of chicken feathers, claws, and scales, have undergone uneven expansion or contraction with considerable variation in number among species. More than half of the innovation families (found in turkey but not chicken) have unknown functions, are singletons, and were annotated by mapping to the zebra finch protein prediction.

Species-specific gene families in birds and mammals are summarized in Tables S6, S7. Of these, 881 are specific to turkeys and chickens and 271 specific to birds. The inference for bird-specific functions is of relatively high quality since the likelihood that a bird gene is not simultaneously found in all 13 non-bird species is low. Most of the rapidly evolving gene families in birds have unknown functions. Approximately 83% of the turkey/chicken-specific families and 71% of the bird-specific families have unknown functions. For the remaining families, most have well-defined roles (Table 5). Families related to egg formation (such as avidin, ovocalyxin, and vitellogenin) and scavenger receptors were identified as avian specific in the present and previous analyses [4]. Examination of gene family sizes between the avian species and the platypus, an egg laying mammal, found two egg-related gene families [egg envelop protein (ENSFM00500000271806) and vitellogenin, an egg yolk precursor protein (ENSFM00250000000813)] to be conserved among the four egg-laying species. Both of these gene families are absent from eutherians. Other gene families specific to egg-laying species (birds and platypus) are mainly related to protein metabolism, cell-cell communication, and regulatory functions. Several other proteins related to egg formation, such as avidin and ovocalyxin, are found in birds but not in platypus.

**Table 5:** Top 20 avian-specific gene families with known functions.

Family ID	Turkey	Chicken	Zebra Finch	Non-Avian Species	Description
ENSFM00500000278106	5	5	2	0	Cytidine deaminase
ENSFM00250000010664	1	3	1	0	C type lectin
ENSFM00520000517850	1	3	10	0	Class II histocompatibility antigen b l, beta chain fragment
ENSFM00250000011687	1	2	1	0	Early response to neural induction ERNI
ENSFM00540000719139	1	1	1	0	16 kDa beta galactoside binding lectin C, 16 galectin (CG 16)
ENSFM00250000030665	1	1	1	0	2 receptor
ENSFM00500000306697	1	1	1	0	28 s ribosomal S6 mitochondrial S6mt MRP-S6
ENSFM00540000721500	1	1	1	0	Amyloid precursor
ENSFM00250000013480	1	1	1	0	B6 BU
ENSFM00500000292985	1	1	1	0	CD30 ligand
ENSFM00540000719360	1	1	2	0	CD30 precursor
ENSFM00500000279114	1	1	2	0	CD47 glycoprotein
ENSFM00540000720384	1	1	1	0	CD5 precursor
ENSFM00540000719692	1	1	1	0	CD80
ENSFM00500000291092	1	1	1	0	CD86 precursor
ENSFM00500000281340	1	1	1	0	CENP-C
ENSFM00500000296154	1	1	1	0	Centromere Q [CENP-Q]
ENSFM00540000721306	1	1	1	0	Centromere U [CENP-U]; centromere p50 of 50 kDa CENP-50 MLF1 interacting protein
ENSFM00500000287565	1	1	1	0	Cholecystokinin precursor CCK [contains cholecystokinin (CCK); CCK-8; CCK-7]
ENSFM00560000772828	1	1	1	0	COMM domain-containing protein 6

In contrast to unique gene families, only 70 families were completely absent in both the turkey and chicken (33 in all birds) compared to the non-avian species. These include the gene family associated with enamel formation (ENSFM00250000008876, an enamelin precursor related to teeth), which is completely lost in the three avian species. Genes encoding the vomeronasal receptors and several casein related families are also completely absent in the avian species. Several olfactory receptor families specific to mammals are either absent or dramatically reduced in birds. Interestingly, the olfactory receptor 5U1 and 5BF1 gene families, reported to be dramatically expanded in chicken as compared to humans and flies [4], is contracted in turkey.

### Synonymous/Non-Synonymous Mutation Rates Vary Widely Across the Avian Genome

**Figure 4:**
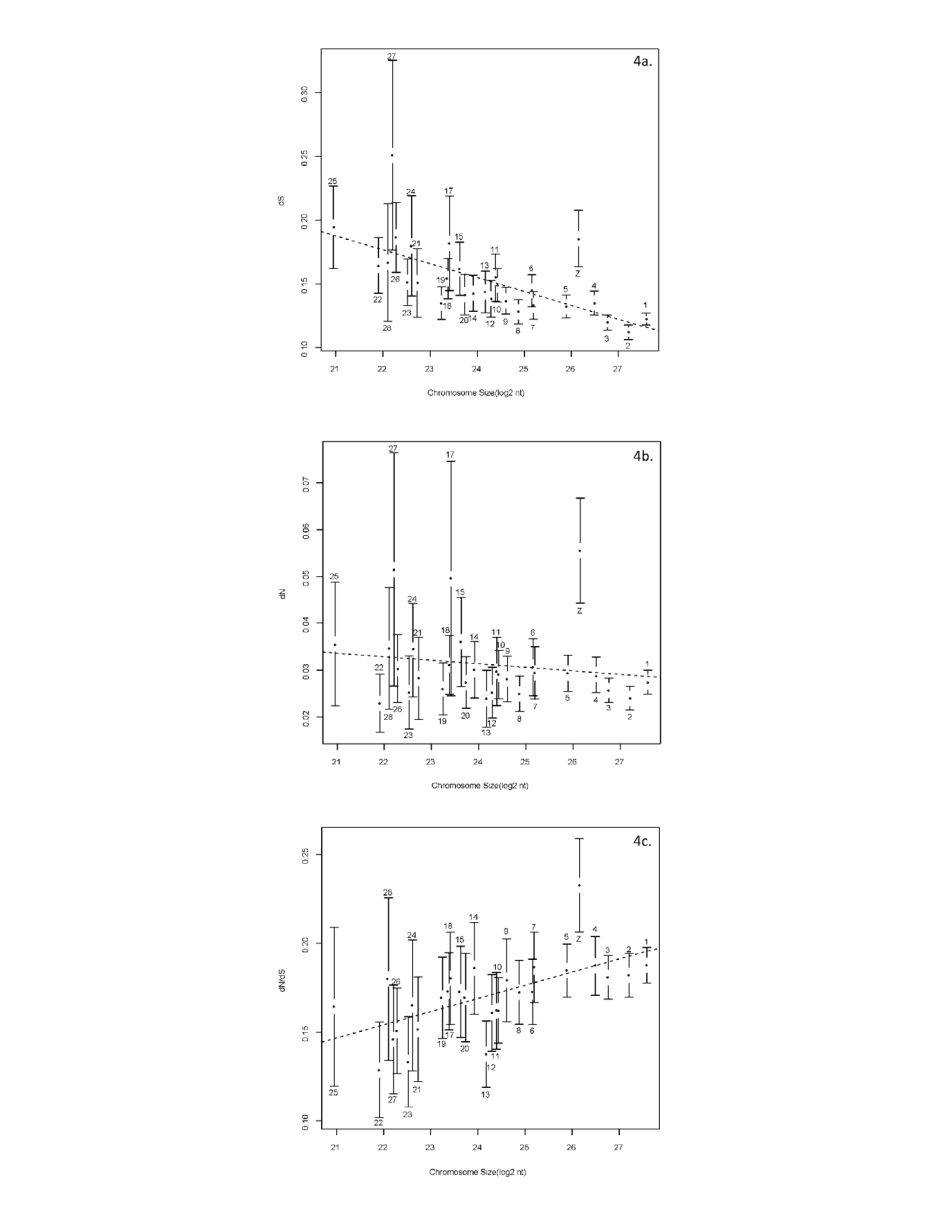
Lineage events in the turkey: variation of (a) synonymous (*d_S_*), (b) non-synonymous (*d_N_*), and (c) *d_N_*/*d_S_* ratios based on chromosome sizes. The chromosome lengths are expressed as log base2 (nucleotide lengths in base pairs).

Lineage events in the turkey, chicken, and zebra finch genomes reveal significantly higher synonymous substitution rates on microchromosomes than macrochromosomes (Figure 4a), with a clear inverse relationship with chromosome size. This suggests that genes on the microchromosomes are exposed to more germ-line mutations than those on other chromosomes [38]. However non-synonymous mutation rates do not seem to vary so widely and when combined show the *d_N_*/*d_S_* ratio (a measure of selection) to increase with chromosome size. These results are consistent with the prediction that the higher synonymous substitution rates of microchromosomes combined with the “Hill-Robertson” effect [39] of higher recombination rates on these smaller chromosomes increases purifying selection [40] on the microchromosomes (Figure 4b and 4c).

**Figure 5:**
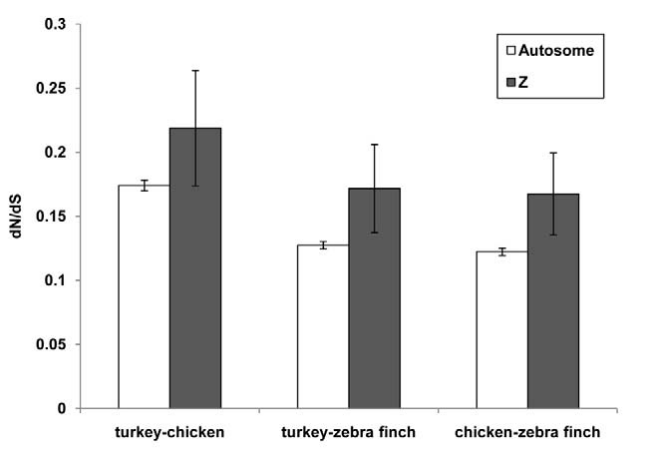
Rapid evolution of sex-linked genes in birds.

Theory predicts natural selection to be more efficient in the fixation of beneficial mutations in mammalian X-linked genes than in autosomal genes, where hemizygous exposure of beneficial non-dominant mutations increases the rate of fixation. This “fast-X effect” should be evident by an increased ratio of non-synonymous to synonymous substitutions (*d_N_*/*d_S_*) for sex-linked genes. As shown in Figure 5, there is solid confirmation of the predicted rapid evolution in the sex-linked genes based on turkey, chicken, and zebra finch genome-wide data. These results confirm that evolution proceeds more quickly on the Z chromosome [41], where hemizygous exposure of beneficial non-dominant mutations increases the rate of fixation.

### Evolution of Genes in Avian Lineages

**Figure 6:**
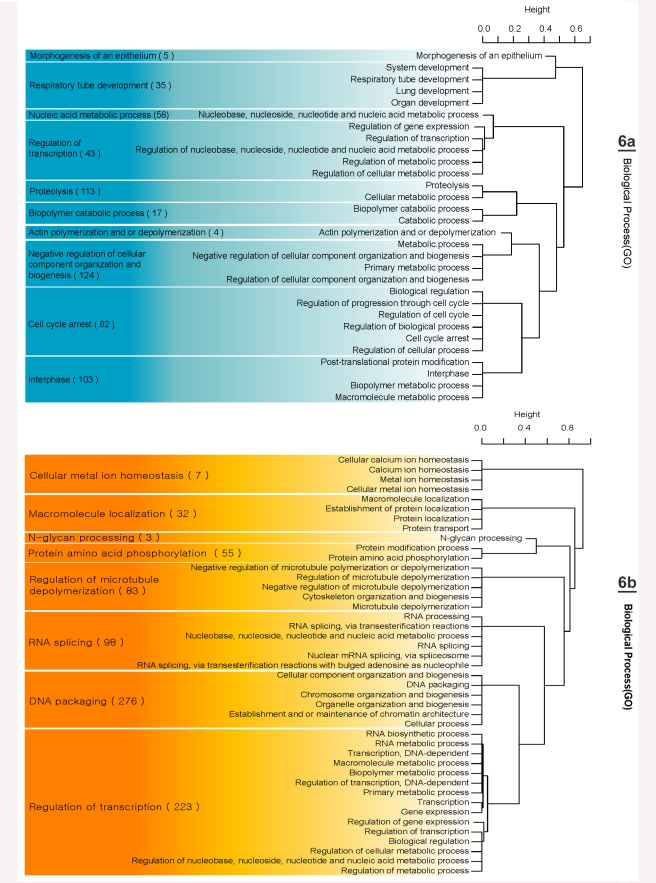
Significant GO terms in the accelerated genes in: (a) turkey compared with chicken and (b) chicken compared with turkey. Number in parenthesis indicates non-redundant number of genes in each group. The representative term in each group was selected manually.

Based on the analysis of differentially evolved genes, 428 and 257 genes were identified as being under accelerated evolution in the turkey and chicken lineages, respectively. Most of the accelerated genes in the turkey lineage have gene ontology (GO) terms related to DNA packaging and regulation of transcription (Figure 6a). In contrast, a large proportion of the accelerated genes in the chicken lineage have GO terms related to negative regulation of cellular component organization and biogenesis, proteolysis, interphase, and cell cycle arrest (Figure 6b). The enrichment of KEGG pathways using DAVID supports the GO term analysis (Table S8). These results suggest that genes with a role in transcriptional regulation are key in the evolution of the turkey, whereas genes involved in protein turnover and cell proliferation have been more important in the evolution of the chicken.

**Figure 7:**
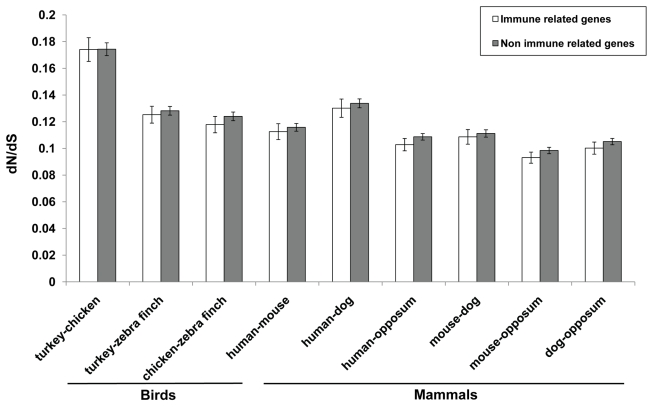
Comparison of the *d_N_/d_S_* ratios between innate immune related genes and other genes. Error bars indicate 95% standard error of the mean d*_N_*/d*_S_* ratios. Significance tests were performed using Wilcoxon rank sum test since the d*_N_*/d*_S_* ratios did not follow normal assumptions (Table S9).

For genes classified as innate immune loci by InnateDB (www.innatedb.ca), *d_N_*/*d_S_* ratios were calculated for each pair of species (turkey-chicken, turkey-zebra finch, etc.) and then compared with ratios for non-immune genes. Innate immune genes showed lower *d_N_*/*d_S_* ratios than other genes in all species-pairs of mammals and birds, except between turkey and chicken where the values are essentially equal (Figure 7). Using Wilcoxon rank sum test, it is obvious from the comparisons that the innate immune-related genes have been under more purifying selection than non-immune-related genes except between turkey and chicken (Table S9). Evolution of genes of the innate immunity system is thought to be continuous and under balancing selection [42]. However, purifying selection under the same conditions may be the dominant force acting on the vast majority of genes that function within the innate immune system [43]. Although only innate immune genes are under purifying selection by functional constraints, they are also more constrained than other genes. This relationship supports the view that the ancient innate immune system has a highly specialized function, critical for the recognition of pathogens and thus should be under purifying selection. However, unlike other species, the *d_N_*/*d_S_* ratios for innate immune genes between turkey and chicken are similar to other genes. Perhaps the adaptation of turkey and chicken to different ecological niches has exposed them to new pathogenic environments with potentially lethal pathogens having exerted selective pressures on their genomes. This thesis would suggest that there was a period of accelerated evolution of the innate immunity system after the divergence of these species 30–40 M years ago.

### Comparison of the Immune Gene Repertoire of Birds and Mammals

The availability of the turkey genome for comparison to the chicken [4] and zebra finch [7] allows for interrogation of the immune gene repertoire. In general, homologs for all the innate immune gene families were found (Table 6), with smaller gene families present in birds. This finding is consistent with earlier comparisons of mammalian with the chicken genome [44] and provides greater evidence of an avian-wide phenomenon. Examples include the chemokines, TNF superfamily, and pattern recognition receptors. Inflammatory CCL chemokines, which occur in all avian and mammalian species, fall into two multigene families (MIP and MCP; Figure S4). There are four MIP family members in the chicken and the zebra finch (CCLi1–4), yet only three family members in the turkey genome build (CCLi2–4). For the MCP family, there are six (CCLi5–10), three (CCLi5–7), and five (CCLi5–7 and 9–10) members in the chicken, zebra finch, and turkey genomes, respectively.

**Table 6:** Innate immune system genes found in turkey, chicken, zebra finch, mouse, and human genomes.

	Birds			Mammals	
Gene Family Name	Turkey	Chicken	Zebra Finch	Human	Mouse
*Chemokines*					
CCL chemokines	11	14	11	27	24
CXCL/CX3CL chemokines	7	9	9	12	13
XCL chemokines		1		2	1
Chemokine receptors	14	15	14	20	20
*Interleukins*					
IL-1	2	4	2	10	9
IL-1 receptor family	11	11	11	11	11
IL10 family	4	4	4	6	5
IL-10 receptor family	5	5	5	5	5
IL-12 receptor family	2	2	2	4	4
IL-16 family	1	1	1	1	1
IL-17 family	5	5	5	6	6
IL-32				1	
IL-33				1	1
IL-5 family	1	1	1	1	1
IL-6 family	3	3	4	7	7
IL-6 receptor family	3	4	5	7	9
Common gamma chain family	8	8	8	8	8
Common gamma chain receptor family	10	12	11	12	12
Other interleukins receptors	4	4	5	7	7
*Other cytokines*					
Interferons	4	8	5	21	23
Interferon receptors	6	6	6	6	6
CSFs	4	4	3	4	4
CSF1R	1	1	1	1	1
TGFs	2	3	3	3	3
*TNF super family*					
TNFSF	9	10	10	18	18
TNFRSF	15	17	20	20	19
*Antimicrobial peptides*					
Defensins	18	17	22	39	45
*Pattern recognition receptors*					
NODL receptor family	6	6	6	22	32
RNA helicases	2	2	3	3	3
TLRs	10	10	11	10	12
**Total**	**166**	**187**	**188**	**295**	**310**

The chicken genome sequence lacks TNFSF-family members TNFSF1 and TNFSF3 [44]. Presence of these lymphotoxins controls lymph node formation in mammals [45]; however, lymph nodes are absent in birds [46]. Therefore, it was not surprising that these genes were not found in any of the three avian genomes. In contrast, lack of TNFSF2 (TNFA) was unexpected, since it is found in many fish species [47], and there are several reports of TNF-alpha-like activity in chickens [48]. A sequence homology search in the three avian species only detected TNFSF15, a close relative of TNFSF2. Loss of TNFSF1, 2, and 3 (as well as TNFSF14) in the avian lineage could explain these observations (Figure S5). Absence of specific genes from the three avian genomes further implies that particular genomic regions are intrinsically difficult to clone and/or sequence with the traditional Sanger and NGS methods.

Finally, clear differences between birds and mammals exist in the size of the pattern recognition receptor families. For example, there are only six NODLR family members in each of the three avian species, in contrast to 22 and 32 in human and mouse, respectively (Table 6 and Figure S6). These are cytoplasmic receptors that recognize a range of ligands that activate caspases, and elicit an inflammatory response. A recent analysis revealed hundreds of NODLR genes in fish [49] with homologs of all mammalian genes. It is therefore clear that NODLR genes were lost during the evolution of the avian genomes. In contrast, while similar numbers of TLRs are found in birds and mammals, evolutionary histories of gene gain, loss, and conversion are complex (Figure S7) [50]–[52]. The avian TLR1A/B and TLR2A/B genes are orthologs of mammalian TLR1/6/10 and TLR2, respectively. All three birds have lost TLR8 and 9 but retained TLR7. The avian TLR21 is the ortholog of mouse TLR13, which was lost in the human lineage, and TLR15 appears to be unique to the avian lineage.

### Transposable Elements (TEs) and Other Interspersed Repeats

Approximately 6.94% of the turkey genome consists of interspersed repeats, most of which belong to three groups of TEs, the *CR1*-type non-LTR retrotransposons, the LTR retrotransposons, and the *mariner*-type DNA transposons (Table 7 and Dataset S1). The *CR1* group of TEs is the most abundant, occupying 4.81% of the genome, which is likely an underestimate because a number of highly degenerate and low copy number *CR1*-type elements remain to be characterized. Overall, the turkey and chicken genomes are very similar with respect to repeat content and the types of predominant TEs [4],[53] with high sequence similarities between major TEs. For example, *CR1_B* in turkey and chicken share ∼91% nucleotide identity over a 2 Kbp region, the *Birddawg_I* LTR retrotransposons share ∼89% identity over a 3.6 Kbp region, and the *mariner* transposon *Galluhop* shares ∼91% identity over the entire 1.2 Kbp of the full-length element. Similar to the chicken, the *Galluhop* repeat in turkey is associated with a deletion derivative of ∼550 bp. Repetitive sequences are among the fastest evolving sequences in the genome. Therefore, the conservation of the repeat elements and sequences between the turkey and chicken is indicative of very stable genomes given a divergence time of 30–40 M years.

**Table 7:** Major repeat content in the turkey genome (also see Dataset S1).

Repeat Type	Count	Total bp (% of Genome)
*CR1* (non-LTR retrotransposon, LINE)	166,756	49,130,504 (4.81)
LTR retrotransposon	16,181	5,181,044 (0.51)
Mariner (Class II DNA transposon)	19,527	6,640,260 (0.65)
Unclassified interspersed repeats	83,060	10,010,105 (0.98)
Total interspersed repeats	285,524	70,961,913 (6.95)
Low complexity and simple repeats	200,695	7,872,500 (0.77)
Grand total	486,219	78,128,846 (7.63)

### Homology-Based Annotation of Non-Coding RNAs

#### Y-RNAs and tRNAs

The number of housekeeping non-coding RNA (ncRNA) genes is remarkably similar between turkey and chicken genomes (Table S10). Subtle differences however exist, with the most important one in the Y-RNA cluster. Y-RNAs are the RNA component of the Ro RNP particle [54] and represent a family of short polymerase III transcripts from a small gene cluster in tetrapods [55]. A BLAST search using known vertebrate Y-RNAs as query uncovered four loci in turkey, one of which appears to be an Y1 pseudogene. The remaining loci are identified unambiguously as homologs of the human Y1, Y3, and Y4 genes, with an Y5 homolog yet to be found. As in other tetrapods, the cluster is located anti-sense between the genes coding for the EHZ2 and PDIA4 genes, respectively. The following arrangement is conserved among Sauropsids: EHZ2>Y4<Y3<Y1<PDIA4, and although Y1 has been lost in the chicken, it has been retained in the turkey. Another difference between turkey and chicken is found for the tRNAs. In the turkey, 170 tRNAs are predicted, with 156 mapped to 20 amino acids, 4 of unknown isotype and 10 pseudogenes. Chicken, duck, and zebra finch all have a higher number of tRNAs, being 254, 241, and 219, respectively. The proportion of tRNAs in each tRNA-families is very similar between turkey and chicken, with the largest difference being observed for cysteine tRNA (Figure S8). The selenocysteine tRNA missing in the turkey genome sequence is present in the chicken and zebra finch genomes, suggesting it is most likely an artifact of incomplete data rather than a true loss, given the presence of likely genes for selenoproteins such as Gpx4 [56].

#### Evolution of miRNAs and snoRNAs

The availability of the turkey genome not only establishes stability of avian genomes in terms of ncRNAs but also permits a much more detailed investigation of the evolution of miRNAs and snoRNAs. No significant differences between the turkey and chicken are found in the numbers of miRNAs and snoRNAs. Among the 487 miRNAs found in the chicken, 432 are also present in the turkey. Similarly, out of the 223 snoRNAs found in the chicken, 194 are found in the turkey. The majority of the turkey and chicken snoRNAs are evolutionarily old: 132 snoRNAs appear across *Sarcopterygii*, 145 in *Amniota*. Most innovations of snoRNAs within amniotes are specific to eutheria, possibly reflecting a gain of function for this class of ncRNAs [57].

The evolution of miRNAs is quite different from that of the snoRNAs. Innovation occurs not only in *Sarcopterygii* and *Amniota* but in almost all species considered. This difference in evolution between snoRNAs and miRNAs is evident in *Galliformes* with 5 snoRNAs and 28 miRNAs chicken-specific (Figure S9). No large variation in count was found among the three avian species, turkey, chicken, and zebra finch (Table S10). In order to better understand the biological functions that may differ between turkey and chicken, the function of those 28 chicken-specific miRNA was assessed by searching for their putative miRNA targets. Micro RNA targets were searched using an approach similar to RNA-hybrid (see Methods) [58]. Analyses revealed that chicken-specific miRNA targets were statistically overrepresented in catabolic processes, homeostasis, double strand break repair, and iron metabolism. In particular, miR-1456, miR-1566, miR-1815, and miR-466 showed relatively small *p* values (Table S11). These results are in line with the GO analysis performed for genes under accelerated evolution (Table 6), where significant differences between turkey and chicken were found for cellular metal ion homeostasis, biopolymer catabolic processes, and DNA-packaging.

Similarly to protein-coding genes, non-coding RNAs in *Galliformes* are characterized by a high level of conservation given the divergence time of 30–40 M years. In fact, apart from moderate differences in the copy number of tRNAs, the aberrant Y-RNA cluster in chicken, and the new miRNAs, the ncRNA complements of turkey and chicken are very similar.

### Turkey Phylogeny

Genome projects enable the collection of large supermatrices of alignable nucleotide sequences for phylogenetic analysis. Galliform phylogeny was re-examined by collecting sequences from the turkey and chicken genomes for 42 loci. These sequences were assembled into the largest supermatrix available for the order, containing 83 galliform species representing 73 genera, with three anseriform outgroup species. With several whole mitochondrial sequences, two genomes, and repeated use in multiple studies, 37 taxa were represented by 11 or more loci, and 12 taxa by more than 20 loci, providing data-rich anchor points that bridged locus sets throughout the tree. For the turkey, the main finding was its close relationship with the Central American ocellated turkey *Agriocharis* (*Meleagris*) *ocellata* (94% bootstrap support) and the relation to the grouses within the phasianids (Figure S10). The turkey-grouse clade has been recovered in several [59]–[61] but not all previous multi-locus studies. The average bootstrap support for the nodes was high and the topology reproduced many features of previous studies, with monophyly of the megapodes, cracids, numidids and odontophorids, and polyphyly of the Perdicinae and Phasianinae within the phasianids. Grouping of an African bird (*Ptilopachus petrosus*) traditionally classified as a phasianid with the New World quails as recently observed [59] is supported, with the three loci independently reproducing this clustering. The same was true when *P. nahanii* was used instead of *P. petrosus*. Polyphyly of *Francolinus* was expected [62]; however, the implied polyphyly of *Lophura* was not.

### Conclusions

Increased throughput and decreased costs of NGS technologies facilitate cost- and time-effective sequencing of genomes. The turkey genome sequence described herein represents the first eukaryotic genome completely sequenced and assembled de novo from data produced by a combination of two NGS platforms, Roche-454 and Illumina-GAII. This genome project is a first where the majority of the production cost was invested in analysis and interpretation rather than generating sequence, and that the assembly is comparable in genome coverage to the predominantly Sanger-based sequences of the chicken and zebra finch. The sequence assigned to the chromosomes covers approximately 93% of the turkey genome. The quality of this sequence makes it a valuable resource for comparative genomics including identification of thousands of SNVs amenable to whole genome analyses.

The turkey sequence confirms and extends the previously known high synteny between the turkey and chicken genomes [3]. These two avian species are remarkably similar with only 30 predicted rearrangements (mainly small inversions) distinguishing their genomes, despite last sharing a common ancestor about twice as long ago as the common ancestor of mice and rats or humans and gibbons. Chromosome rearrangements that occurred show a trend towards more acrocentric chromosomes in the turkey than in the chicken. The stability of galliform genomes is further confirmed by the overall conservation of gene sequences and repeat families. At less than a third the size of mammalian genomes, a greater proportion of the turkey genome (∼10%) is under selective constraint versus mammals where the fraction of conserved nucleotides is approximately 5%. This also reflects the reduced percentage of the turkey genome comprised of interspersed repeats (7%).

Whereas genomes of close relatives allow for analysis of rapidly changing sequence, those of distant species help elucidate regions conserved during vertebrate evolution. Gene families present only in birds provide a broad perspective on lineage-specific evolution. For example, variation in gene content between birds and an egg-laying mammal (platypus) shows functions shared by egg-laying animals in general as well as those unique to egg-laying birds. Likewise, genes specific to mammalian characteristics such as tooth formation have been lost in avian species. Some gene families such as TLRs of the innate immune system show complex evolutionary histories of gene gain, loss, and gene conversion between mammalian and avian species.

The adaptive immune system is a relatively recent innovation peculiar to the vertebrates and provides a valuable framework for genome comparisons [63]. Genes involved in the control and regulation of the immune response towards invading pathogens are subject to strong selective pressures: the so-called “arms race” between pathogen and host. The result has been exceptional sequence divergence between the immune genes of vertebrate species, in particular those between birds and mammals [64]. Additionally, many immune genes belong to gene families that have been subject to lineage specific expansions and contractions, facilitating the evolution of new functions to combat pathogenic challenges. There are many fundamental differences between the immune systems of birds and mammals, including the major histocompatibility complex (MHC) structure [65], absence of lymph nodes in birds [46], and different mechanisms of somatic recombination in the generation of antibody diversity [66].

From an evolutionary perspective, the turkey and chicken provide an interesting case for comparative study. These two genomes have undergone intense artificial selection in recent decades for similar production traits, yet their differentially evolved genes included more functioning in transcriptional regulation in turkey, and more functioning in protein turnover and cell proliferation in chicken. Comparative genomics can provide additional insights into the response of the galliform genomes to this recent period, as well as to their longer histories of domestication. The turkey genome sequence can enhance the discovery of genetic variations underlying economically important quantitative traits, further maximizing the genetic potential of the species as a major protein source.

## Methods

### Genomic DNA Source

Vertebrate whole genome sequence assembly is aided by decreased variability in the target genome. To this end, a female turkey “Nici” (donated by *Nicholas Turkey Breeding Farms*) identified as NT-WF06-2002-E0010 was chosen for sequencing; Nici is also the source DNA for the two BAC libraries that have been characterized [12]. Nici is from an inbred sub-line (i.e., sib-mating for nine generations) originally derived from a commercially significant breeding line. From her pedigree, Nici has an increased inbreeding coefficient of 0.624 relative to the founder breeding line. As a prelude to initial genome sequencing, heterozygosity of Nici was compared with that of individuals from several breeder lines by genotyping 147 randomly distributed microsatellites [12]. Mean heterozygosity for Nici was determined to be 0.31 compared to 0.33 for other commercial birds. Further SNP genotyping results found Nici was homozygous at 293 of 333 SNPs (87.99%) compared to an average of 275 (81.73%) for birds from a Beltsville Small White flock closed for 30 years. Of note, all sequence data accumulated to date suggest that Nici is monomorphic at the MHC, typically the most polymorphic region of the genome [67]. It is noteworthy that NGS depth of coverage allowed for the use of a genome that was only partially inbred.

### Sequencing Strategy

#### Roche 454 sequencing

DNA libraries for WGS sequencing on the Roche/454 GS-FLX system were prepared using standard protocols provided by the manufacturer. Briefly, approximately 10 µg of DNA was sheared by nebulization and fractionated on agarose gel to isolate 500–800 base fragments. Paired end (PE) libraries were prepared essentially as described [68] by hydrodynamically shearing 20 µg of intact genomic DNA (HydroShear-Genomic Solutions, Ann Arbor, MI), polishing the ends, and ligation of circularization adapters. For preparation of ∼3 Kbp PE libraries, fragments were purified with AMPure™ SPRI beads (Agencourt, Beverly, MA) to yield DNA fragments of the desired size. For the ∼20 Kbp PE library, fragments were purified by gel electrophoresis and excision of the gel region from 17–25 kb and electro-eluting fragments. Linear fragments were circularized by cre-lox recombination within the circularization adapters, and the circularized DNA was randomly fragmented by nebulization. Nebulized DNA fragments containing PE were isolated by streptavidin-affinity purification with the biotinylated linker. These steps were followed by ligation of adaptors providing for subsequent amplification to increase library yield.

The WGS or PE libraries were used as templates for single-molecule PCR on 28 µm diameter beads in emulsions [69]. The amplified template beads were recovered after emulsion breaking and selective enrichment. Sequencing primer was annealed to the template and the beads were incubated with *Bst* DNA polymerase, apyrase, and single-stranded binding protein. Slurry of the template beads, enzyme beads (required for signal transduction), and packing beads (for *Bst* DNA polymerase retention) was loaded into the wells of a 70 mm×75 mm picotiter plate. The picotiter plate was inserted in the flow cell and subjected to pyro-sequencing on the Genome Sequencer FLX instrument. The Genome Sequencer FLX flows 200 cycles of four solutions containing dTTP, SdATP, dCTP, and dGTP reagents, in that order, over the cell. For each dNTP flow, a single 38 s image was captured by a CCD (charge-coupled device) camera on the sequencer. The images were processed in real time to identify template-containing wells and to compute associated signal intensities. The images were further processed for chemical and optical cross-talk, phase errors, and read quality before base calling was performed for each template bead. Raw reads were trimmed to remove adapter/linker sequences prior to use in de novo genome assembly.

#### Illumina Genome Analyzer II sequencing

Single and PE read DNA libraries for WGS sequencing on the Illumina GAII system were prepared using standard protocols and kits provided by the manufacturer (Illumina Inc., San Diego, CA). Prior to construction of each library type, approximately 5 µg turkey gDNA was sheared on a S2 focused ultrasonicater (Covaris Inc., Woburn, MA) to an average target size of 200 bp. Sheared DNA was recovered in 30 µL of elution buffer after purification through a QIAquick spin column (QIAGEN Inc., Valencia, CA), and the entire sample was processed according to Illumina's DNA library sample kits (v1). Adaptor-ligated DNA inserts (300 bp±50 bp) were recovered by agarose gel-purification. For amplification of single read libraries (PE libraries), 1 µL (2 µL) of each 30 µL eluant was enriched by 14 (12) cycles of PCR. Amplicons were again gel-purified, and then sized and quantified on a 2100 Bioanalyzer using a DNA 7500 chip (Agilent, Santa Clara, CA). Illumina flow cells were clustered with 4 pM aliquots from each appropriate library type using Illumina's single and PE read Cluster kits (v1), respectively. GA2 sequence data (approximately 40 Gbp) from 7 single reads (1×80 bp) and 1 PE read (2×76 bp) were generated using Illumina Cycle Sequencing kits (v2–v3 upgrade), and images were processed for base-calling using either GA Pipeline 1.3.2 or 1.4.0 under standard parameters. The GA Pipeline was run on a quad-processor dual-core Linux server running CentOS 5.3.

### Assembly Process

Celera Assembler release 5.3 was used to produce the assembly. The assembly process can be summarized to the following major stages:

Stage 1 (gatekeeper): input of reads and quality control

Stage 2 (overlapper): computation of read overlaps and trimming of poor quality sequence based on the overlaps

Stage 3 (unitigger): initial assembly of uniquely-assemblable contiguous chunks of sequence based on the overlaps

Stage 4 (cgw): scaffolding of unitigs based on mate pair data, followed by merging overlapping unitigs into contigs

Stage 5 (consensus): computation of consensus sequences for the contigs

There are multiple choices of the assembler modules available for overlapping and unitigging. The traditional OVL overlapper was originally designed for Sanger reads. The more advanced MER overlapper was designed to account for the homopolymer errors that are common in 454 read data. The MER overlapper is more accurate, although several times slower, on pure 454 assemblies. Surprisingly, with the combined Illumina and 454 Titanium data, the MER overlapper had no advantage over OVL, which suggests that the homopolymer errors are less pronounced in the latest Titanium data. Because BOG (best overlap graph) is more tolerant of highly variable read sizes (74 bp to 366 bp), the newer BOG unitigger was used instead of the original unitig module.

Three maps were used to produce a Combined Map (CMap) for alignment of assembled sequence to chromosomes. The CMap had 31,769 markers that mapped both to the assembly and to the turkey chromosomes MGA1 through MGA30. Maps for the sex chromosomes W and Z were not used due to fragmentary marker coverage. Instead, scaffolds that aligned only to chicken W and Z chromosomes were identified and then ordered and oriented according to the chicken coordinates.

### BAC Contig Physical Map

A detailed comparative BAC contig physical map for turkey [16] was generated based on over 43,000 BES, over 80,000 BAC fingerprints, and over 34,600 BAC locations assigned by hybridization to overgo probes corresponding to 2,832 loci [70]. Two different BAC libraries were used: CHORI-260 generated by the Children's Hospital of Oakland Research Institute and 78TKNMI generated at Texas A&M University. Comparative BAC contigs were assembled based on: (1) consistent (correct strandedness and separation distance) alignments of mate-paired BES to the chicken genome sequence (Build 2, May 2006, http://genome.ucsc.edu), (2) hybridization to unique overgo sequence probes aligned with the chicken genome, and (3) BAC fingerprint-based contigs [16]. The BAC contig physical map, along with the BES, provides a tool for aligning scaffolds from the turkey sequence to turkey chromosome regions as well as for identification of rearrangements between the chicken and turkey genomes. (Regularly updated versions of this map are available at http://poultry.mph.msu.edu/resources/resources.htm#TurkeyBACChicken, and it can also be accessed in graphical form at http://birdbase.net/cgi-bin/gbrowse/turkey09/, see Text S1.)

The current number of contigs, end sequence matches to the chicken genome and lengths are provided in Table S12. Most gaps between contigs are due to regions of low BAC density (particularly on microchromosomes MGA18 and 24–30 and on the sex chromosomes that are underrepresented in the BAC libraries and, in some cases, poorly assembled in the chicken sequence). However, some gaps are due to repetitive regions and likely sites of CNV [10]. The average size of comparative map BAC contigs on the autosomes is over 10.5 Mb with the N50 average autosomal contig size being about 31 Mb. Twelve chromosomes (MGA6, 9, 11, 12, 13, 16–21, and 26) are spanned by only a single BAC contig and another five are spanned by two contigs (MGA2, 5, 15, 23, and 30).

In addition to the previously known centric split of GGA2 to MGA3 and 6 and fusion of acrocentric MGA4 and 9 to create the metacentric GGA4, the comparative map suggests movement of more interstially positioned chicken centromeres to positions at or near the telomeres on MGA2, 7, 10, 11, 12, and 13. Although MGA3 and 7 both contain short p arms visible in the turkey karyotype [10], no evidence of centromeric breaks internal to sequences orthologous to that of the chicken were found on these chromosomes, although there are a couple of short terminal contigs on MGA7 that could comprise a very small p arm. Of course, there may also be repetitive sequences or other sequences that were refractory to assembly in the chicken sequence that may be located on p arms of MGA3 and 7 (MGA8 and 14 are difficult to resolve near the likely p end telomere due to multiple rearrangements, and microchromosomes MGA18 and 25–30 tend to be fragmented in the BAC map and less well assembled in the chicken sequence due to poor BAC coverage).

### A SNP-Based Linkage Map of the Turkey Genome

A total of 768 SNPs were genotyped on a F2 population of two genetically different commercial turkey lines that consisted of 18 full sib families with a total of 948 offspring. SNPs were genotyped using the Illumina Golden Gate assay. Of the 768 SNPs, 458 were eventually used to build linkage maps for 27 chromosomes (MGA1–17, 19–26, 28, 30) (Table S13). The linkage map was constructed with a modified version of CRI-MAP software kindly provided by Drs. Liu and Grosz of Monsanto. All markers were checked for non-Mendelian inheritance errors using the option “prepare.” Linkage maps for the individual chromosomes were constructed in a number of iterative rounds using the “build” option within CRI-MAP starting with a threshold of LOD = 5 with subsequent stepwise lowering the LOD threshold until LOD = 0.1. Closely linked markers not separated by recombination events were ordered according to their location on the chicken sequence map (build WASHUC2). The order of markers in the final map was verified using the “flips” option.

### SNVs

Strong SNVs are positions at which: (1) at least three reads support each nucleotide variant, (2) the sum of the top three quality values for each variant is at least 60, and (3) the overall depth of coverage is at most 30. For this analysis, gaps in the multiple alignments were assigned a quality value equal to the minimum quality of the flanking bases. In addition, if the SNV was an indel within a homopolymeric run, at least one Illumina read was required supporting each variant. The support and quality value thresholds should reduce chance sequencing errors to 1/1,000,000, and the 30-fold depth of coverage threshold should filter out apparent variations caused by near-identical repeats. The requirement for Illumina reads verifying homopolymer indels was used to filter out well-known 454 sequencing biases [71]. Weak SNVs are similar to strong SNVs but with relaxed thresholds. For weak SNVs, at least two reads had to support both variants, and the sum of the top two quality values for each variant had to be at least 45. The restriction on the depth of coverage was removed. As with strong SNVs, if the variant was an indel within a homopolymer, at least one Illumina read supporting each variant was required.

### Annotation of Protein-Coding Genes

Draft annotation was generated using two independent methods. First, a draft annotation of 12,206 putative protein coding loci was generated by combining evidence from multiple sources using JIGSAW [72]. Evidence for genes included spliced alignments of known proteins and mRNAs from multiple vertebrates, and expressed sequence tags (ESTs) from chicken and turkey. Ab initio gene predictions by Twinscan [73] and GlimmerHMM [74] (trained on chicken genes) were also considered by JIGSAW but with a very low relative weight, such that no gene models were based solely on ab initio predictions. The protein mappings were given the highest weight, followed by full-length mRNA alignments and then EST alignments. Proteins and mRNAs were taken from the most recent Ensembl gene builds for chicken, zebra finch, and green Anole lizard, and from the GenBank RefSeq database (the “other vertebrates” section plus mouse and zebrafish genes). Many thousands of genes and gene fragments were eliminated from the initial predictions if the computational evidence was insufficient. Second, the gene-finding pipeline at Ensembl [75], which also uses a combination of known proteins, ESTs, and cDNAs to annotate genes, was used to generate an independent set of protein-coding genes and noncoding RNAs. After combining the two gene lists, the total number of distinct protein coding loci was 15,093 plus 611 noncoding RNA genes, for a total of 15,704 genes. Some loci were identified as producing multiple distinct proteins due to alternative splicing, giving 16,217 distinct protein sequences. Orthologs between turkey, chicken, and human proteins were defined using sequence homology, phylogenetic trees, and conservation of synteny. Homologous pairs and orthology type are available from the version 57 Ensembl Compara database (http://e57.ensembl.org). It was assumed that all 1∶1 orthologs were correct and were used to define conserved syntenic regions. Further orthologs were then defined from the one-to-many and many-to-many relationships, if the homologs mapped to a conserved syntenic region. This allowed for a 7%–8% increase in the number of defined orthologs for all species (Table S14).

### Three-Way Avian Genome Alignment

Multiple (three-way) alignments were built on the turkey, chicken [4], and zebra finch [7] genomes using Pecan [33]. Pecan is a global multiple sequence aligner that assumes no major rearrangements in the input sequences. Thus, sets of collinear segments were defined before aligning the sequences. Searches were based on the turkey-chicken and chicken-zebra finch pairwise BLASTZ-net alignments [76]. The genomes were repeat-masked using RepeatMasker (www.repeatmasker.org), followed by BLASTZ analysis, to find all highly similar regions, which were grouped in chains using the axtChain software and refined using the netChain software [77]. GERP [34] was used to get both per-base conservation scores and conserved elements.

### Lineage Specific Expansion/Contraction of Protein-Coding Gene Families

#### Assignment and comparison of gene families

The gene family annotation from Ensembl version 56 (http://e56.ensembl.org) was downloaded and the following procedures were conducted to assign all the 12,206 predicted turkey genes to gene families. First, for the turkey genes that have a best match to non-turkey genes, family annotation of the non-turkey reference genes was automatically propagated to the turkey genes, i.e., turkey genes were assigned to the families to which their non-turkey reference genes belong. Altogether, 12,054 turkey genes were assigned to gene families in this manner. Next, protein sequences of the remaining turkey genes were extracted and used to BLAST against the chicken protein database. Manual inspection of the BLAST results revealed an additional 77 turkey genes with relatively good sequence homology to the chicken genes (i.e., higher than 75% similar with alignments covering more than 25% of the sequence), and these were assigned to the corresponding chicken gene families. Finally, 26 of the remaining turkey genes were assigned to gene families through manual search of gene description keywords in the NCBI HomoloGene database and BLAST of reference genes against all available sequences at GenBank. The remaining 46 genes could not be assigned to any Ensembl family (most were annotated as predicted genes) and were discarded from the subsequent analysis.

#### Comparison of gene family copy numbers between turkey and other species

Rates of change in gene family size were computed as previously described [78]. Briefly, copy numbers for 15 species including human, macaque, chimpanzee, mouse, rat, dog, pig, cow, opossum, zebrafish, fruitfly, lizard, fugu, chicken, and zebra finch were computed for each gene family based on the Ensembl annotation. Copy numbers in human were used as reference to calculate the rate of copy number change (R) for each gene family using the equation: 
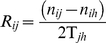
 where *Rij* is the observed rate of family size change in family *i* of species *j* relative to the human, and *n_ij_* is the number of genes in family *i* of species *j*. When *j* = *h*, the species is human. T*jh* is the divergence time in million years between species *j* and human. Divergence times were obtained [79], and for each family, rate patterns were generated by sorting the species based on *R* values and then clustering the gene families with the same pattern into rate pattern groups (RPG). Turkey/chicken-specific RPGs, which contain gene families that are either expanded or contracted in turkey/chicken as compared to all other non-bird species, were examined first. Then the zebra finch was added and bird-specific (turkey/chicken/zebra finch) RPGs, which contain gene families that are either expanded or contracted in the three birds as compared to all other non-bird species, were examined.

### TEs and Other Interspersed Repeats

Searches were combined based on similarities and de novo repeat analysis approaches. For de novo repeat analysis, RepeatScout [80] and LTR_Struc [81] were used under default conditions. Overall, 944 repeat elements were identified, many of which were redundant. LTR_Struc uncovered three LTR retrotransposons that had both LTRs and the reverse transcriptase domain. During similarity searches, known chicken repeats (Repbase Update, http://www.girinst.org) as well as representatives of different types of TEs were used as query. Repeats identified by RepeatScout were classified by comparison with known chicken repeats as well as with representative LTR retrotransposon protein sequences, non-LTR retrotransposon (or LINE) protein sequences, and DNA transposases. Whenever possible, the chicken homolog was used to assign names for the turkey TEs.

### Homology-Based Annotation of Non-Coding RNA (ncRNA) Genes

#### RNA folding and co-folding

Various tools from the ViennaRNA package [82] were used to determine the putative structure as well as the function of the reported ncRNAs. In particular, structural features were derived from RNAfold, RNAcofold, RNAalifold, and RNAduplex, while putative ncRNA-RNA interactions were predicted with RNAplex and RNAplfold. Query sequences were obtained from NCBI and Rfam [83].

#### Annotation of tRNA, miRNA, and snoRNA genes

Putative tRNA genes were annotated with tRNAscan-SE [83] using default parameters. The repetitive structure of the rRNA operons causes substantial problems for genome assembly software [84]. In order to obtain a conservative estimate of the copy number, only partial operon sequences that contained at least two of the three adjacent rRNA genes were retained. These settings, however, did not allow finding any rRNA loci. Interestingly, a provisory rRNA annotation from Ensembl also could not predict any rRNA, indicating that rRNAs have been excluded completely from the current assembly.

A great variety of C/D-box and H/ACA-box snoRNAs were reported during the last few years. Due to the fact that snoRNA sequence similarity is much higher in closely related organisms, a step-wise approach was used for finding all homologs among members of the vertebrate families. Starting with all reported snoRNAs in chicken, the genomes of the turkey and zebra finch, as well as other vertebrates [human, mouse, platypus (*Ornithorhynchus anatinus*), duck (*Anas platyrhynchos*), lizard (*Anolis carolinensis*), frog (*Xenopus tropicalis*), and zebrafish (*Danio rerio*)], were searched for all reported snoRNAs and miRNAs in vertebrates using BLAST. Search parameters were used as in Ensembl (-W 8 -r 1 -q -1 -G 2-E 1 -a 4). A total of 607 snoRNA sequences were retrieved as reported by Shao et al. [85] as well as from snoRNA-LBME-db [86]. All identified snoRNA homologs in one organism were added to the query set for the next related organism, according to the phylogenetic tree, in order to improve the results in the following BLAST step. BLAST hits were accepted as homologous snoRNAs with a sequence identity higher than 85% and a minimal length of at least 90% of the given query, the E-value cutoff was 10^−3^. Each snoRNA was checked if it could be mapped to one of the RFAM entry by using the Perl script rfam scan.pl provided by RFAM.

All 1,993 known pre-miRNA sequences found in the BLAST searches were downloaded from the mirBase database version 14. Duplicate sequences were removed leading to a total of 1,468 miRNA precursors used as query sequence. Seven additional miRNAs from a provisory Ensembl annotation were further incorporated. Similar to the snoRNA annotation, homologs were identified with BLAST. All identified miRNA homologs in one organism were added to the query set for the next related organism, according to the phylogenetic tree, in order to improve the results in the following step. BLAST hits were accepted as homologous miRNAs with a sequence identity higher than 85% and a minimal length of at least 90% of the given query; the E-value cutoff was 10^−3^. The procedure was iterated until no further miRNA homologs were found. Each miRNA was further checked for mapping to one of the RFAM entry by using the Perl script rfam scan.pl provided by RFAM.

Putative miRNA targets were searched using RNAplex. For each gene, the largest 3′ UTR region was selected and the local accessibility was computed with RNAplfold. For each miRNAs, interactions with a seed from nucleotide 2 to 6 were selected and with an interaction energy which was among the 30% highest interaction energies. GOEAST [87] was used to investigate putative functional enrichment of the targets.

### Phylogenetic Analyses

#### Ortholog set preparation and d_N_/d_S_ estimation

Predefined simple 1∶1 ortholog sets of human, mouse, dog, opossum, zebra finch, and chicken were retrieved from the OPTIC database [88]. Defining 1∶1 ortholog sets between turkey and chicken genomes was performed using the *Mestortho* program [89]. Protein sequences of the orthologous genes were aligned with ClustalW [90]. Using *pal2nal* [91], the protein sequence alignment and the corresponding mRNA sequences were converted into codon alignments. The *codeml* option of *PAML*4.2a [92] was used to estimate *d_N_*, *d_S_*, and *d_N_*/*d_S_* (ω) ratio using estimated κ and F3X4.

#### Evolution in avian lineages compared with mammals

The non-synonymous/synonymous rate ratio ω = *d_N_*/*d_S_* indicates the selective pressure on the protein. A less stringent and phylogenetic topology-free alternative was used to detect accelerated molecular evolution called identifying “differentially evolved genes” (*Devogs*). Briefly, ω ratios for each gene between turkey and chicken were compared with all six pairwise ω ratios for mammals using the student *t* test. When the log transformed ω ratio for a gene between turkey and chicken is significantly higher than that found between mammals, it indicates accelerated evolution of the gene between turkey and chicken lineage compared with those in mammalian lineages and vice versa. The accelerated genes in turkey and chicken lineages can be further classified into two groups, which are genes accelerated in turkey lineage and chicken lineage. Paired *t* test statistics of log transformed ω ratio between turkey-mammals and chicken-mammals was performed to identify significantly accelerated genes in each avian lineage. To correct for multiple hypothesis testing, adjusted *p* values were obtained using the Benjamini and Hochberg false discovery rate procedure [93] and identified significantly accelerated genes at the level of adjusted *p*<0.05.

#### Gene enrichment analysis using GO terms

Gene enrichment analysis of GO terms was performed using the *DAVID* functional annotation program [94]. Over-representation statistics for every possible GO term in the *Devog* between-turkey-mammal or within-mammal with respect to the given orthologous gene sets of the seven species were calculated using EASE [95]. To correct for multiple hypothesis testing, adjusted *p* values were obtained using the false discovery rate procedure [93] and identified significant GO terms at the level of adjusted *p*<0.05. Hierarchical clustering of over-represented GO terms was conducted with a dissimilarity matrix as defined by Kosiol et al. [96]. Specifically, two GO terms, X and Y, have dissimilarity
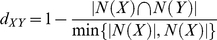
where *N* (C) denotes the set of *Devog* assigned to GO category C. Only the *Devog* information was used and did not include the background gene information of GO terms for clustering because it would give prominence to GO terms information with respect to the *Devog* background. The *hclust* function in the R statistical package (www.r-project.org) was used with the “average” option for hierarchical clustering.

### Phylogenetic Comparison of Immune Genes

Sequences other than those derived from the turkey genome project were collected from NCBI (www.ncbi.nlm.nih.gov) using BLAST [97], retrieved through searches in Ensembl (www.ensembl.org), and identified by BLAT searches on the UCSC Genome Bioinformatics database (genome.ucsc.edu). ESTScan v. 2 [98],[99] was used to correct predicted coding regions for frameshift and other sequencing errors. The alignments of amino acid and coding sequences used for the analysis of gene conversion and the construction of phylogenetic trees were generated with MUSCLE v. 3.7 [100]. Gene trees reconciled with species trees were calculated using TreeBeST v. 1.9.2 [101] and trees were visualized using Archaeopteryx [102]. Perl scripts and modules from Bioperl [103] were used to manipulate sequence and phylogenetic data.

### Maximum Likelihood Estimation of Phylogenetic Trees

DNA sequences for 42 high-coverage loci (11 mitochondrial) were collected from GenBank, for all *Galliformes* and for three outgroup *Anseriformes* species. One representative species of each genus was selected based on frequency of coverage, and additional representatives were chosen for the known polyphylous genus *Francolinus* and in cases of complementary locus coverage. The 86 final species were represented by 10.6 kb on average. For *Coturnix coturnix*, loci from the complete mitochondrial sequence of *C. japonica* (often considered a subspecies of the former) were used. *Northura maculosa* was classified as a cracid at NCBI but did not join the other cracids in preliminary trees; this GenBank entry was apparently a misspelling and misclassification of the tinamou *N. maculosa* and was removed from the study; GenBank was notified of the problem. For each locus, sequences were aligned and the unmasked alignments were concatenated, partitioned by gene (except that the two mitochondrial rRNA genes were fused as were the eight mitochondrial coding sequences). A maximum likelihood tree was constructed using RA×ML with the GTR-GAMMA model and 100 full bootstraps were taken.

## Supporting Information

Datasets S1 Supplemental FASTA file of repetitive sequences in the turkey genome.
(0.10 MB DOC)

Figure S1 **Distribution of regions of exclusive coverage for both sequencing platforms.** Panel (a) shows a large number of short (<20 bp) gaps in coverage by Illumina sequencing, whereas the Roche/454 coverage gaps tended to be larger as shown in panel (b). The mean sequencing gap for Illumina reads was 46 bases compared to a 72 base mean gap for Roche/454 coverage.
(0.53 MB TIF)

Figure S2 **SNP identification and estimates of nucleotide diversity across the turkey genome.**
(0.05 MB JPG)

Figure S3 **FISH confirmation of the turkey-chicken inversion rearrangement due to apparent unequal recombination between** ***NME1*** **and** ***NME2*** **orthologs on GGA18/MGA20.** CHORI-260 BACs 110F07 (GGA18 end coordinates: 4,850,650–5,056,016) and 112P09 (9,665,995–9,865,995) were labeled in green (FITC), while 96A17 (5,087,535–5,266,203) and 92G16 (9,980,713–10,142,396) were labeled with red (Enzo Red) and used for FISH analysis of chicken and turkey pachytene chromosomes, which are 14–20× more extended than mitotic metaphase chromosomes allowing for greater resolution. A view of GGA18 (left frame) affirms the arrangement predicted by the BES alignments noted above, 110F07 and 96A17 signals co-localize to generate a yellow signal halfway along the chromosome q arm, as do 112P09 and 92G16 near the q terminus. Whereas for MGA20 (right frame), the 110F07 and 112P09 BAC probes co-localize (green) as do the two red probes, indicative of the 5 Mb inversion. (Prior FISH experiments utilized the BAC probes singly or in pairs of two to ensure all probes hybridized equally well.) This inversion was previously indicated by inconsistent BAC mate pairs: CHORI-260 111D05 (5,106,305–10,099,832), 95I22 (5,109,664–10,107,855), 89F20 (5,134,762–10,035,123), 94C02 (5,157,115–10,042,702), and 95H13 (5,268,786–9,982,916) and 78TKNMI 18A07 (5,109,437–10,066,115), all of which had BES that aligned with the same strand in the chicken sequence, as expected for BACs that cross inversion breakpoints. Additional FISH, overgo mapping, and fingerprint analyses confirm the inversion and narrow the breakpoint regions to sites near the *NME1* and *NME2* orthologs (unpublished data).
(0.06 MB JPG)

Figure S4 **Evolution of the CCL gene family of chemokines.**
(0.08 MB JPG)

Figure S5 **Evolution of TNF superfamily of ligands.**
(0.05 MB JPG)

Figure S6 **Evolution of NOD-like receptor gene families.**
(0.06 MB JPG)

Figure S7 **Evolution of the Toll-like receptor gene family.**
(0.05 MB JPG)

Figure S8 **Codon usage in percent for** ***M. gallopavo*** **(turkey, blue),** ***G. gallus*** **(chicken, orange),** ***A. platyrhynchos*** **(duck, yellow), and** ***T. guttata*** **(zebra finch, green).**
(0.07 MB TIF)

Figure S9 **Lost and gained snoRNAs (a) and miRNAs (b) in different species.**
(0.24 MB TIF)

Figure S10 **Turkey phylogeny: Maximum likelihood tree of Galliformes based on concatenated, partitioned alignment of DNA sequences for 42 loci (11 mitochondrial).** Each species is marked with a two-letter abbreviation of its NCBI order (AN, Anseriformes outgroup), family (MP, Megapodiidae; CR, Cracidae; NU, Numididae; OD, Odontophoridae), or phasianid subfamily (PE, Perdicinae; PH, Phasianinae; TE, Tetraoninae; ML, Meleagridinae), followed by the number of loci used for the species. Bootstrap support percentages are shown if <100%.
(0.15 MB JPG)

Table S1 **HSA19q homologs.**
(0.19 MB XLS)

Table S2 **Annotation of turkey genes (edit date 11/10/2009).**
(4.01 MB XLS)

Table S3 **Predicted rearrangements between the chicken and turkey genomes.**
(0.04 MB XLS)

Table S4 **Summary of gene family assignments.**
(0.03 MB DOC)

Table S5 **Gene family comparisons between turkey and chicken.**
(0.14 MB DOC)

Table S6 **Summary of gene families in 16 animal species.**
(0.05 MB DOC)

Table S7 **Species-specific RPG.**
(0.03 MB DOC)

Table S8 **Enrichment test of KEGG pathway.**
(0.04 MB DOC)

Table S9 **Wilcoxon rank sum test between innate immune related genes and the other genes.**
(0.03 MB DOC)

Table S10 **Summary of homology-based RNA annotations from the sequenced genomes of turkey (** ***M. gallopavo*** **), chicken (** ***G. gallus*** **), and zebra finch (** ***T. gutatta*** **).** Where a range of numbers is given, it remains uncertain whether multiple copies in the genomic DNA are true copies of the gene or assembly artifacts.
(0.06 MB DOC)

Table S11 **Significantly enriched GO terms (** ***p*** **<0.0001) in the set of putative targets for the chicken specific miRNAs.**
(0.04 MB DOC)

Table S12 **Comparative map contig list.**
(0.20 MB XLS)

Table S13 **Turkey SNP linkage map.**
(0.14 MB XLS)

Table S14 **Summary of gene orthologs defined from sequence homology, gene trees, and conservation of synteny for Ensembl gene predictions.**
(0.03 MB DOC)

Text S1 **Turkey GBrowse and GenBank submission.**
(0.03 MB DOC)

## Abbreviations

BAC: bacterial artificial chromosome
BES: BAC end sequence
CNV: copy number variation
ESTs: expressed sequence tags
FISH: fluorescence in situ hybridization
GO: gene ontology
LINE: long interspersed nuclear element
MHC: major histocompatibility complex
miRNA: micro-RNA
ncRNA: non-coding RNA
NGS: next-generation sequencing
RPG: rate pattern group
SINE: short interspersed nuclear element
snoRNA: small nucleolar RNA
SNP: single nucleotide polymorphism
SNV: single nucleotide variant (SNP/indel)
TE: transposable element
TLR: Toll-like receptor
WGS: whole genome shotgun
