# Supplementary material for: Multi-Platform Next-Generation Sequencing of the Domestic Turkey (Meleagris gallopavo): Genome Assembly and Analysis
Source: PLoS Biol. 2010 Sep 7;8(9):e1000475. doi: 10.1371/journal.pbio.1000475 (PMC2935454; doi:10.1371/journal.pbio.1000475)
Supplement: Datasets S1 — Supplemental FASTA file of repetitive sequences in the turkey genome. (0.10 MB DOC) [file pbio.1000475.s001.doc]

**Supplemental FASTA file of repetitive sequences in the turkey genome**

>CR1_R551#LINEs R=551

ACCTCGAAGGGCTCTCAGCCGCTCCATCACAGCAGGATATCTCCAGACAGACACAACGCTCTCTCTGGGTTCACTTCCTGCGCGTCCCACAAGCGGACTGAGCCACCGCTCCCACCGCTGCCACAAGGGTGTATGAGGTAACAATGTGACATCACACCGCAGAGTCACCTCACAGAGAATGACGTGCCCCGGGCTGGGGACCAGGCAGCATGCAGGGCAACCTTGCAGGCCACAAAACACAGCACGTTTTTGTGACGCAATCGGCTCTGGGGTTCAAGTTCCATGCTGAAGCAGGGAATCACCTCAGCGGGTGAGGGAGAGCCCCTGCCAGGGAGCAGCTGCTGGGCACCGTTGCCCGGCATTGTCTTAGGGATTGGGGACTCCATGCCGGGAGGCACTGAGGCCCCCATCTGCTGCCCAGACAACCTCTCCAGAGAGCTTTGCTGTCTTCCAGGGGCACGTGTCAGAGACGTTAAGGAGACTCCACTGCATCTGATCAAACAAGA

>CR1_R58#LINEs R=58

AGGGTGCACTCAACCCCACTGTTGATGTCCCTGATGAAGGGGAAACCCAGATGCTTGTACCAACTGGGCGTAGAAGCCCTTGGGAGCAGCCCTGCGGAGGACTCGGGGATCCTGGTGGACAAAGAGCTGGACATAAGCCAGTAGTGTGCACTTGCAGCCCAGAAGGCCAACTGTATCCTGGGCTGCATTCAAAGAGGAGCGACCAGCAGGGAGAGGGAGGTGATTGTCCCCCTCGACTCGGCTCTGGTGCGGCCCCATGTGCAGCACTGCGTCCAGGCCTGGGGCTCCAGCACGGGAATGATGTGGAGCTTTTGGAGCAGGTCCAGAGAAGGGCCACGAAGATGATCAGAGGGCTGGAGCACCTCTCTTAGAAAGAAATGTTGAGGGAACTGGGATTGTTTCTGTGGGAGAAGAGAAAGCTCTGGAGATAGCTCATGACAGCCTTCCAAGACTCGAAGGGAGCGCATAAGCAGGAGGGGGCAACGGCTGTTTAGGAGGGTGGATAGCGATAGGACAAGGGGAAAATATAATGAAATGAGATTGGTCAGGTTTAGGTTGCTTTTTATGAAGCAATGTTTCACGCCCAGGATCGTGTCTCTCTTTTATGTGTGCCTTAGAATCGCTTCCAGGAGGATCTGCTCCATGATCTTTCCCGGCACAGAGGTGAGACTGACAGGCTGGTAGTTCCCTGGGTCATCCTTTCTATCCTTCTTAAAAATGGTGCTGTCACTTTTTTTCTCCTCACCAGGGACTTCACCTGACTGCCTCGACTTGTCAAATACCATGGAGAGTGGCTCGGTGACTGCAGCGGTGAATTCCCTCGGAACTCTGGGACTCATCTCGTCAGGACCCAAAGACTCCTGGATGTTTGGGTCCTCAGGTGGTCCAGAACCTGATCTCCCATGGCAGTGTGGGATGGGAAGGGGTCTGTGGGACTGAGGAAGCAATGGCAGTCCCCACCTCACCAAAGCAAAGATGAGGGCTGTGCATAATGAGCAGTTCTCAGACAAGAGGGAAGCTAAAATGGTAAAGGGTATTGGGGCCTTCTCTCGTGGCAAGTAACATGGTGCACAGAGCACAGACATCAGCCCTGTGCACCGAGCAAAGAGCACAGGTAGGGGCAAGAGGCACGGATGTACCGTAATGACTGTATGACGTCAAACAGGTCAAAAGAGAGCAGCAGAGCTGCGAAGCGCCCAAACAACGGAACAACGACACGGAGAGGTGTGTTGGTCTCGGTGGAAACAGCAAGGAAGTGGAATGTGCATTTCCCGGCATGCCCCGGAACCTCACTTTGCTGACTGGTCATTGTCTGCCCAGCAGGTAAGGGCGATGCACACCCGCCAGCGTTTACCCATTCCAGAGCCCGGCACAATAGGGTTCCCCCAGAGCTCTGAATTGCGCTTCATGGTGCCCAGAAGGCTGCCTATCCTCTTTGTTCTGTGTCTTGCTTGTCTCTGGTCTTGCACTGTTTTCACTATTACATTCTCTTGTCCTCCTATTTTTGTGTTCCTTGCCCCATTCTCTTGGCCTAATGACCCCTTGGCTTTGTGTTGCCACATTCATGTCGGCATTCAGTTTTGG

>CR1_B_a#/LINEs R=95

CGGCGGAAGGCTACTGCCAGGAAACATGTGGAGACCCAGACAGAGGTCCCCTGCACACGTAAGAGGAAGGCTGTAGCCAAGAAGAGTGTGGCGACCCAGACTGAGGTCCCAAAACAGCACGCTAGCGTTCAGGTTGCTGGCTGCAACGAGTGCCAGAGTCTGGCCCTTGAAGTGCCAGGCGATGGAGGCAACGCCTGTGTAAGGTGCGACCAGGTAAATGACCTTCTCAGTCTGGTGGCCTGCCTGAGGGAAGAGGTGGAGAGGCTGAGGACCATCAAGGACTGTGAAAAGGAAATTGACTGGTGGTGCCAGGCCCTATCGGCCCCGAGGTCCCGGCATGCAACTGAGGCTCCCTATGGAGCATGTGGCCCCCAGTCGCCTTGCAAACAGGTGACAGAGAGGAATCGACAGACAGACTGTGCTCCTGATACAGTGAGCCCCCAGTCTGCTCCTTCCCCTCACAATACTTATGTAGAACTAGGGGGTGTGGGACTGGGACAAAACTCTCTCCCAAAAGGGCAAGGTGATATCCACTACACAGTGGTGGAGCCTCTGCCCTGTTGTTGCCGAGCAGAGCAAAGCTGTTTGAGAGAGAAGGAGGGGTGGAAACAGATACCAACTCGGCGTTGTGGGCGTCCCCCCCCCAGACCATCCTCGGCTCCCCAGGTGCCTCTGCGGAATAGATTTGAGGCCCTGGAACTCGAGGGAGAGGTGTGTGCAGGTGTGGAGGGAGGCCCACCCGTGAGGGTGCCTCGGGGGAAGCGGTCAACCCCACGCCTCAAGACTTCCTCCACCCGGAAGGATAGAAGGGTGGTTGTCATAGGCGACTCCCTGCTGCAAGGAACAGAAGGCCCTATATGCCGGCCTGACCCTACCCGCAGAGAGGTGTGCTGCCTCCCTGGGGCACGGGTCAGGGACATTGCTAGGAGACTTCCCAGACTGATCTGCCCTTCTGACTACTATCCACTATTGATAGTACAGGCTGGCAGTGAAGAAGTTGGTGAGAAAAGCCTGAAGGCTATCAAAGATGACTTCAGGGGACTGGGGCGGGTAGTTGAAGGAACAGGTGTGCAGGTGGTTATTACATCTGTACCTTCAGTGGCAGGAAACGATACTGGGATGAGCCTAAAAACCCATCTCTTGAACAAATGGCTTAGGAGTTGGTGCAAACGCAGGAGTTTTGGTTTTTTTGATCATGGGGCAATTTACTCGGCACCTGGCATGATGGCTGCAAATGGAAGCAGCCTGTCTCCATGGGGAAAGAGGGTTCTAGCCGAGGAACTGGCGGGACTTATTGACAGGTGTTTAAACTAGGTATGAAGGGGGAAGGGGACAAAATGAGGACCGCTGGGACTGAGGGGGCAGTTCGAGGCTCCAAACAGAACTTGATGGGTGGAGACAGTGGAGTTCAAAGGCTTGGAGAGGGGTGGGGGGATGCTGCTTCTCTTAAGTGCCTGTATACTAATGCGCGTAGCATGGGGAATAAACAGGAAGAGCTGGAGTTCTGCGTGCGGTCGCGAGGTCATGATCTCATTGCGATCACGGAGACGTGGTGGGACAGCTCACATGACTGGAATGTTGTCATGGAGGGCTATGTGCTTTTTAGGAAAGATCGGCTGGCTAGGCGGGGTGGTGGAGTTGCTCTTTATGTGAGAGAGCAGCTAGAATGTATTGAACTTCACTTGGGGGAGAGTGATGTAGCAGTGGAGAGCTTATGGGTGAGAATCAAGGGGCAGGCTGGTAAGGGTGACACTGTTGTGGGTGTGTACTACAGGCCCCCTGATCAGGATGAGGAGGCTGATGAGGCCTTCCAAAAGCAACTGGATGTAGCATCACGTTCCCAGGCACTGGTGCTTATGGGGGACTTCAATTATCCAGATATTTGCTGGACGACCAACATGGCCAGGCAC

>CR1_R232#LINEs R=232

TTTTCCCTCATCCAGGGATTCCCGAGGCATCGGAGCAGCCCGGCACCTCTCGCGAGAGCGGCTCACGTGGCGGGGGCGTTGCCAGGGTAGCGGCTCTCCGCCCCCCCCCCCCCCCCCCCCCCTAATAAGAGCGTTGAAGAGAGCAGTGGCGTGCCACTGGCCACCCAGACGGGGAGGAGGAGTTTTGTCTCTGTGAAGAGGGTGATAAGTGCGGTTGCACCCATCAACGACCTGGTAAAAGAGCGAGGGCAGATCCAGCACTTTCAGTAACGGGGCGGAGCTGTTAGTCACACTTGGTAACGCTACTCTGATTGTGCTGGTGCTAGTGGGTCTGCTGGATTGCCATGGCTGCGTCCCGGCGGAAGGCTACTGCCAGGAAACATGTGGAGACCCAGACAGAGGTCCCCTGCACACGTAAGAGGAAGGCTGTAGCCAAGAAGAGTGTGGCGACCCAGACTGAGGTCCCAAAACAGCACGCTAGCGTTCAGGTTGCTGGCTGCAATGAGTGCCAGAGTCTGGCCCTTGAAGTGCCAGGCGATGGAGGCAGCGCCTGTGTAAGGTGCGACCAGGTAAATGACCTTCTCAGTCTGGTGGCCTGCCTGAGGGAAGAGGTGGAGAGGCTGAGGACCATCAAGGACTGTGAAAAGGAAATTGACTGGTGGTGCCAGGCCCTATCGGCCCCGAGGTCCCGGCATGCAACTGAGGCTCCCTATGGAGCATGTGGCCCCCAGTCGCCTTGCAAACAGGTGACAGAGAGGAATCGACAGACAGACTGTGCTCCTGATACAGTGAGCCCTCAGTCTGCTCCTTCCCCTCACAATACTTATGAAGAACTAGGGGGTGTGGGACTGGGACAAAACTCTCTCCCAAAAGGGCAAGGTGATATCCACTGCTCAGTGGAGGAGCCTCTGCCCTGTTGTTGCCGAGTAGAGCAAAGCTGTTTGAGAGAGAAGGACGGGTGGAAACAGATACCTACTCGGCGTTGTGGGCGTCCCCCCCCCAGACCATGCTCGGCTCCCCAGGTGCCTCTGCGGAATAGATTTGAGGCCCTGGAACCCGAGGGAGAGGTGTGTGCAGGTGTGGAGGGAGGCCCACCCGTGAGGGTGCCTCGGGGGAAGCGGTCAACCCCACGCCTCAAGACTTCCTCCACCCGGAAGGATAGAAGGGTGGTTGTCATAGGCGACTCCCT

>CR1_B_b#LINEs R=335

AAGAACTCATTCAGTACCTCAGCCTTCTCTTCATCCATTGAAGCAAGTTCTCCTTTTACATTTACCAAAGGAGGTACACTCGCTTTGGCCTGTCTCTTCTGGCCAATGTACCTGTAGAATGTCTTCCTATTGTTTTTCACATCCCTCGCCAAGTTCAGTTCTGCCTGCGCCTTGGCTTTCCTGATCCCACGTCTGCAAGTCCGGATGGCATCCCTGTATTCTTCCCAGGTGACATGCCCTTGTTTCCAGAGCTTGTACGCACCTTTCTTCGCCCTCAGCATGCCCAGCAGGTCCTTACCGAGCCATGCCGGTTTCCTACCTCCTCTGCCCACTTTCTTATTCAGAGGGATGGAGAGCTCTTGTGCTCTCAGGAAGGCATCCTTGAAGAGTAGCCAGCTCTCCTCAACATCTTTGTTTTTAAAGACAGCATCCCAGGGGATCTTGGCCAACAGTCCATTGAACAGCTTAAAGTTCGCTCTTCCAAAGTTCAGGGTCCTGACGCCACTCTTTGCCAGGCCCACATCCCTCGAGATCACGAACTCAACCAGGGCATGGTCACTGCAGCCCAGGCTGCCTCCAACCTTAACACCTTTAATGATCTCCTCCGCATTGGTGAGCAGCAGGTCCAGCAACGCTTCACCTCTGGTCGGTCTGTCCAATACCTGGACCAGAAAGTTATCATCAATGCATTCCAGGAGCCTCCTGGACCTCTTGCAGCTCGCCGTGTGGTCTTTCCAAGAGATATCCGGATGGTTGAAGTCCCCCATCAGGACAAGAGCCCGTGAGCACGAAGCCTCCCGCATCTGAAGCAAGAAAGCCTCGTCAACAGTCTCCCCTTGATCAGGTGGCCTGTAGTATACGCCTATCACCAGCTGGCCTTTGTTAGACCCATCCCTAATTCTAACCCACAGGCTCTCAACCTGTTCCTGACTGTTTCTCAGAGGGAGCTCCTCACAGTCTATCCACTCTTTGACATAGAGGGCAACTCCCCCACCCTTCCTACCTCGCCTATCCCTTCTAAAGAGCTGATACCCCTCAATGGTGGTATTCCAGTTATGGGAGTCATCCCACCATGTTTCCGTGATAGCAACAAGGTCATAACTTTCCAAGCACATCACGGTTTCCAACTCGTCCTGCTTATTTCCCAGGCTGTGTGCATTGGTATAAAGGCACTTCAGCTGGGCTTTCCGTCTCACCGCCTTTATAGAGGATCTAGGATCCCCTAGAACAGCAGCATCCCCAGCACCCCCCTGCCCCAGTCCATCCCTTTGTACTCCACCATCATCACCCATCAAGTTTGGTACGAGCCCTCAAATGACCCGCTCAACCCCAGCAGCCCTGATTTTGCCCCCTTCCCCCTTCATACCTAGTTTAAAGACCTTTCAATGAGTCCCGCCAGTTCCTCAGCTAAGATCCTCTTCCCCCTCAGAGACAGGCTACTTCCATTTGCAGCCATCAT

>CR1_R89#LINEs R=89

CCTCCTCTGCTTGATTTCTTCTGCTGGGGGATGGAGAGCCCTTGCGCTCTCAGAAAGGTGTCCTTAAAGAGCTGCCAGCTCTGTTCTGTTCCTATGTCCCTAAGGACAGTTTCCCAGGGGATCTCACCCAGCAGTTCCTTAAGCAGCCTGAAGTTTACTCTCCTGAAGCTCAGGGTCCTGACTCCGCTCTTTGCCAGGCCCACATTCCTTAAGATCACAAACTCAACCAGAGCATGGTCACTACAGCCCAGGCTGCCTCCAGTCTTAACCTCTTTAATGATCTCCTCTGCATTGGTGAGCACCAGGTCCAGTAACGCTTCACCTCTGGTTGGTCTGTCCAATACCTGGACCAGGAAGTTATCATCAATGGACTCCAGGAGTCTCCTGGATTGCTTGCAGCCCGCCATGTTGTTTTCCCAGCAGATATCCGGGTGGTTGAAATCCCCCATCAGGATGAGAGCCTGTGAGCATGATGCCTCTTGCAGCTGAAGCAAGAAGGCCTCATCAACAGGCTCCCCTTGATCAGGTGGCCTGTAGTAGACCCCAACCACTAGATGTCCTTTACTGGTCTGATCCTTAATTTTAACCCACAAGCTCTCAACCTGTTCATGGCTGTTTCTCAGAGGCAGCTCTTTGCAATCTATCCACTTCTTAACATAGAGGGCAACTCCCCCACCCCTCCTACCCTGCCTATCCCTTCTAAAAAGCCTGTAGCCCTCAATCATGGTATTCCAGTTATGTGATTCATCCCACCATGTTTCCATGATAGCAATTAGGTCATAGTTTTCCAATTGCACCACGGTTTCCAACTCCTCCTGCTTATTTCCCATGCTGTGTGCATTGGTATAGAGGCACTTCAGCTGGGCT

>CR1_R85#LINEs R=85, overlap with R=138

GTGATATGAGACAACCAGCATGGCTTCACCAAGGGCAAGTCCTGCCTGACCAACCTAGTGGCCTTCTATGATGGTGTAACTGCATCAGTGGACAAGGGAAGAGCCACTGATGTCATCTATCTGGACTTCAGTAAGGCCTTTGACACAGTCCCCCACAACATCCTTCTCTCCAAATTGGAAAGATATGGATTTGATGGGTGGACTGTTCAATGGATGAGGAACTGGTTGCAAGATTGTACCCAGAGAGTGGTGGTCAATGGCTCAATGTCTGGATGGAGATCAGTGATGAGTGGTGTCCCTCAGGGGTCAGTACTGGGACCGATACTCTTTAATATCTTCATCAGTGACATTGACAGTGGGATTGAGTGCACCTTCAGCAAGTTTGCAGATGACACCAAGCTGTGTGGTGCAGTCAACATACCTGAGGGATAGGATGCCATTCAGAGAGACCTAGACAGGCTGAAGCAGTGGGCCCAGGTGAACCTCATGAGGTTCAACAAATCCAAATGCAAGGTCTTG

>CR1_R2#LINEs R=2

AGGTTGGAAAAGACCTCTAAGATCATCCAGTCCAACCATCAACCTATCACCACTATTCCCACTAAACCATGTCCCTCAGTACCACATCTACATGTTCCTTGAACACCTCCAGGGATGGTGACTCCACCACCTCCCTGGGCAGCCTGTTCCAGTGCCTGACCACTCTTTCAGAGAAGAAATTTTTCCTAATATCCAACCTGAACCTCCCCTGGTGCAACTTGAGGCCATTCCCTCTCGTCCTATCACTAGTTACATGGGAGAAGAGGCCGACCCCCACCTCACCACAACCTCCTTTCAGGTAGTTGTAGAGAGCAATAAGGTCTCCCCTGAGCCTCCTCTTCTCCAGACTGAACAATCCCAGTTCCTTCAGCCGCTCCTCATAAGGCTTGTGCTCCAGACCCCTCACCAGCTTTGTTGCCCTTCTCTGGACCTGCTCCAGGGCCTCAATGTCTTTCTTGTAGTGAGGGGCCCAAAACTGAACACAGTACTCGAGGTGCGGCCTCACCAGTGCTGAGTACAGGGGGACAATCACTTCCCTGCTCCTGCTGGCAACACTATTTCTGATACAAGCCAGGATGCCATTGGCCTTCTTGGCCACCTGGGCACACTGCTGGCTCATGTTCAGCCGACTATCCATCAATACACCCAGGTCCATTTCCTCCAGGCAGTCTTCCAGCCACTCCTCCCCAAGCCTGTAGGGTTGCCTGGGGTTGTTGTGACCAAAGTGCAGGACCCGGCACTTGGCCCTGTTGAAACTCATCCAGTTGGCTTCAGCCCATCGATCCAGCCTATCCAGATCCCTCTGTAGGGCCTTTCTACCCCCAGGCAGATCAACACTTCCTCCCAACTTGGTGTCATCTGCAAACTTACTGAGGGTGCACTCAATGCCCTCATCCAGATCATCAATAAAGATATTGAAGAGGACAGGCCCCAGCACCGACCCCTGGGGAACACCACTCGTGACCGGTCGCCAGCTGGATTTAACTCCATTCACCACAACTCTCTGGGCCCGGCCATCCAGCCAGTTCTTTACCCAGCCAAGAGTGTACCTGTCCAAGCCACGGGCAGCCAGCTTCTCCAGGAGAATACTGTGGGAGACAGTGTCAAAGGCTTTGCTGAAGTCTAGGTAGACTACATCAACAGCCTTTCCCTCATCCACCAGGCAGGTCACCTGATCATAGAAGGAGATCAGGTTGGTCAAGCAGGACCTACCTTTCGTAAACCCATGCTGACTAGGCCTGATCCCCTGGTTGTCC

>CR1_F_b#LINEs R=68

AGAGCAATAACGTCTCCCCTGAGCCTCCTCTTCTCCAGACTGAACAATCCCAGCTCCTTCAGCCGCTCCTCATAAGGCCTGTGCTCCAAACCCCTCACCAGCTTTGTTGCCCTTCTCTGAACACGCTCCAGGGCCTCAATGTCTTTCTTGCAGTGAGGGGCCCAAAACTGAACACAGTACTCGAGGTGCGGCCTCACCAGTGCTGAGTACAGGGGGACAATCACCTCCCTGCTCCTGCTGGCAACACTGTTTCTAATGCAAGCCAGGATGCCATTGGCCCTCTTGGCCACCTGGGCACACTGTTGGCTCATGTTCAGCTGAGCATCAACCAACACCCCCAGGTCCATTTCCTCTACACAGTCATCCAGCCACTCAGCCCCAAGCCTGTAGCGTTGCATGGGGTTGTTGCGGCCAAAGTGCAGGACCCGGCATTTGGCCTTGTTGAACCTCATCCCATTGGCTTCAGCCCAGCTATCCAGCCTGTCCAGATCCCTCTGTAGGGCCTCGCTACCCCCAGGCAGATCAACACTTCCAGCCAACTTGGTGTCATCTGCAAACTTACTGAGGGTACACTCAATGCCCTCATCCAGGTCATCAATAAAGATATTGAACAAGATAGGCCCCAGCACCGACCCCTGGGGAACACCACTCATAACCGGTCACCAGCTGGACTTAACTCCATTCACCACCACTCTCTGGGCCCGGCCCTCCAGCCAGTTCCTTACCCAGCCAAGAGTGTACCTGTCCAAGCCACGGGCTGCCAGCTTCTGCAGGAGAATACTGTGGGAGACAGTGTCAAAGGCTTTGCTGAAGTCTAGGTAGACTACATCAACAGCCTTTCCCTCATCCACCAGACGGGTCACTCAATCATAGAAGGAGATCAGGTTGGTCAAGCAGGACCTGCCTTTCATGAACCCATGCTGGCTAGGCCTGATCCCCTGGTTGTCCCGCACATGCCGTGTGATCTCCCTCAAGATAATCTGCTCCATAACCTTCCCTGGCACCGAGGTCAGGCTAACAGGCCTGTAGTTCCCCGGATCCTCCTTACGACCCTTCTTGTAGATGGGAGTCACATTGGCAAGCCTCCAGTCTTCTGGGACCTCTCCAGTCAACAAGGAGCGCTGATAGATGATGGAAAGTGGCTCGGCTATCACCTCTGCCAGCTCCCTCAGCACTCTCGGGTGGATCCCATCCGGCCCCATGGACTTGTGGCAGTCCAGTTGGAGTAGTAGGTCTCTGACTGTTTCCTCCTGAATCGTGGGGGGTTTATTCTGCTCCCCATCCGAGACTTCCAGGTCAGAGAGTAGAGTACCCTGAGGATAACTGGTCTGACTTTTAAAGACAGATGTAAAGAAGGCATTGAGAACCTCAGCCTTTTCCTTATCCTCAGTGGTCACATTCCCAGCCTCATCCAGTAAAGGATGGAGATTCTCCTTAGTCCTCCTCTTACTGTTAATATATTTGTAAAAGAGTTTTTTGTTCTCTTTTACCCCAGTGGCCAAGTTGAGTTCAAGCTGGGCTTTTGCCTTTCTAATTTTCTCTCTGCATATCTTAACAACTTCTTTGTACTCTCCCTGAGTTGCCTGTCCCTTCTTCCACAGGAGGTAGATTCTCTTTTTCTCCTGGAGCCTCAAGAATAGCTCCCTATTCATCCACGCCGGTCTTCTTCCCCGCTGGCTCATCTTGTGGCACAGGGGGACAGCCTGCTCCTGCGCCTTTAAGACTTCCTTCTTGAAGAGCAACCAGCCTTCTTGGACCCCTTTACCCTTCAGGACTGAATCCCAAGGGACTCTCCCTACCAGTGTCCTGAACAATTCAAAGTCCGCCCTCTGGAAGTCCAAGGTAGCAGTTTTGCT

>CR1_F_d#LINEs R=11

AGAATCATAGAATCATAGAATTGCTCAGGTTGGAAAAGACCTTAAAGATCATCAAGTCCAACCACAACCTAACCATACTACCCTAACTCTAACAACCCTCTGCTAAATCATGTCCCTGAGCACCACATCCAAACGGTTTTTAAACACATCCAGGGATGGTGACTCAACCACCTCCCTGGGGAGCCTATTCCAGTGCTTAACAACCCTTTCTGTAAAGAAGTTTTTCCTGATATCCAACCTAAACTTACCCTGGCGCAACTTGAGGCCATTTCCCCTCGTCCTGTCACCTGTCACCAGTGAGAAGAGACCAACCCCGCTCTCACTGTAAGCACCTTTCAGATATTGGAAGAGAGCAATAAGGTCTCCCCTCAGCCTCCTTTTCCCCAGACTAAACAGCCCCAGTTCCTTCAGTCTCTCCTCATAGGGCATATTCTCCAAGCCCTTCACAAGCCTTGTTGCCCTTCTTTGGACCTGCTCCAGCACCTCCATGTCCTTTCTGTACTGAGGTGCCCAAAACTGAACACAGTACTCGAGGTGAGGCCTCACCAATGCCGAGTACAGGGGCAGGATGACTTCCCTAGTCCTGCTCACCACACCATTCCTGATACAAGCCAGGATGCCATTGGCCTTCTTGGCCACCTGGGCACACTGCTGGCTCATATTCAGCCGACTGTCCATCAGAACACCAAGGTCCCTTTCCATCAGGCAGCTTTCCAGCCACTCCTCCCCAAGCCTGTAGGGTTGCCTGGGGTTGTTGTGACCAAAATGCAGGACCCGACACTTGGCCCTATTGAAACTCATACCGTTAACCTTGGCCCATCGATCCAGTCTGTCCAGGTCCCTCTGTAGTGCCCTTCTCCCCTCTGGCAGATCAACACTCCCTCCCAACTTGGTGTCATCTGCAAACTTACTGAGGGTGCACTCAATCCCCTCATCAAGATCATCAATGAAGATGTTAAATAGAAGTGGCCCCAGTACCGAGCCCTGGGGGACACCACTTGTGAC

>CR1_R223#LINEs R=223

GGCCTCCAACCTTTACATCCCCCACCAGACCTTCTCTGTTAACAAACAGCAGGTCCAAGATATTGCTTCCCCTCGTTGGCTCCCTCACCAGCTGTGTCAGGAAGTTATCTCCCACACACTTTAGGAACCTCCAGGACTGTTCCCTGTCAGCTGTATTATAGTTCCAGCAGATGTCTGGGAAGTTGAAGTCCCCCACCAGAAGAAGGGGGAGTGACCTAGAGACCTCACCCAACTGTTTATAAAGCATCTTGTCCACCTCTTCATCCTGGGTGGGTGGCCTGTAGCAGACTCCCACAATAACATCAGTCTTGTTGGCCTTTGCTTTTATTCTGATCCATATGCTCTCAACCCTATCATCAACATTATTAATTTCCACACATTCATAGTCTTTTTTAACATAGAGAGCTACACCACCACCTTTCCTACCCAGCCTGTCTCTTTTGAAAAGCCGGTAGCCCTCCATCACTGCACTCCAGCTGTGGGAGTCATTCCACCACGTTTCCATGATGGCAACTATATCATGACTTTCCCAACACACAATGGCCTCCAGCTCCTCCTGTTTATTACCCATGCTGCGTGCATTAGTGTAAAAACACCTGAGCTGGGCCTCCTTTTGAGTGGCAGTAGCCCTAATACCCTCATGACCATACTCAGGTGTTTCCACAGCCACCAACCTCACACCTGCCCTCATGTTCTTCCTACAAAAGGGTGAGTCATCCTCCTCCTCCACCAAGACACAAGACCATGGGATCTTACTAGCACATCCCTCTCCCACAAGCACAGGCACGTTACATACAGGCTCCGTACTAGTGACCCCAGTTACACCCCCACCCCCCTTTGTACCTAGTTTAAAGCCCTATCAATGAACCTTCCCAACTCCCATCCTAAGACCCCTCTCCACCAGTGGGAGAAGCCACTCCTATCAGCCGCCAGCAGGCCCAGTCTCATGTAAACAGAGCAGAGGTCAAAAAACCCAAACCCCTGCCAGGCACACCAAGCTCTGAGCCAGGTATTAATCCGTTGGCCCATCATATTTAGTCCCTCATCATTCCCTATAACTGGAGGGATAGAGGAGAACACAACTTGTGCTCCCGAACCCTTTACCTGTCGCCCCAAGGCTCTGAAGTCCTTTTTCATAGTCTTTAGGGAAGTTCTTCCTATATCATCACTACCAACCTGAAACAACAACAGGGGATAGTAATCTGAGGGCCAAACTAGGGAAGGAAGTTTCTTCCTTATATCTTTAACCCGGGCTCCCAGGAGGCAACAGACCTCCCTGTGTAGAGGGTCTGGTCTACATATTGGCCCTTCTGCTCCCTTCAG

>CR1_B_c#LINEs R=16

GGTTGGAAGGGACCTCAAGGATCATGAATCTCCAACCCCCCTGCCACAGGCAGGGCCACCAACCTCCACATTTAATACTAGACCAGGCTGCCCAGGGCCCCATCCAATCTGGCCTTGAACACCTCCAGGGATGGGGCATCCACAACCTCTCTGGGCAGCCTGTTCCAGCACCTCACCACTCTCTCTGTAAAGAAATTCCCCCTGACATCCAACCTAAATCTTCCCTCCCTCAACTTAAAACCATTTCCCCTTGTCCTGCTGTTATCTACCCTTTCAAAGAGTTGATTCCCCTCCTGTTTGTAGGCTCCCTTTAGGTACTGAAAGGCTGCAATGAGGTCACCCCACAGCCTTCTTTTCTCCAGGCTGAACAAGCCCAGCTCCCTCAGCCTGTCTTTGTAGGGGAGGTGCTCCAGCCCTCTGATCATCTTTGTGGCTCTCCTCTGGACCCTCTCCAACAGCTCTCTGTCTTTCTTGTACTGGGGGCTCCAGACCTGGACACAGTACTCCAGATGGGGCCTCACAAGAGCAGAGTAGAGGGGGACAATCACCTCCCTGTCCCTGCTGGCCACCCCTCTTCTGATGGAGCCCAGGATACCATTTGCTTTCTGAGCTGCAAGAGCACACTGCTGGCTCATGTTAAGCTTTTCATCCATCAAGACCCCCAGGTCCTTCTCTGCAGGGCTGCTCTCAAGGACTGCTCCTTCCAGTCTGTATAGATGCCTGGGATTCCTCCGGCCCAAGTGCAAAACCTTGCACTTTGCTGTGTTGAACCTCATTAGGTTCACCCGGGCCCACCTTTCAAGCCTGTTGAGGTCCCTCTGAATGGCATCCCTTCCTTCCACCGTGTCAACCGCACCACTCAGCTTGGTGTCATCAGCAAACTTGCTGAGGGTGCACTCGATTCCATCATCGATGTCATTGATAAAGATGTTAAAGAGCACCGGTCCCAAGACAGACCCCTGGGGGACACCGCTCGTTACCGGCCTCCACCTGGACATAGAACCATTGATCACCACCCTCTGTCTGCGGCCTTCCAACCAATTCCTTATCCATCGAATAGTCCACCCATCAAATCCATATCTCTCCAATTTGGAGATAAGGATGTGGTGGGGGACCATGTCAAAGGCCTTGCACAAGTCCAGGTAAATGACATCGGTTGCCTTTCCCTTGTCCACCAATGCCGTCACTCCATCATAGAAGGCCACCAGATTGGTCAGGCATGACCTTCCCTTGGTGAAGCCATGCTGGCTGTCTCGGATCACCTCCTCATCTCTCATGTGCCTTAACATGTCTTCCAGGAGGATCTGTTCCATGATCTTCCCAGGCACAGAGGTGAGGCTCACCGGCCTGTAGTTCCCCGGGTCCTCCTTTCTCCCTTTCTTGAAAATGGGAGTGATGTTTCCCTTTTTCCAGTCACCGGGGACTTCACCTGACAGCCATGACTTTTCAAATATGATGGAGAGCGGCTCGGCAACCACATCAGCCAGCTCCTTCAGAACCCTGGGATGCATGTCATCTGGCCCCATAGACTTGTACACATTCAGTCTCATGAGGTGGTCTCGGACTTGCTCTGCCCTTACAGTGGGAGGGAATTTACTCCCCCAATCCCCACCTAGAGGTTCAGGGATGTGGGAGTCAGGGAAAGGAGAACTACTAGAATACTGACGCAAAGTAATGACTGAGGAAAACAACTTATTCAGTATCTCAGGCTGCTATTTCTCCGTTGAAGTTAGTTCTCCTTTTACATTTACCATAGGAGGTACACTCGCTTTGGCCTGTCTCTTCTGGCCAATGTACCTGTAGAATGTCTTCCTATTGTTTTTCACATCCCTCGCCAAGTTCAGTTCTGCCTGCGCCTTGGCTTTCCTGATCCCACGTCTGCAAGTCCGGACGGCATCCCTGTATTCTTCCCAGGTGACACGCCCTTGTTTCCAGAGCTTGTACGCACCTTTCTTTGCCCTCAGTATGCCCAGCAGGTCCTTGCTGAGCCATGCCGGTTTCCTACCTCCTCTGCCTGCTTTCTTATTCAGAGGGATGGAGAGCTCTTGTGCTCTCAGGAAGGCATCCTTGAAGAGTAGCCAGCTCTCCTCAACATCTTTGTTTTTAAAGACAGCATCCCAGGGGATCTTGGCCAACAGTCCATTGAACAGCTTAAAGTTTGCTCTTCCAAAGTTCAGGGTCCTGACGCCACTCTTCGCCAGGCCCACATCCCTCGAGATCACAAACTCAACCAGGGCATGGTCACTGCAGCCCAGGCTGCCTCCAACCTTAATGCCTTTAATGATCTCCTCCGCATTGGTGAGCAGCAGGTCCAGCAACGCTTCACCTCTGGTCGGTCTGTCCAATA

>CR1_R87#LINEs R=87

CATCAACAGTGGGGTTGAGTGCACCCTAAGGACACCAAGCTGTGGGGTGCAGTGACACAGCAGAGGGACGGGATGCCATCCACAGAGACCTCAACAGGCTGAGCAGTGGGCACAAATCCAAGTGCAGGGTCAGTAGCTGGGATGAGGGAACACCCACCACCAAAACATGCTAGGGGATGAAAGGACTGAGCGTAGTCCTGCTCCATAAGGCCTGGGAGTACTGGTGGGTGGGAGCTGGACACAAGCCAGCAGTGGGCCCTTCCAGCCCAGAAAGCCAACCGTATCCTGGGCTGCATCCAAAGATGCGCAGTCAGCGGGCGAAGGGAGGAGATCCCCATCCCTCTGCTCTGCGCTGGTCAGGCCTCACCTGCAGTGCTGCGTCCAGATGTGGAGTCCTCAGTGCAGGGAGACATGGAGCTGTGCGAGTGCATCCAGAGTACAGCCACAAACACGAACCCAAGGATGGAACCCCTCTCCTGTAAGCAAGGAGAGCTGGGCTGTTCAGCCTGGAGAAGTGAAGGCTCCAAGGGAACCTGGTAACAGCCTTTCAATACCTAAAGGAAAGAACAAGGGAAAATGGAGGTGAAATCTAAGGAAAATGTCTTGTACAGTGAGGGTGCTGAGGCAGTGGCACAGGTTGCCAGAGACATGGTGGATGTGCTGGCCCTTTCAAGGTGAGCCTGGCTCAGACTCTGAGCAACTTGATCTAGCTGTGGATGGTCAGCATGGGGGAGTCGGATCACATCGCAAACCTACCTGCATACAAGCAGCGCACCAACATGGGGTGCATGATGTTCATTACCTGCTGGACACATCTCACACCACTGACTGGAGGAGCCCAGGCAATCCTTCAACTCCATCAGGCTGCAAAATGAAATCAGAAGAGACGCAGAACATAATTTAAAACACAACAAGGATGCACACAATGCCCTTAAAAAACACCCACCGCATGGATGCAATTAGCACATAGCCCTATTAATTACAGCCATCCAGCTTGAGATCCTCCAGAAAAGCAGAGCTCACAGCTCTGAAATCAAGCAGGAATCACTCACCCCATTCTAAGGGCTCACCCCACACCCCAAAGGAAGCAGTCTCCAGATTCAGATGCTGCTCTTACACCTAGCAGCCCTTCAGTGCACTTGCGATCCCCCAAAAAGACAAGATCACAGCCCTGAAAGAAAAAAAGAAACCACTTACCCCATTCCAAGGACCAATACTCCCCCACACTCTCAAGTGAAGGAAGCAGCTTCCAGGTCCAAATACCTACCAGCCCTTAAGTACGCTCAGGGTTCCCTGCTAAAAAAGAGAGAGAGCAGCTCCACAATGGAGGAAAAAAAAAAAAAAAAAAAAAAAAACACACTCACTCAACAAAAAAAGAGACACACAACAAAGGAAGCACTCCCCAGGTTCACATGCTGCCCCTACTCTTCCCAGCCCTTCAATGGGATCAGGGAGGGGTGGAGCCTGGCTCTGCCCCACTCCACAGCCAAACTGCCCCCAGCTGTGCTCGCAGGGCTGCACCCCGCCCCAGGTGCGGGTTCAAAAAAAAAGGGGGAAAAGGAATGCCCAGGCAAAGGCAAGGGCCAAGAGAACATGGAAGGAAATGCCAAGGATGACGGCCCAGACAATACGGCAGGGAACGCCAAAACTGAATGCCGACATGAATGTGGCAACACAAAGCCAAGGGGTCATTAGGCCAAGAGAATGGGGCAAGGAACACAAAAATAGGAGGACAAGAGAATGTAATAGTGAAAACAGTGCAAGACCAGAGACAAGCAAGACACAGAACAAAGAGGATAGGCAGCCTTCTGGGCACCATGAAGCGCAATTCAGAGCTCTGGGGGAACCCTATTGTGCCGGGCTCTGGAATGGGTAAACGCTGGCGGGTGTGCATCGCC

>CR1_R217#LINEs R=217

CCACCAGACCTTTTCTGTTAACAAACAGCAGGTCCAGGATATTGCTTCCCCTTGTTGGCTCCCTCACCAGCTGTGTCAGGAAGTTATCTCCCACACACTCTAGGAACCTCCAGGACTGTTCCCTGTCTGCTGTATTATAGTTCCAGCAGATGTCTGGGAAGTTGAAGTCCCCCACAAGAACAAGGGGGAGTGACCTCGAGACCTCACCCAACTGTCTATAAAGTATCTTGTCCACCTCTTCATCCTGGGTGGGTGGCCTGTAGCAGACTCCCACAATAACGTCAGTCTTATTGGCCTTCGCTTTTATTCTGACCCATAAGCTCTCAACCCTATCATCAACATTATTAATTTCCACACATTCATAGTCTTTTTTAACATAGAGAGCTACACCACCGCCTTTCCTACCTTGCCTATCTCTTTTGAAAAGCCGGTAGCCATCCATCACAGCACTCCAGCTGTGGGAGTCATTCCACCACGTTTCCATGATGGCAACTATATCATGGCTTTCCGAACACACAATGACCTCCAGCTCCTCCTGTTTATTACCCATGCTGCGTGCATTAGTGTAAAGACACCTGAGCTGGGCCTCCTTTTGAGTGGCAGTAGCCCTAATACCCTCATGACCATACTCAGGTGTTTCCACAGCCACCAACCTCACACCAACCCTTGTGTTCTTCCTACAAAAGGGTGAGTCGTCCTCCTCCTTCACCAAGACACAAGACTGTGGGATCTTACTAGCACATCCCTCTCCTACAAGCACTGGCATGTTACATACAGGCTCTGTTCTAGTGACCCCAGTTACACCCCCACCCCCTTTCTTACCTAGTTTAAAGCTCTATCAATGAACCTTCCCAACTCCCATCTTAAGACCCCTTTCCACTAGTGGGAGAAGCTACTCCTATTGGTTGCCAGCAGGCCTGGTCTCATGTAAACAGAGCAGAGGTCAAAAAACCCAAACCCCTGCCAGGCACACCAGGCTCAGAGCCAGGTATTAATCCGTTGGCCCATTATATTTAGTCCCTCATCATTCCCTATAACTGGAGGGATAGAGAAGAACACAACTTGTGCTCCCAAACCCTTTACCTGTCGCCCCAAGGCTCTGAAGTCCTTTTT

>CR1_R24#LINEs R=24

CTGAAGGCTATCAAAGATGACTTCAGGGGACTGGGGCGGGTAGTTGAAGGAACAGGTGTGCAGGTGGTTATTACATCTGTACCTTCAGTGGCAGGAAACGATACTGGGATGAGCCTAAAAACCCATCTCTTGAACAAATGGCTTAGGAGTTGGTGCAAACACAAGAAATTTGGTTTTTTTGATCATGGGGCAATTTACTCAGCACCTGGCATGATGGCTGCAAATGGAAGCAGCCTGTCTCCATGGGGAAAAAGGGTTCTAGCCAAGGAACTGGCGGGACTTATTGACAGGTGTTTAAACTAGGTATGAAGGGGGAAGGGGACAAAATGAGGACCGCTGGGACTGAGGGGGCAGTTCGAGGCTCCAAACAGAACTTGATGGGTGGAGACAGTGGAGTTCAAAGGCTTGGAGAGGGGTGGGGGGATGCTGCTTCTCTTAAGTGCCTGTATACTAATGCGCGTAGCATGGGAAATAAACAGGAAGAGCTGGAGTTCTGCGTGCGGTCGCGAGGTCATGATCTCATTGCGATCACGGAGACGTGGTGGGACAGCTCGCATGACTGGAATGTTGTCATGGAGGGCTATGTGCTTTTTAGGAAAGATCGGCTGGCTAGGCGGGGTGGTGGAGTTGCTCTTTATGTGAGAGAGCAGCTAGAATGTATTGAACTTCACTTGGGGGAGAGTGATGTAGCAGTGGAGAGCTTATGGGTGAGAATCAAGGGGCAGGCTGGTAAGGGTGACACTGTTGTGGGTGTGTACTACAGGCCCCCTGATCAGGATGAGGAGGCTGATGAGGCCTTCCATAAGCAACTGGATGTAGCGTCACGTTCCCAGGCACTGGTGCTTATGGGGGACTTCAATTATCCAGATATTTGCTGGACGACCAACATGGCCAGGCACGCACGGTCCAGACGGTTCTTGCAGTGCGTTGAAGACAACTTTCTGATGCAGGTGGTGGAGGAACCGACGCGGGGAGGGGTGTTACTGGACCTTATTCTCACCAACAGGGATGGACTTGTTAAGGAAGTAAAGGTTGGGGGCAGTTTGGGTTGTAGTGATCATGAGATGGTGGAGTTCAAGATCCTGAGTGGAAGAAGCAAAGCAATAAGTAGGATTGCTACCCTGGACTTTAGGAGAGCCAACTTCGATCTCTTCCGGGACCTACTTGGAGCTATCCCGTGGGCTCGGGTGTTAGAAGGTAAGGGGGCCTCTGAGAGCTGGTCAGCATTTAAACAGCTCTTCTTCCAAGCTCAGGATCGGTGCGTCCCTGTGAGCAAGAAATCGGGAAAAGGTGGCAGGAGACCTGTGTGGATGAGCAAGGAGCTCATGCGCAAGCTCAAAGGAAAGAAGAAGGTCCATGAAATGTGGAAAAAGGGTCTGACCACTTGGGAAGAATATAGGAATGTAGTCAGGGCCTGCAGGGATGCAACGAGGAAGGCTAAAGCCCGCCTGGAATTGAATCTGGCTAAAGTGATAAAGGATAATAAAAAAGGCTTCTTCAAGTATGTTAACAGCAAAAGGAAAACTAGAGAGAATGTGGGCCCCTTACTAAGTGAGGGGGGGGTCCTGGTAACGGGGGATGCTGAGAAGGCAGAGATACTGAATGCCTTCTTTACTTCCGTCTTCAGTGAAAAGGCTCTCCCTCAGGAATCCCACACCCTGGAGGTTAGTGAGAGGGTCTGGGGAATGGGAGACTTCCCTTTAGTCAGGAAAGAGGACGTGCGTGAGCGCCTAGGCAGTACTAAGGTCCACAAGTCCATGGGACCTGATGGGGTGCATCCACGTGTGCTGAGGGAGCTGGCGGAGGTCATTGCCGAACCGCTCTCCATCATTTTTGAGAGGTCTTGGATAACAGGGGAGGTCCCTGAAGACTGGAGGATAGCCAATGTCACTCCGGTCTTCAAAAAGGGCAAGAAGGAAGATCCGGGTAATTATAGGCCTGTCAGCCTCACCTCTGTCCCTGGAAAGGTGATGGAACAGCTTGTGCTGGATACCATCTCCAGACAACTGGGAGAAAAGGAGGTTATCAGGAGTAGTCAGCATGGGTTCACCAAGGGGAGGTCGTGCTCGACCAACCTGGTGGCCTTCTATGATGCTGTCACATGCTGGGTGGATGGGGGAAGAGCGGTAGATGTAGTCTACCTTGATTTTAGAAAGGCATTTGATACTGTCTCCCACGACATCCTTATAACAAAGCTGAGGAAGTGTGGGATAGACGAGTGGACAGTGAGGTGGGTTGAGAACTGGCTGACTGGCAGAGCGCAGAGGGTCGTCGTTGGTGGTGCAGAGTCTGGCTGGAGACCTGTAACCAGTGGTGTCCCCCAGGGGTCTGTGCTGGGTCCGGTCTTGTTCAACATCTTCATCAATGACCTTGATGAGGGGATAGTGGCCACCCTCAGCAAGTTTGCTGATGATACGAAGTTGGGAGGATTGGCTGACACGCCTGAAGGCTGTGCTGCCATTCAGCGAGACCTGGACAGGCTGGAGAGCTGGGCAGTAAGAAACCGGATGAGGTTCAACAAAAGCAAGTGTAGGGTCTTACACCTAGGGAGGAATAATTGCATGCACCAATACAGGCTGGGGGATGAGCTGCTGGAGAGGAGCTCTGCAGAGAGGGACCTGGGCGTCCTGGTGGACGACAGGTTGGCCATGAGCCAGCAGTGTGCCCTCGTGGCCAAAAAGGCCAATGGCATTCTGGGGTGCATTAAAAAGAGCGTGTCCAGCAGGTCGAGGGAGGTGATCCTCCCCCTCTACTCTGCCCTGGTAAGGCCTCATCTGGAGTACTGTGTCCAGTTCTGGGCTCCCCAGTACAAAAAAGACAGGGATCTCTTGGAAAGAGTCCAGCGGAGGGCCACAAAGATGGTGAAGGGCCTGGAGCATCTCCCCTATGAAGAAAGGCTAAGTGAACTGGGTCTGTTTAGCCTTGAGAAAAGAAGACTGAGAGGGGACCTGATCCAGGTTTATAAATATCTGAGGTGTGGCGGCCATAGGGGTGAGGCCAGTCTCTTTTCAGTGGTACGTGGAGACAGGACGAGGGGAAACGGACATAAGCTGCAGCATAGGAAGTTTCGCACGAATGTGCGTAAGAACTTCTTCACGGTGAGGGTGACGGAGCACTGGAACAGGCTGCCCAGGGAGGTTGTGGAGTCTCCTTCTCTGGAGATATTCAAGTCTCGCCTGGACACCTACCTGTGCGACCTGGTGTAGGGAACCTGCTTTGGCAGGGGGGTTGGTCTCGATGATCTCTAGAGGTCCCTTCCAACCCCTACAATTCTGTGATTCTGTG

>CR1_R138#LINEs R=138

AAGGATGTTGTGGGGTACCATGTCAAAGGCCTTACTGAAGTCCAGATAGATGACATTAGTGGCTCTTCCCTTGTCCATTGATGCAATGACACCATCATAAAAGGCCACTAGGTTGGTCAAGCAGGATTTGCCCTTGGTGAAGCCATGCTGGTTCTCCCATATCACCTCCCTGTCTTCCATGTGCCTTAGCATAGCTTCCAGGAGGATCTGCTCCATGATCTTTCCTGGCACAGAGGTGAGACTGACAGGTTGGTAGTTCCCAGGGTCATCCTTTTTACCCTTCTTAAAAATGGGTGTGATGTTGCCTTTTTTCCAGTCACCAGGGACTTCACCTGATTGCCATGACTTTTCAAATATCATTGAGAGTGGCTTGGCAACTACATCAGCCAATTCCCTCAGGACTCTGGGATGCATCTCATCGGGACCCATAGACTTGTGGATGTTCAGGTTCCTCAGGTGGTCACAAACCTGATCTTTGCTTACAGTGGGAGGGATGTTGCTTCCCCAATCCCCTCCTACCAAACCAAACATTTGAGGGCTGTGTGGTGAGCAGTTATCAGAGAAGACCGAGGCAAAAAAGTTGTTTAGCACCTCAGCCTTCTCCTTGTCTGTTGTTACCAGCTTGCCTGTGTCACTCACTAGGGGGGTTTTCCCTCCTGGACTTTCCTTTTCTGGTTGAGGTACCTGTAGAAGCCTTTCTTGTTCTTGGTATCCCTTGCCAAGTTCAGTTCAAGCTGGGCCTTGGCTTTCCTGACCCCATCCCTACACAGCCTAGCAGCTTCCTTATACTCTTCCCACAGTACCTGTCCCTGTTTCCACTGCCTGTGCATTTTCTTCTTGCTCTTCAGTTTGACCAGCAGGTCCCAGTTCAGCCATGCTAGTCTCTTGCCTTCCTTTCCTGACTTGCTACACCTGGGGATGGAGAGCTCTTGCACACTGAGGAAAGCTTCCTTAAAGATCTGCCAGCTCTGCTCTGCACCCTTGCCCATGAGGACAGTTTCCCAGGGTGTTTTGTTGACTAACTCCTTGAAGAGCTGGAAGTTAGCTTTCCTAAAATTTAGCTTCCTAATTTTACTCTTTGCCTGTCTCATATCCCTCTGGAGCATGAACTCAACCATAGCATGGTCACTACAGCCCAGGCAGCC

>CR1_R930#LINEs R=930

TCCTCTGACCCCAATACAGCAAGACAATGCAGCTAATACACTGTGAGCTTTTGCACCAGTTTCCAAATTCAGAGCAGTGAAGCAGAGTTTCCTCTATTTTTGTATGTGAAAATTTGTATGTGCCATTTTGACTCTCTGTTCTGAGAAAATACAGCAGCACTGAGATACGAGAAAGCTGTGGTACAAGCAGAAAAGGCTGTAGTGAAGGAATCAGCATGCTGTTTTGAGGAATAGCTTTGGTGTGTTTTCGAAAAGAAAAAAAAGCCAAGTACTTTTCTTGCTAGTAGGAATAGTTCTGTCCTTGGATACAAAAACATCAAAGGATTTGCTGCTTGCAAAAACATGTTCAGCTGTTTTCATGTAGTTACACCACCTTTTAGTTTACATCTATTTTATCATCTGGATGCTGAGTAAACTTGAACCAAATGCTAGGAACGTAGGCTTCCTACTGTGCATGGAAAGAGCTTAATGTCTTAGAAAAGAACACTTTGTGAACGAAGTGAAGCAAAGGTTATCCTCAAGAGGGCAATTTGCAGGTAAACCCACTTCAGCAATAGATTATACATAGTATTTTGATTTCTGCCAGCCCATCATTGAATACTACACATATTAGGAAGAATGCAGTTTAGAAGTGTTCCTCTTCAGGCTTTTTCATCTTAGAGGTGCTATCTTCCATGTGATGCATGAAATTGATATGATCTATACTTTGCTTTTTCTGTGTATATCACAGGCATCAGTCTGAGACATCCCTAGCTCATGCTTGCTCTTCAGAATTTACTAAGATGTTTGGATCAAGAGTTTGTTAATCCTAATGTTTTTTCATCTGATGTTCAATTGGTGGTTCACTGTGGCAGATGAATCACAGTAACACTGATATGACCATTTCTTTCTACATTTTGTAGGTTTTGTGCCCACAGGCTTGGATAAATTTCATAAGGCACTGGCCATCAATATAACCTATCTGATACATCATTTGCTTTACTTTCTGATACATCATTTACATTTACTTTATCGAGTGCATCCTCAGCAAGTTTGCAGATGACACCAAGCTGAGCCTGGAAGGAAGGGAAGCCATCCAGAGGGACCTGGACAGGATGGAGAAGTGGGCCCATGAAAACCTAATGAGGTTCAATAAGGCCAAATGCAGAGAGCTGCACTTGGGCTGGGACAATCCCAGTTATTTATACAAACTGGGGGAATATCTCCTGGAGAGCAGCTCTGCAGAGAAGGACTTGTGGGTCCTGGTGGATGAGAAAC

>CR1_F_c#LINEs R=69

CACAAGAGATGAGCCTAGGGGAACGATGCTTGAGCTGGGGGTGAGGCAGATGACTCGGCTGAAGTGCATCTACACCAATGCACGCAGCATGGGCAACAAGCAGGAGGAGTTGGAAGCGATTGTGTGTCAGGCTAACTATGACCTAGTTGCTATTACCGAAACATGGTGGGACCACTCCCATGACTGGAGTGCTGTGATGGATGGCTACAAGCTCTTCAGAAGGGATAGACAAGGAAGGAAGGGTGGTGGTGTGGCCCTTTATATTAAAGACTGTTTTGATGTTGAAGAGCTTGGGGTTGGGAATGATAAAGTTGAGTGTCTATGGGTAAGGATCAGGGGGAAGGCCTATAAGGGAGACATCTTGGTGGGGGTCTGTTATAGACCGCCTAACCAGGATGAAGAGATAGATGAGGCATTCTATGAGCAGCTGGCAGAAGTTGCGCGATCGCCAGCCCTTGTTCTCATGGGGGACTTCAACTTCCCTGATATATGCTGGAAATACAATACAGCACAGAAGAAGCAGTCTAGGAGGTTTCTAGAGTGTATGGAAGATAACTTCCTGATGCAGCTGGTAAGAGAGCCTACCAGGGGAGGTGCCCTGCTAGACCTGCTGTTCACAAACAGAGAAGGACTGGTGGGAGATGTGGAGGTTGGGGGCTGTCTTGGACAGAGTGACCATGAAATGGTAGAGTTCTCTATTCTTGGTGAAGCCAGGAGGGGGAACAGCAAAACTGCTACCTTGGACTTCCAGAGGGCGGACTTTGAATTGTTCAGGAGACTAGTAGGGGGAGTCCCTTGGGATTCAGTCTTAAAGAGTAAAGGGGTCCAAGAAGGCTGGTTGCTCTTCAAGAAGGAAGTCTTAAAGGCGCAGGAGCAGGCTGTCCCCCTGTGCCGTAAAATGAGCCGGCGGGGAAGAAGACCGGTGTGGATGAATAGGGAGCTATTCTTGAGGCTCCAGGAGAAAAAGAGAATCTACCTCCTGTGGAAGAAGGGACAGGCAACTCAGAAAGAATACAAGGAAGTTGTTAAGATGTGCAGAGAGGAAATCAGAAAGGCAAAAGCCCAGCTTGAACTCAACCTGGCCACTGGGGTAAAAAGGAATAAGAAACTCTTTTATAAATACATCAACAGTAAGAGGAGGACAAAGGAGAATCTCCATTCTTTACTGGATGAGGCTGGGAATGTGACCACTGAGGATAAGGAAAAGGCAGAGGTTCTGAATGCCTTCTTTACATCTGTCTTTAAAAGTCAGACCAGTTATCCTCAGGGTACTCCACTCTCTGACCTGGTAGTCTCGGCTGGGGAGCAGACTAAACCCCCCGTGATTCAGGAGGAAACAGTCAGAGACCTACTACTCCAACTGGACTGTCACAAGTCCATGGGGCCGGATGGGATTCACCCAAGGGTGCTGAGGGAACTGGCAGAGGTGATAGCCGAGCCGCTTTCCACCATCTATCAGCACTCCTGGTTAACTGGAGAGGTCCCAGAGGACTGGAGGCTTGCCAATGTGACTCCCATCTACAAGAAGGGTCGTAAGGAGGATCCGGGGAACTACAGGCCTGTTAGCCTGACCTCGGTGCCAGGGAAGGTTATGGAGCAGATTGTCTTGAGGGAGATCACATAGCATGTGCAGGACAACCAGGGGATCAGGCCAAGCCAGCATGGGTTCATGAAAGGCAGGTCCTGCTTGACCAACCTCATCTCCTTCTATGATCGAGTGACCCGTCTGGTAGATGAGGGAAAGGCTGTTGATGTAGTCTACCTAGACTTCAGCAAAGCCTTTGACACTGTCTCCCACAGTATTCTCCTGCAGAAGCTGGCAGCCCGTGGCTTGGACAGGTACACTCTTGGCTGGGTAAGGAACTGGCTGGAGGGCCAGGCCCAGAGAGTGGTGGTGAATGGAGTTAAATCCAGCTGGCGACCGGTCACGAGTGGTGTTCCCCAGGGGTCGGTACTGGGGCCTGTCCTGTTCAATATCTTTATTGATGACCTGGATGAGGGCACTGAGTGCACCCTCAGTAAGTTTGCAGATGACA

>CR1_R172#LINEs R=172

GTACTGGAAGAGAGTAATAAGGTCTCCCCTCAGCCACCTCTTCCCCAGACTAAACAGCCCCAGCTCCCTCAGCCTCTCCTCATAGGGCTGATTTTCCAAGCCCTTCACTAGCCTCGTTGCCCTTCTCTGGACCTGCTCCAGTACCTCCACGTCCTTTCTGTACTGAGGTGCCCAAAACTGAACACAGTACTCGAGGTGAGGCCTCACCAATGCCGAGTACAGGGGCAGGATGACTTCCCTAGTCCTGCTCACCACACCATTCCTGATACAAGCCAGGATGCCATTGGCCTTCTTGGCCACCAGGGCACACTGCTGGCTCATATTCAGCCGACTGTCCATCAGAACACCAAGGTCCCTTTCCATCAGGCAGCTTTCCAGCCACACCTCCCCAAGCCTGTAGGGTTGCCTGGGGTTGTTGTGACCAAAATGCAGGACCCAACACTTGGCCCTATTGAAACTCATGCCGTTAACCTTGGCCCATCGATCCAGTCTGTCCAGGTCCCTCTGCAGTGCCCTTCTCCCCTCTGGCAGATCAACACTCCCTCCCAACTTGGTGTCATCTGCAAACTTACTGAGGGTGCACTCAATCCCCTCATCAAGATCATCAATGAAGATGTTAAACAGAAGTGGCCCCAGTACTGAGCCTTGGGGGACACCGCTCGTGACCAGCTGCCAACTAGATCCAACTCCATTGACCACGACTCTTTGGGCCCGGCCATCCAGCCAGTGCTTCACCCAACAGAGCATACACCCATTCAGACCATGGGCAGCCAGCTTCTCCAAAAATATTATAGGGGACAGTGTCAAAGGCCTTACTGAAGTCCAGGTAGACCACATCGACAGCCTGACCCTCATCCACTAAGCGGGTCACCTTGTCATAGAATGAAATCAGGTTTGTCAAACAGGATCTACCATTCATAAACCCATGCTGACTGGGCCTGATCCCCTGGTTGACCTTTAATTGGTCCATGATGGTTCCCGAGATTATTCGTTCCATAACCTTCCCAGGCACCGAGGTGAGACTGATAGGCCTGTAGCTACCAGGATCATCTTTCCGGCCCTTTTTGAAGATGGGCATCACATTTGCCAGTTTCCAATCCACTGGGACATCCCCGGTTAGCCAGGACTGCCGAAGAATGATGGAGAGTGGCTTGGCAACCACATCTGCCAACTCCCTCAGCACTCTAGGGTGCAATCCATCTGGTCCCATGGACTTGTGAGCATCCAGCTTTCGGAGCAGGTCCAAAACCATCTCATCATGGATCATGCAGGGCCTATTCTGTTCCCCATCCCTATCTACCAGCGCAGGGGGCTGTGTTCCCAGGGAGTAACAAGTCTTACTATTAAAGACTGAGGCAAAGAAGGCATTAAGTACCTCAGCCTTATCCTGATCCTTGGTCACCAAGTTGCCCTCGGCATCCAACAAGGGATGGAGATTCTCCCTAGCCCTCCTCTTGCTGTTGATGTATTTATAGAAATATTTATTGTTGTCCTTTACCTTAGTGGCCAAGTTCAGTTCTAGCTGGGCTTTGGCTTTTCTAATTTTCTCCCTGCACAGCTTCACTACATACTTGTAATCATCATAAGTAGCTTGCCCACTCTTCCAGAGACCATAAACTTTCCTTTTGCTCTTGAGTTCCAGCTAAAGGTCTCTGTTCAGCCAGACTGGCCTCCTTGCCCATTGGCTCATCTTCCATGACTTGGGGATGGTCAGCTCCTGCACCTTTAAAATAAATTCCTTAA

>CR1_F_a#LINEs R=157

TGGCAGTCTCTGAGACCACAGGTTCAACCATGGTCTCCACCAGACAGCGGGCTTGCTCTAAAAAGAATGTGGCGACCCAGACAGAGAGCCTGCCTAGAAATGTGGCTGTTCAGGTCTCTGGCTGCAGGGAGTGCCTGAGCCTGTTGCTGCCAGGGGAGGGTGGCAGGGATTCCACCTGTGTGAGGTGTGAGCAGGTGGATGATCTGCTCAGCCTGGTGGTGGAGCTCAAGGAGGAGGTGGAGAGACTAAGGACCATCAGGGAGTGTGAGTGGGAGATTGACTGGTGGAGTGACTCTCTGGCATGCCAGAGGGAAGGGTGCCAGGTAGATACCCCCAGAAAAGTGGTGGACCCCCTGCCCTGTCACAGTCGGACAGACCTGACAGATGAGGAGGATTGGAAGAGAGTCCCGGCTCAGCGTCACGGGCACCCCCCCTGCCTGCCTACCCCGCTTCCCCAGGTCCCACTAAGCAATAGGTTTGAGACTCTGGAAATTGAAGGGGAGGTGAGTGGGGAGGCAATGGAGGATCCGCCTAGGAGGGAGCCTAAGGCGAGGCGGTCACCCCCACGCCTTGAGACTGCCTCCGTCAGGAAAGAAAGAAAGGTGGTTGTGGTGGGTGACTCCCTTCTCAGAGGAACAGAGGGCCCCATATGCAGACCCGACCCAAGCCATAGGGAAGTGTGCTGCCTTCCTGGGGCCCAGGTCAGGGACATAACTAGAAAACTCCCAAAGCTGATTCGGTCAACTGATTACTTTCCATTACTCATAGTTCAGGTGGGCAGTGATGAAATCGCTCAGAGAAGCCTGCAAACTATGAAAAAAGATTTCAGGGGCTTAGGGCGTTTAGTTCAAGGAGTGGGAGCGCAAGTGATCTTTTGTTCTATACCTTCAGGGGCAGTGAGGGACACTGAG

>CR1_R0#LINEs R=0

AATGTGACTCCCATTTTCAAGAAAGGCAGTAAGGAAGATCCGGGGAACTACAGGCCTGTCAGCCTGACCTCGGTACCAGGGAAAGTTATGGAGCAAATCATCTTGGGTGAGATCACACGGCACGTGCATGGTATCCAGGGGATCAGGCCCAGCCAGCATGGGTTCATGAAGGGCAGGTCATGCTTGACCAACCTGATCTCCTTCTATGATTGAGTGACCAGTTTGGTGGATGAGGGAAGGGCTGTTGATGTCATCTACCTAGACTTCAGCAAAGCCTTTGACATGGTCTCTCACAACATTCTTCTGGCGAAACTGGCTGCCTGTGGACTGGACAGGTAGACTCTTCTTTGGATAAGGAACTGGCTGGAGGGCCGTGCCCAAAGGGTAGTGGTTAATGGAGTTAAGTCCAGCTGGAGACCGGTCACAAGTGGTGTCCCCCAGGGGTCGGTACTGGGGCCGATCTTCTTTAATATCTTTATTGATGACATAGATGAGGGGATCGAGTGCACCCTCAGTAAGTTTGCAGATGACACCAAGCTGGGAGGTGGTGTTGATCTGCCTGAGGGTAGGGAGGCCATACAGAGGGATCTAGACAGGCTGGATAGCTGGGCCGAGGTGAACGGGATGAGGTTCAACAAGGCCAAGTGCAGGGTCCTGCACTTGGGTCACAACAACCCCAGGCAACGATACAGGCTTGGGGAAGAGTGGCTGGAGAACAGCCCTGAGGAAAAGGACCTGGGGGTACTGGTTGATGATAAGCTGAACATGAGCCAGCAGTGTGCCCTTGTAGCCAAGAAGGCCAACGGCATCCTGGCCTGCATCAGAAAAAGTGTGGCCAGCAGGAGCAGGGAGGTGATTGTCCCCCTCTACTCAGCTCTGGTGAGGCCCCACCTGGAGTACTGTGTCCAGTTTTGGGGCCCTCAGTACAAGAAAGACATTGAGCTCTTGGAGCGGGTCCAGAGGAGGGCCACAAAGATGATCAGAGGGCTGGAGCACCTCTCCTATGAGGAAAGGCTGAGGGAGCTGGGATTGTTTAGCCTGGAGAAGAGAAGGCTCAGGGGAGACCTCATTGCGGCCTTCCAGTACCTGAAGGGAGCTTATAAACAGGAGGGGGAATGACTCTTTACAAGGGTGGATAGTGATAGGACAAGGGGGAATGGTTTTAAACTGAAACAGGGGAGGTTTAGGTTAGATATTAGGAAGAAGTTTTTCACACAGAGGGTGGTGAGGCACTGGAACAGGTTGCCCAGGGAGGTTGTGGATGCCCCATCCCTGGAGGCATTCAAGGCCAGGCTGGATGTGGCTCTGGGCAGCCTGGTCTGGTGGTTGGTGACCCTGCTCATAGCAGGGGGGTTGAACTAGATGATCTTTGAGGTCCTTTCCAACCCAAGCCATTCTATGATTCTAT

>CR1_R120#LINEs R=120

AGGGAGTGGAAGCAGGAATGGGTTACCTGGGAGGACTACAAAGAAGTTGTCTGAGCAGCCAGGGATCAGGTTAGGAAAGCTAAAACCCAGATAGAATTAAATCTAGCCAGGGACATAAAGGGGAACAAGAAGAAATTCTACAGGTATATCAGTGATAAAAGGAAGGCCAGGGAAGATGTGGGCCCTCTCCAGAAGGAAACTGGAGACCTGGTCACAAGGGATACAGAGAAAGCTGAGGTGCTCAATGACTTCTTTGCCTCAGTCTTCACCAGCAAGGGCTCTAGCCACACTGCCCAAGCTGCAGAAAGCAAAGGTAAGAACTGGGAGAAGGAAGATCTGCCCGCTGTAAGTGAAGATCAGGTTCGAGACCATCTAAAGAACCTGAAGGTGCACAAGTCCATGGGACCCAACAAGATCCATCCACAGGTTCTGAGGGAACTGGTGGATGAAGTTGCCAAGCCACTATCCATCATATTTGAAAGGTCATGGCAGTCTGGTGAAGTTCCCACTGACTGGAAAAGGGGAAACATAACCCCCATTTTCAAGAAGGGAAAAAAAGAAGATCCAGGGAACTACAGGCCAGTCAGTCTCACCTCTGTGCCTGGCAAGATCATGGAGCAGATCCTCCTGAAGGCCCTTCTAAGGCACATGGAAAATAAAGATGAGGTGATTAGTAGTAAACAACATGGCTTCACCAAGGGCAAGTCATGCCTGACAAACTTGGTGGCCTTTTATGATGGAGTTACAGCATCAGTGGATAAAGGAAGAGCAACTGACATCATCTACCTGGACTTGTGCAAAGCATTTGACACTGTCCCACATGACATCCTGGTCACCAAATTGGAGAAAAATGGATTTGATGGATAGACCACTTGCTGG

>Birddawg_Ia#LTR input15000_RT1_B4_L7_9_fsta.rev

GGGCTTTCATCCTTACCAGCTTCTCCAGTAGCCCTACTGTATGGGGCTACTGAATCATGGCCTGAACCTCTGATTAATCACCTGAGGTGAGCAATGAGTCAGCTACGGGAGCACAGGTGAAGGCAATACACCTGTGCTGCAGGAAGGGGTGGAGCCTGGCTCCACCTCTCTTAGAGCCATTTAAGAGCAGACTGCCAGTGGGGAAGGATCTCTTCTGGAGATCTCTCCACCAGAGTCTATCGCACAGGCCCAGGATAGGTGAGTGTCCTTTTTTCCTACTCTAGTATAATAATGACATCAGCCAAATTCCTGGATATACTGTATCATCTGTATATTCATTGATTGTACAATTGGTGAGCCAGGCAGGCAAAATATACGTTATATCATGGCTTTTTTTGTGGAATTTCTATAAATGGGTCAAGGGAGAGAACACCATTCCCCTAGATAAAATGATCCCTGGGCAGATCTCAGGGTTTCTGGAATCTCCTTACTACCAATTAGTGACCATAATTGAGAAATGGGGTCCCCTGCATTCTATGGGGAATATTTCCCTAGTACGCATGACAGAATACTCTCAAGGACTAGACAGAGACATTCTAACCACAAAAGAAAGGCAGTTAGCTGCATCCACAGTGGCATAGCCACTGTTAAATGCTTTAAATCAAGCTGAGACAAGATCAGACACATTAGAAAGTGAAAATCAGCTATTAAAAGCCCATATTGACAAACTGGAACAGGAACTTAATGTACTAACAGGAAAAGAAACAATTTTACACTCTCTAGGTCAGGTCCACGTATTTCAATAGATAGTAGTCATACCTCTGAAGTAGTGGACCTGGTTGAACAGGAAAGCAGAAACTTTAAATCAGATCTAGAAGTTCCACTTATAATAGGATGGAGTTCCAGGGAGTCCGCCATAACTCAAAAGATGAAGAAAATACAAGATAGACCAGCAGGGGTGCCCCCTGACCAATGGCCCCCTGTTGAAATGCACTTACATGTAACAGCTAGGCCATATACTCCAACTGAATTAATAGATATGGTGCAGCGATTTCGGCAGAGGCCAAGAGAGAGTGTAGCTGCATGGTTGTTTAGATTATGGGTTTCAGGGGGAGAAGTGTCATGGTTAATGGGTCAGAAGTAGCAAAGCTGGCCACCATAATGGTACACCTTGCTGTCAGGCAGAGACTATACGCTGCTGTCCAGTGTAGCAATGAGAACCATTCAATAATAGACTGATTAATGGCAGCATGCCATATAGTCTAGCCAAACAAATCAGGCATTCCATTAAACACAGGACTATGGTCCTCCATGAAGGACCTTCAAAATTATATTTGTGAATTAGGCATGAGAGAAGCGATATATGAAGACTTGCTTGATAGCCCAGATATGATAAAGTTCTCAGCAAGTATGCAAGATTTAATCTTACAACAGGCCCCTTCCCACCTATATGGGACCTTAGTTTCTATATTAAACCCTCTGGTGGCCTAGGAAGCTGTGGTTCAACAAGCTGCCCAGTTAGCAGCTGATGTGGGGGAGATGGAGCGTCTGTGCACCAGGCACAATATCCAAGTAGTAGAAGAAGCAGAGGTGCTCCCAGTAGTTAAAACCAAGCGGCCACCTATGCGGAACTCTGAGTTGGGGCCTGTAAGGGTCTCACGAAACAAATGTTTAATGACCTTATTCGGGCTGGGGTCCAATTTGCAAAGATCGACCGCCAGCCCAATCACATCCTCAGATTTGATCTTACAATCAGTGATTCCAGAACTACCCTAAGAAACCAAGGGTAAGAGTTAGAGGATGATGATGTGGAATTTTGAGGATTTCATTATTCCCAATAGAAAAAAAAAAAGTTCCCTCGGGTGTCTCAGGCTACAATGCCTGAACCACCTGAAGCAAAAGACTTGATTTTGGCCCACTTCGTGGAGTGACGAGGGATGTTAGTCCCCCTAGGGCCTCCAGACAAGACCAGAGGCCATATGTAGAGTTAACAATATATTAGTCCCAGAAAAATGTCCAGAGAGTTATGGCACATGTTGATACTGGCGCAGAAACATCAGTTATTTACGGAAATCCGAACAAATTTCATGGAGACAGAGTGATGATAGGTGGTTTTGGGGGACAAACTATTCCTGTCACCCAAACATGGTTAAAACTGGGGGTTGGGCGTCTCCCACCCAGGAGTATAAGGTATCTATTGCCCCAGTCCAGGAATACATCTTAGGCATAGATATTCTATGCAGTCTGGCTCTCCAGACAACTGTGGGAGAGTTCAGACTTCGACAGAGATGTATTAGTATCTGGGTGGTACAGGCAATATTAAGAGGCCATGTGAAACATGGGCCAATTTGCCTGCCAAAACTGCGCTGGATTACTAATGTGAGACAGTATAGAGTCCTGGGTGGGCAGGATGAAATATCAAAAATGGTGCAGGAATTAGAAAAAGTGGGTGTTATAAGACCTGCTCACAGCCTATATAATTAACCCATATGGCCAGTGTGGAAGTCGCATGGCACATGGAGAATGATGGTAGATTACAGAGAATTAAATAAAGTCACAATGCCTATTCATGCAGTTGTACCCAATAGTGCCTCCCTCATAGACACATTCAGTAGGGAGATAAAAACCTATAATTGTGTCCTAGATTTAGCAAATGCATTCTTCAGTATCCCAATTACTGAAGAATTGCAAGATCAGTTTGCATTTACATGGGGAGGCAGGCAGTGGACCTTTCAGGTCCTGCCACAGGGGTATGTGCATTCACCAACGTATTGTCACAATCTAGTGGTGCGTGACCTGGCTGATTGGAAAAAACCTGATAAGGTTAACCTGTATCACTATATTGATGATCTGCTGCTGACATCCGACTCACTGGAGGCAGTAGGGCAGGCAACGGATTCATTAACTGCCTATTTACAACAGAGGGGGTGGGCTATAAATCCTGAAAAGGTGCAAGGTCTGGGCCTGTCCGTAAAATTCCTGGGGGTGGTTTGGTCAGGAAAGACCAAAGTACTGCCCAGTGCTGTCATAGATAAGGTTCAGGCGTTCCCAGTCCCTACAACATCGAAGCAGCTGCAAGAGTTTCTAGGTGTATTAGGTTATAGGCGTTCTTTTATACGTCACCTAGTGCAGCTGCTAAGGCCACTGTAGAGACTCACAAAAAAGGGGCAGCTTTGGGACTGGGGGAAAACAGAACAGGATGCTTTCCAACAGGTGAAACTGGCAGTTAAAAAAGCCCAGGCATTGGGTATATTTGATCCTACCCTCCCAGCCGAGTTGGATGTTCATGTTACGCAACATGGCTTTGGTTGAGGCCTGTGGCAAAGCCAGAGTTCTGTTCAAACCCAAATTGGTTTCTGGTCTCGGGTTTGGCACGGAGCAGAAGGAAAATACAGTATGATTGAAAAACAGTTATTGGCTGCCTATTCGGCATTGCAGGCAGTAGAGCCAATAACTCAAACGGCTGAAGTTATAGTCAAGACCACTCTGCCGATTCAGAGGTGGGTAAAAGATCCGACCCACCTTCCTAAAACAGGGGTGGCCCACTCACAAACAGTGGCACGATGGGTCGCCTATCTCAGCCAAAGGAGTTATCTGTCTTCATCACCTTTGAAAGAAGAACTCCAGAAGATCCTAGGCCCAGTAACGTATAACAGTGATGCACCAAAAGAAATACTGGTTGCTCCACCACAGAAGAGTCCCGTTCAGGAGAGAAAATACCCTATCCCTGAAGATGCCTGGTACACAGATGGGTCCAGCAAGGACAACCCAAGCAAGTGGAGAGCAGTAGCATACCATCCTTCTACTGAAACAATCTGGTTTACTAGGGGGATGGTCAGAGCAGCCAATGGGCAGAACTGCGAGCCGTGTGGATGGTTATAACTAAGGAACACGGTGATGGTATCCTGAATATCTGTACAGATAGTTGGGCTGTGTAGCAGGGGCTCACTGTGGATTGCGGAATGGACTATCCATGCCCGGCCGATCTGGGGCAAGGCCATGTGGAATAGACATGGAATACAGTCAAACACAGGACTGTAGGTGTCTACCATGCTTCTGGTCATCAGCCCCTACAGTCACCAGGAAACAATGAAGCCAACACGCTGGCCCGAATACGATGGATTGAGAATTCACCATCTGAGAACATTGCCCGTTGGTTACATCAGAAGCTACAGCATGCGGGACACAAGACAATGTGGGCAGCTGCTAAAGCATGGGGACTGCCCATACAGCTACCTGACATCATCCAGGCATGCTGGGATTGCGACACTTGCTCCAAGATTGAGACCGAGATCGTTGCCCGAAACAACAGCCCATCTTGCTAGAGGACATAATCCTCTCCAGTGATGGCAGGTCGATTACATCGGGCCCCTCCCTCAGTCTGAGGGGGTGAGATATGCCCTGACCTGCGTCAACACTGTAAGTGGGCTACTGCAGGCCTATCCAGTGCCGAAAGCAAACCAGGCATATACCATCAAGGCACTCACCAAACTGATTTCTGCCTATGGGACACCTCAAGTCCTCGAGAGCGACCAAGGAACTCATTTTACTGGTGCACTGATACAACACTGGGCAGAAGAAAATAACATCGAATGGCAATTCCACCTGCCATATAATCCAACGGGGGCAGGCCTCATCGAATGTTATAATGGTATTCTTAAGGCTGCCCTGAAGACAGATTCCCAGTCCCTGCAGGGGTGGACAAAAAGACTGTATGAAACCTTGTGGGACGTGAATGAAAGACCCCAAGATGGCAGACCCAGCGCCCTGAGAATGTTGCATAACACATGGGCCACCCCGCTTAGGATCCAAATTACGGGCACTGATCATCAGGTAAGACCCCAGATTGGTAATGAAAATAATATTCTGCTCCCTGCCCCTGAGAATTTAGATCCTGGTACCCATAGAATAAAATGGCCTTGGAAGGTACAGGTAGGACCCAAGTGGTGTGTCCTACTTGCACCTTGGGGGAGATTGTTGGAGGCAGGAGGCTCAGTAGTCCATCCAGTAATAGGTACATGGCCTACTGATGTTGTGGTCAACACTCCAATTTTCATTGCTAAAGGGACTCCCATCATGTCCCTAGGGCAAATCAGGACACCTCCTTTGGTGCCTGATATTATTATGCAGCCGCAGACATCCGGCCAGAAAGTGTGGTACAGGCGGCCAGGACATGCCTCTGTACAAGCAGAAGTGTTGACCCAGGATAGAAACACGGCCTGTATCTTGCCCTGGAGAGCAGACCTTCCCCTCCTGGTACCTCTGAAACATCTGAATACATACTGTATTTCTCCCCGTGAGTCTTTGAGCCTTCAGGATCACTGGAAGGAACAAAATGTCATCAAAAGCATTCCAGTGTTACTGTCGGATCATGTGTAACACGCGGTGATGGTCTACATGGGACTGATGGAACACAAGACGGACCGGCACCTCACGACTGCATGTCTCGAGTAACATCATCGAGCACTGGCCCATAAAAGAATATCAACATTCATCGATTGTCACTCATCTTGAATTTTACCAACATGTGTCGCAATGAAATTGTATAAGCATCGTGGTGTATTTGATCTTTGTATTAGGAAAAGCTCACCCTTTAGTCAACCTAATCATTGGTTCACACCATGAAGGGGTGGGAGTGTACAGGGCTGCTGAATCATGGCCTGAACCTCTGATTAATCACCTGAGGTGAGCAGTGAGTCAGCCACGGGAGCACAGGTGAAGGCAATACACCTGTGCTGCAGGAAGGGGTGGAGCCTGGCTCCACCTCTCTTAGAGCCATTTAAGAGCTGACTTCCAGTGGGGAAGGATCTCTTCTGGAGATCTGCTCCACCAGAGTCTATCACGTGAGCGCAGGATAGGGTGAGTGTCCTTTTTTCCTACTCTAGTATAATAATGACATCAGCCAAATTCCTGGATATACTGTATCATCTGTATATTCATTGATTGTACACTACACTTTAGGGCTGTCCCAAGACAAGAGAAGTCTCTGCCGTCCTCCAC

>Birddawg_Ic#LTR input_4518_PT_B4_L6_7_fsta.rev

CCTATTTTATAATCTTACGTGGTGTATGGGAACTGCTGAATCGCGGCCTGAACCTCTGATTGACCACCTGAGGCGAGCAATGAGTCAGCCCTGGGAGCACAGGTGAAGTCAGTTCCCTTGTGCTGCTGAAAGGTGTGAAGCCTGGCTGCACCTCTCCTAGACCCATGTAAGGGCAGACCACCAGAGGGTAAGGATCTCTTCTGGAGATCCCTCCTCTGGAGTTTTGTGGTGAGCCCAGGATGTGGGTAAGCTTTCTGTCTATTTTCTTTTTGTTATTATAATGTGCTCTGCCAACCACTCTTGTTTATTTCATAATTATAACTTCTTTCTGTTCACTGACTATACGCTTGGTGAGCCAGACAGGCCAAATATACTAAAATTCACATTATGTCCTTTCTGTGGAATTTCTGTCAATGGGTCAGGGGGGAGAACGTTACCCCTCTAGATAAAAGGATTCCAGGGCAGATCCCAGGATTTGTGAGATCTCCCTATTATGAACTAGCAGTTATCATTGGAAAGTGGGGTCCCCTACGTGTTTTGGGAAATATCTCCCCGTACCGCATGGTAGAGTACTTTCAGGGACTTAGCAATGATATAGTGATCACAAAAGAAAGACAATCAGCTGCCTCCACAGTGGTGTGGCCGCTATTAAATGCTTTAAATCATGCTGAAGCGAGGAATAATACTTTAGAGAATGCAAACCAACCTTTGAGAGACCGTATTGAAAAGTTAGAGCAAGAACTTGGCATTTTAGCAGGGGCGAAACCAATCCTGCATCTTCCAATAGGTCAAGTTTGTAACCTTTCAATAGACAGTGACCCAACTCCTGAATCAACAGACTTCACTGATCAGGCAAATAAAAAATGTAAATTAGATCTCGAATCTCCGATTTTGACAGATCCACTAGTGGTCTGTCCTGGCATAACTCAGAAAACAACAAAAAAGTAGAGGACAGACCAGCAGGGGTGCTGCCTGCTCAGTGGCCCCCTGCCCAGACTCATTTGCACGTAACAGCCCGGCCCTACACAGCAACGGAATTGATGGATTTAGTACAGCGATCTTGGCAGGAGCCTAGAGAAAATGTGCCAGCCTGGTTATTAAGACTGGTTATTATTCTGGGGCAGAAAGTGTTTTGGTGAATGGCTCAGAAATAGCCAAGTTGGCCACCATGACTGACCATCCTGTCCTCAGGCAGAGGTTATATGCAGGTGTTCAATATCTTAACGAAAACCATTCTATAATAGAATGGTTAATGGTGGCCTGCCATATGCTATGGCCGATAAATCCGATATACCTCTACATACAGGGATGTGGTCCTCCATGGAGGACCTTCAAAATTATATCTGGGAACTAGGCGTAAGGGAAGCAATTTATGAAGATACATTTGATGGCGTTGATATGGTCAAATTCTCAGTGGGGATGAGAGACTTGATTCTACAGCAAGCCCCCTCCCATCTATATGGTACTTTGGTCTCTGTATTAAATCCCCTGGTGGCCTCAGAAGCGATAGTCCAGCAGGCTGCCCAGTTAGTGGCTAACCTGGGAGAAACAGAACGCCTGCGGTCCAGGCACAACATTCGGACAACAGAAGAAGCCGAGGTCCTGCCGCTGACTAAAACCAAGGTGCTGGCTGCTCGAGCTCCTCAATCGGAACCTGTAAGGGTCTCCCAAAAACAAATGTTTAATGATTTAATTCGGGCTGGGTTCCAGTTTGCTAAGATTGACCGCCAGCCCAACCATGTCCTTCTCCAGCTTTGGAGACAACTAAAGGACAATCAGAAATTTCAGAACTACCCTAAGAAACCTAGGGTAAGGGTTATAGAGAGAATAACAGCAACGACATGGAACCTAGAAGATTTCATTGTTCCTAATAGAAAAAAAAAGACTGCCTCAGGTGTCTTGGGCCATGATGCTTGAGCCACCAGAAGCAAAAGAATTGGCCATAGCACAATTTATGGTGTGATGAGGGATGCTAGTCCCTCTAAGGCCTCCAGACAGGACCGGAGGCCCTATGTACAATTAATGATTTATTGGTCCCGAAAAAATATTCAGAGAGTTATGGCATTAGTGGACACTGGAGCAGAAACATCAATGATCTACGGAGATCCGACCAAATCTGATGGAGACAGAGTGATGATTGGTGGTTTTGGGGGACAGACTATTCCTGTCACCCAAACATAGTTGAAATGGGGGTTTGAGCGTCTCCCACCCCGGGAGTATAAGGTATCTATTACCCCACTTCAGGAATACATCTTGGGCGTAGATACTCCATGGGATCTGGCTCTCCAGACGACTGTGGGAGAGTTCAGACTTCGACAGAGATGTATCAGTATCCGGGCAGTGTAGGCAATATTAGGAGGCCATGTGAAACATGGGCCTATTTGCCTGCTGAAACCGTGCCGGATTACTAATGTGAGACAGTATAGACTCCCGGGTGGGCAGGATGAAATATCAAAAATGGTGCAGGAATTAGAAAAAGTGGGCATTATAAGAACTGCACACAGCCCATATAATTCCCCCATGTGACCAGTGCAAAAGTCAGATGGCACGTGGAGAATGACAGTGGATTACAGGGAATTAAAAAAGGTCACGACACCTATTCATGCAGCCGTACCCAATATTGCCTCCCCAATGGACACATTGAGTAGAGACATAAAAACCTACCATTGTATCCTAGATTTAGCAAATGCATTCTTCAGTATTCCAATTGCTGAGGAATCGCAAGATGAGTTTGCGCTTATGTGGGGAGGCAGGCAGTGGACCTTTCAGGTCCTGCTACAGGGGTACATGCATTCACCAACATATTGTCACAATGTAGTGGCGCGTGACCTGGCTGATTGGAAAAAACCTGATGATGTTAACCTGTATCATTCTATTGATGATTTCCTGCTGACATCCAACTCACTGGAGGCAGTAGGGCAGGTGGCAGATTCATTAACCACCTATTTACAATTAAGGAGATGGGCTATAAATCCCCAAAAGGTGCAAGGTCCAGGCCTGTCCGTAAAATTCCTGGGGTGGTTTGGTCAGGAAAGACCAAAGTACTACCCAGTGCTGTCACAGATAAGGTTCAGGCGTTCCCAGTCCCTACAACATCGAAGCAGCTGCAAGAGTTTCTAGGTATATTGGGTTACTGGCTTTCCTTTATACTTCACCTAGCGCAGCCTCTAAGGCCACTGTACAGACTCACAAAAAAGGGGCAGCTATGGGATTGGGGGAAAACAGAACAGGTCGCTTTCCAACAGGCAAAACCGGCAGTTAAACAAGCCCAGGCATTGGGTATATGCCCCAGTATAAGTGGCAGTGTTGACCCAGGATAGAAATACGGCCTGTATCTTGCCCTGGAGGCAGACCTTCCCCTCCTAGTACCCCTGAAACATCTGTGGACCCCTTGAGTCTTTGAGCCTTCAGGATCACCATAAGGAACGAAATGCCATCAGAGTGTTCCAGTGTTACTGGCAGATCACGTGTAACACCCGGTGATGGTCCACATGGGACTGATGGAGCACAGGATAGACCTGCACCTCACGACCGCATGCCTCCAGTAACATCATCGAGCACTGGCCCATAAAAGAATATGGACGTTCACCGATTGTAACTCATCTTGAATTGTACCAACATGCGTTGCAATATAGCAGATCTCTGTATTAGGAAAAAGCTTACCCTGTAGTCAACCTGATCATTGGTTCATGCCATGAAGGGGTGAAGTGTATAGGAACTGCTGAATCATGGCCTGAACCTCTGATGGATCACCTGAGGCAAGCAATGAGTCAGCCCTGGGAGCACAGGTGAAGGCAATTCCCCTGTGCTGCCAGAAGGTGTGGAGCTTGGCTCCACCTCTTCTAGACCCATGTAAGAGCTGACTACCAGTGGGGAAGGATCTCTTCTGGAGATCCCTCCTCTGGAGTTTTGCAGTGAGCCCAGGACATGGGTAAGCTTTCCTTCTATTCTTTTTGTTATTATAATGTGCTCTGCCAACCACTCTTGTTTATTTCACAATTATAACATCTTTATATTCACTGACTATACATGTGGATTATGGAGTTTGATTCTGCACATCAAAACCCTCTGCCCGTAACT

>Birddawg_Ib#LTR input_4675_PT_B4_L6_2_fsta.rev

GTAAGCATTCTATCTTTTCTGCTTTGGTGTAATAACTATGTAGCTAAGCTCTTTGGTATACTGTAACATCTGTTTATTCATTGATTGTACACTTGGTGAGCCAGGCAGGCCAAATATGCTTTATACTATGTCCTTTCTGTGGAATTTCTATAAATGGGTTGGGGGCAGAACATCACCCCTTTAAATAAAATGATCCCTGGACAAATCCCAGGTTTTCTGGGTTCTCCTTATTACTAATTAGCAACTGTAGTTGAGAAATGGGGTTCCCTGCATACTATGGGGAATGTTTCCTCATTCCACATGATGGAATACTTCCAAGAATTGGACGGAGACATTCTAACTACAAAAGAAAGGCGGTTAGCTGCCTCCACGGTGGCATGGTCACCTTTAAATGCTTTAAATCAAGCTGAGATAAGAGCTGACACCTTAGAAAGTGAAAATCAGATATTAAAAGATTGTACTGAAAAACTAGAATGGGAACTTAATGTATTAACAGGGAAAAGACCAACTTTAGATTCCCTGATAGGTCAGGTCTGCAATATTTCAATAGATAATAACTACACCTCTGGAGTAATGGACCTGGTTGAACAGGAAAACAAAAACTCTAGATTTAGAGGTTCCACTTGTGGCGGATTCCTTGGAACTCCATCCTATTATAACCCAAAAGACAAAGCAAGTACAAGATCAACCAGCAGAGGTGCCCCCTGACCAATGGCCCCTGCCCAAATGCATTTACTCTTAATGGCTAGGCCATATACACCAACAGAACTAATGAATATGGTGCAGTGATTTCGGCAGAGGCTGAAAAAGTGTACCCATGTGGTTACTTAAATTATGGGATTCAGAGGTGGAAAGCATCATGGTTAATGGGCCAGAAGTATCAAAATTGGCCACCATGATGGTACACCCTGCCCTCAGACAGAGACTGTATGCTGCTGTTCAGTGTAACAATGAGAACCATTCAATAATAGACTGGTTAATGGCAACATGTTGCATGGTTTGGCCAAACAAATCAGATATTCCATTAAACACAGGATTATGGTCCTCCATGGAGGACCTTCAAAATTATATTTGTGAACTGGGCGTAAGAGAAGCGATATATGAGGACTAGTTTAAAAGCCCAGATATGGTAAAGTTCTCGGCAGGTATGCAAGATTTAATCTTACAGCAGGCCCCTTCTCACCTATATGTGACATTAGTTTCCATATTAAAGCCCCTGTTGGCCTCGAAGGCTGTGATTCAATAGGCTGCCCAGATAGTAGCTGATTTGGGGGAGATAGAGCATTTGTGCACTAGGCGTGATATTTGAGCAGTTGAAGAAGCAGAGGTGCTTCCAGTAGTTAAAACCGAGAGGCCACCTGTGCAGAATCCTCAGTCAGGACTTGTAAGGGTCTTGCAAAAACAAATGTTTAATGATCTCATTCAGGCTGGGGTCCAATTTGCAAAAATTGACTGCCAGCTTAGTCGCATCCTCCTTCAGCTGTGGATTGAAGGACAATCAGAGATTCCAGAATTACCCTAAGAAACCAAGGGTAAGAGATACAGAAAGTGTACCAATGATGATGAGGATTTCATTGTTCCCAATAGAAAAAAGAAGCCCCCTCAGGTGTCTCAGGCTACAGTGCCTGAGCCACCTGAAACTAAAGACTTGACTTTGGCTCAGTTCATGGAGTTGAAAATATTCTGGTCCTGGAAAAATGTCCAGAGTTATGGCACTTGTTGATACTGGAGCAGTAACATCAATTATCTACAGAGATCTGACCAAATTTGATGGAGACAGAGTGGTGATTGGTGGTTCTGGGGGACAGACTATTCCTGTCACCAAAACATGGTCGAAACTGAGGGTTAGGCATCTCCCACCCCGGGAGTATAAGGTATCTATTGTCCAAGTCCAGGAATACATCTTGGGCATAGATATTCTATGGGGTCTGGCTCTCCAGACAACTGTGGGAGAGTTCAGATTTTGACAGAGATGTATTAGTATCCAGGCGGTGCAGGCAATATTAAGAGACCATGTGAAACATGGGCCTATTTGCCTGCCGAAACTGTGCCGGATTACTAACGTAAGACAGTTAGACTTCCAGGTGGGCAGGATGAAATATCAAAAACGGTGCAGGAATTAGAAAAAGTGGGCATTATAAGACCTGCATACAGCCCATATAATTCCCCCATATGGCCTGTGCGGAAGTTGGATGGCACGTGGAGAATGATGGTGGATTATATAGAATTAAATAAGGTCATGCCGCCTATTCATGAGGCTGTACCCAATATTGCCTCCCTAATGGACACACTGAGTAGGGAGATTAAAAACCTATCATTGTGTCCTAGATTTAACAAATGTGTTCTTCAGTATCCCAATTGCTGAGGAATCACAAGATCAGTTTACGTGGGGAGGCAGGCAGTGGACCTTTCAGCTCCTGCCACAGGGGTATGTGCATTCACCAACGTATTGTCACAATCTAGTGGCGCGTGACCTGGCTGATTGGAAAAAAACTGATAATGTTAACCTGTATCACTATATTGATGATCTGCTGACATCTGACTCAATGGAGGCAGTAGGACAGGCGACGGATTCATTAACCGCTTATTTACAACAGAGGGGATGGGCTATAAATCCCCAAAAGGTGCAAGATCCAGGCCTGTTTGTAAAATTCCTGGGGGTGGTTTGGTCAGGAAAGACCAAAGTACTACACAGTGCTGTTGTAGATAAGGTTCAGGTGTTCCCAGTCCCTGCAACATTGAAGCAGCTGCAAGAGTTTCTAGGTGTATTGGGTTACTGGCATTCCTTTATACCTCACCTAGCGCAGCTGCTAAGGCCACTGTACAGGCTCAGAAAAAAGGGACAGCTATGGGATTGGGGGAAAACAGAACAAGATGCTTTCCAACAGGCAAAACTGGCAGTTAAACAAGCCCAGGCATTGGGTATATTTGATCCTATCCTCCCAACCGAGTTGGACGTTCACGTCACTCAAGATGGCTTTGGTTAGGGCCTGTGGCAACACCAGAGTTCTATTTGGACCCCCCCATTGGTTTCTGGTCTCAGGTCTGGCATGGAGCAGATGAAAGATACAGTATGATTGAAAAACAGTTATTGGCTGCCTACTCTGCATTACAGGCAGTAGAGCCTCAAACAGCTGAAGTTTTAGTTAAAACCACTCTGCCGATTCAGGGGTGGGTGAAAGGTCTGACCCACCTTCCTAAGACAGGGGTGGCCCAAGCACAAACAATGGCATGATGGGTTGCCTATCTCAGCCAAAGGAGCAGTCTGTCTTCATCACCCTTGAACGAAGAACTTCAGAAGATCCTAGACCTGGTGACATATCACAGTAATGCACCAAAAGAAACACTGGTCTCTCCACCAGAGAAGAGTCACATTCAGGAGGGGAAAATATCCTATCCCTGAGGATGCCTGGTACACAGATGGGTTCAGCAAGGGCAGCCCAAGCAAGTGGAGAGCAATAGCATACCATCCTTCCCTGAGACAATCTGGTTTGATGAGGGGAATGGTCAGAGCAGCCAATGGGCAGAACTGCGAGCTGTGTGGATGGTTATAACCAAGGAACTTGGTGATGGTATCTTGAGTATCTGTACAGATAGTTGGGCTGTGTACCGGGGGCTCACTCTTCAGATTGCACAGTGGGCCACCCAGGAATGGACTACCTATGCCTGGCTGATCTGGGACAAGGACATGTGGTTAGACATATGGAATACAGTCAAACACAGGACTGTATGTGTCTACTGTGTTTCTGGCCATCAGCCCCTACAGTCACTGGGATGAAGCCAACATGCTAGCCTGAGTTCGATGGATTGAGAATACACCATCTGAGAACATCACCCGTTGGTTCCATCAGAAGCTATAGCATGCAGGACAAAAGACCATATGGGCAACTGCTAAAGCATGGGGACTGCCTGTACAGCTACCTGATATCGTCCAGGCATGCTGGGATTGCAACACTTGCTCTGAGACCAAGATCATTGCCTGAAACAACAGCCCATCTTGCTAGAGGACACAATCGTCTCCAGCGATGGCAGGTCGATTACATTGGGTCCCTTCCTTGGTCTGAGGGGGTGAGATATGCCTTGACCTGCATCGACACTGCAAGTGGGCTACTGCAGGCCTATCCAGTGCCAAAAGCAAACCAGGCATATACCATCAAGGCACTCACTAAACTGATCTCTGCCTATGGGACACCTCAAGTCATCAAGAGCGACCAAGGGACTCATTTTACTGGTGCAATGATACAATGCTAGGCAGAAGAAAATAACATTGAATGGCGATTCCACCTGCCATATAATCCAATGGGGGCAGGCCTCATTGAATGTTATAATGGTATTCTTAAGGCTGCCCTGAAGACAGACTCCCAGTCCCTGCAGGGGTGGATGAAAAGACTCTGTGAAACCTTGCGGGACCTGAATGAAAGACCCCGAGATGGCAGACCCAGGGCCCTGAGAATGTTGCAGGCAACATGTGCTACCCTGCTTAGGATCCAAATTACAGGCACTGATAATCAGATAAGACACCAGATTGGTAATGAAAATGATCTTCTGCTCCCTGCCCCTGAGAATTTAGATCCAGACACCCATAGAATAAAATGGCCTTGGAAGGTGCAGGTAGGACCTGCAGGTGGTGTGGCCTACTTGCACCTTGGGGGAGATTATTGGAGGTGGGAGGCTCAATAGTCACTCCAGTAATAGGTACATGGCCTACTGATATTGTGGTCAACACTCCAATCTTCATTGCTAAAGGGGCTCCCATCCTGTCCCTATGACAGATCAGGACACCCCCTTTGGTGCCTGATATTATTATGCAGCTGCAGACATCTGGCTAGAAGGTGTGGTACAGGCGGCCAGGATGTGCCCCTATACAAGCCAAAGTGTTGACCCAGGATAGAAACACAGTCTCTATCTTGCCCTGGAGAGCAGACCTTCCCCTCCTAGTACCTCTGTATTACTCCCTGTAAGTCTTCGAGCCTTCAGGCTCACCAGAACGAATGAAATATAATCAAAGCGTTTCAGTGTTACTGTCAGATCATGGGCAACACCTGCTGATGGTGTACATGGGACTGATGGAACATAGGATGGACCTGCAACTCACGACCACATGCCTCCAGTAACATCGAAGAATACGAATGTTCACTGACCGTCACTCATCTAGAATTGTACCAACATGTGTCACGATGGAAATTGTATAAGCATTGCGATATAGCAAATCTCTGTATTAGGAAAAGCTTACCCTTTGGACAACCTGATCACTGGTTCACTCTGTGGAGGGTGGAGTGTGTGAGAACTGCTGAATCACAGTCTGAACCTCTAATTGATCGCCTGAGGAGAGCAATGAGTCAGCCACAGGAGCACAGGTGAAGGCAATTCACCTGTGCTGCTGGAAGGGGTGGAGCCTGG

CTGCACCTCTCCTAGACCCATTTAAGAGCTGACTGCCAGTGGGGAAGGATCTCTTCTGGAGATCCACTCTGCTGGAGTTTCATAAGTGAGAGTAAGTGGGTAAGCATCCTATCTTTTCTGCTTTAGTGTAATAACTATGTAGCCAGGCTCTTTGGTATACTATAGCATATGTATATTCATTGATTGTACATAGGTGCTTGTAAAACATCTCAAATTATGATCATTTTTCCACCAGTTACT

>Short1#LTR R=92_rev

TGAGGAGGCACAAATTATTTGGGGCCTGGCTAATGCTTATCGGGCCTTATTCAATACTATCCTGGAGAGGGAGAGAGTTTGTGAAATTGAAAAGGAGAGTCTCAGGGAAACAGTGCAAAATGAGCGAGAGAGTCTCCTGGTCGAGAGAAACAGCCTCCAATCTGAGAGAGATGCCCTCCGAATTGAGAGAGACTCCCTGCAATCCAAACTCAACAGTCTTCAATCTCAACAAGACACCCTCCAATCTGAGCTAGATACTCTCATAATCGAACGAGACAGGCTCCAAGCTGAACTGGAGGATGAGAGCATTGGAACTGACCAACCTGATCAACTACAGGAAGCACCACAATTGATGTCAGTTGCCCCTGTAAGAGGACGGAAAATGAAACGAATCTCGACTCAGTTAGAAGAGAAGAAAGAGGAGGAAAGAGGTCCAGAGGAGTGGCTCCAAGATCCACAAGAAGCACCCCTGAGTCCAGGGGAGGGGCCCTCAGTAAGACCACGGTCACCAACGCCTATGAGGGCAACAAGAAGTCCTGAAAGAGTAAGAGGTGAGCAAGATATCGTTACCATTGTTGATCGATCTCTGAAAATGAATGAAATTCGAAGTCTGAGAAAAGACTTTTCACGTCACCCAAATGAGCCTATTGTCACCTGGTTACTTCGATGTTGGGACAATGGGGCCAACAGTGTGTGGCTAGATAGTAGAGAAGCTCGCCAACTGGGTGGCATTGCTAGGGACTCAGCCATTGACAGAGGTATTAGTACATGCCAGAACCAGGCCTTCACCCTCTGGAAGCGGATGTTGTTAGCTGTAAAAGAAAGATACCCCTTCAAAGACGATCTGATGCCTGAGAAAAAGAAATGGACTGATATGGAAAAAGGCATCTGTTATTTGAGAGAATATGCTGTGGTGGAAATGCTACATAGCCCCGATTTCATTCCTAATGAGCCAGACCAAGAGCATGATCCTGAGAGAGTCAGGTGTACACCAAACATGTGGCGTACATTCACAAAGACTGCACCAGAAAGGTACGCCAGTACATTTGCAGCAATGTATGGCAGAGGGGAAAGAAGACCCCTTATAAATGAATTGGTTAATAAACTTCAAGACTTTGAGTTACATTTAAGCCCTCTACGAGCTTGTGTTTCAGCCATTACAAAAGTAGCTGAAAAGCTGGACAGAATGGAGAACAAACAAGAAGATATAATAGACAAACTATCAACCGTGCCTGATGCTGATGAATCCATGGTGGTAACAGATACATCAAATGGAGACCAGAATTCCCAAAACAGCCTATTGGAGGGGCTGATCAACTTGATTTCCTCCCAGCCGGTGGCATCCAATGTCTCAGCTATCAAAAGAAGACGTTCTCCTGCTCGAGCAAGTGACAACAGTAAGACCACATCACGTTTTGCCTTGTGGCATTACCTACGTGACCATGGAGAAGATATGAAGGAGTGGCATAAACAACCTACTCCTGTACTTCAAGCACGGGTAAGAGAATTACAAGACAAATCAACCACCAAGGTGAACTTCTCCAAAAAGGTGAT

>Kronos_Id#LTR R=176_rev

GCGGAGGAGGAGGAGGAGGAAGATCCAGGCGAGGGGCCCTCCTCAGAACCACCACCATCGGGAAAAGCAAAAGGAAAAACTAAGAGACATGCAGAAGAGAGTGATGATGAGGACGTCTTTATTACTACTCGTCGGCCTCTGAAGATGACTGAAATCCAAGGCTCAAGAAAGGAGTTCACACGGGGCCCGAATGAAACTCTCGTTTCCTGGTTGCTTCGCTGCTGGGACCGCGGGGCCAATAGCTTGTTTCTAGATGGTAATGAAGCTCGCCAACTAGGAGCCATTGCCAGAGATCCAGCTATTGACAGAGGAATTAGTCGATGTTTGGATGAGGCTGCCACCCTCTGGGAACGAGTGTTAATAGCCGTGAAGGAAAGGTATCCCTTCAAAGACGGCTTGAAGCCTGTGATGAAAAGATGGGATACAGTTGAAAAGGGTATCCAGTATTTGAGGGAAATGGCTGTGGTGGAAATGTTGTTTGACCCTAACTTTGTTCCTAACCACCCACGACAAGACCACGACCCTGAGAGAGTGAGGACGACGCCTGAGATATGGCAGAGACTCATAACAACAGCACCAGACAGATATGCCGGTACACTACTGTCAGCTAATGACAGATACCAGGAACAAGAGAGAATACCCTTAGTTTTTGAATTGATTCTTACGCTCCAAAACTACGAACAACAGCTACCCCCAACTCACGTTTCCATTGCCGCCATCTCACAAATAACGGACCAACTGAATGAAATGCAGGAGCAAATGTCTCTACTGATTAATCGTGACAAACCTGTGCCAGTATCAGAAATGCTCGATGAAGATCAGGATAATCAAAAGAGTCTAATGAAGGAGCTGATCAAACTAATGCGAATCCAACTAATTAACGATGATGAACCTTCCTGCTCACGGGCACCATCAAAAATCTCAGCTATTAAAGGCAAACGCTTTCCGGCTCCAGTGAGGAACAATACATCGCGAATTGCCTTGTGGCGTTACCTACGTGACCATGGAGAAGACATGAGA

AAATGGCACGACCAAGCTACTCCTGTACTACGAGCACGGGTGAAAGAATTACAAAGCAGATCAACCACCAGTGCAGTTGCTCCAGTTATCACAGGTAATGAATAGAGGGGCCCTGCCCTCAGTCGGGGGGGGAAAAGGATAATCGAGTATATTGGACTGTGTGGATTCGATGGCCTGGCACGTCAGAACCACTGAAATATAAGGCACTGGTGGATACTGGTGCACAGTGCACTCTGATGCCCTCGAGTCATGAAGGGACAGAATCAATCCATATTTCCGGGGTGACTGGAGGCTCTCAAGAATTGACTGTGTTGGAGGCCGAGATAAGCCTCACTGGTAAGGACTGGCAAAAACATCCTATTGTAACTGGCCCAGGGGCTCCATGTATACTAGGTATCGATTACCTCAGAAGGGGGCATT

>Kronos_Ib#LTR R=84_rev

GGAATCTCAACCGTCCAGGAATCCTAGAGGTGATCATGGACTGGCCTGAAGGTAAAAAGTTTGGAACATCACCAGCAGAAGAGGTATCACGTGCTAAAGAGGCCCCACCATATAATGAACTACCAGAAAATGAAAAGAAATATGCCCTGTTCACAGATGGATCGTGTTGTATTGTGGGGAAGCATCGCAGATGGAAAGCCGCTGTGTGGAGCCCCACACGACAAGTTGCAGAGGCCACTGAAGGAAAAGGAGAATCGAGCCAATTTGCAGAGGTAAAGGCTGTCCAACTGGCCTTGGATGTTGCTGAACGGGAGAGGTGGCCAATGCTTTATCTTTACACTGACTCATGGATGGTGGCAAATGCTTTATGGGGGTGGTTACAGCAGTGGGAGCAAAATAACTGGCAAAGAAAGGGTAAACCTATTTGGGCTGCTGAGCTGTGGAAAGATATTGCTGCCCGAATAAAGAATATGGTTGTAAAGGTGCGCCATGTAGATGCTCATGTGCCCAAGAGTCGGGCTACTGAAGAACAGCAAAATAACCATCAGGTAGACCGAGCTGCCAAAATTGAGGTGGCTCAAATAGACTTGGACTGGCAGAACAAGGGTGAATTATTTCTAGCTCGATGGGCCCATGAGACCTCGGGCCATCAAGGAAGAGATGCAACATATAAGTGGGCTAGAGACCGAGGGGTGGACTTAACTATGGATGCTATTGCACAGGTTATTCATGACTGTGAAACATGTGCCACAATTAAACAAGCCAAGAGGATGAAACCTCTCTGGGGGGAAGGGCGATGGCAAAAGTATAAATATGGGGAGGCATGGCAGGTTGATTATATCACCTTGCCACGATCTCGCAATGGTAAGTGTTATGTGCTTACCATGGTGGAGGCAACCACTGGGTGGCTTGAAACATATGCAGTACCCCATGCTACCGCCCGAAATACCATATTAGGTCTTGAGAAACAAGTCCTGTGGCGACATGGCACCCCAGAAAGAATTGAGTCAGATAATGGGACTCATTTTAAAAATTCTCTTGTAAATACTTGGGCCAAAGATCATGGCATTGAGTGGATTTACCATATTCCCTACCATGCACCAGCCTCTGGTAAAATTGAACGATACAATGGGTTGTTAAAAACTATGCTGAAAGCAATGGGAGGCGGTACATTTAAGCACTGGGAGAAGCATTTGGCAGAAGCCACCTGGTTAGTCAATACCAGAGGATCTATCAATCGTGATGGTCCTACCCAATCCAGCTCCCTACATACCGTAGAGGGAGATAAAGTCCCTGTAGTACATGTAAAGAACATGTTGGGAAAGGCAGTTTGGGTTTTTCCAGCTTCTGGGAAGGGCAAACCTCTTCGTGGAACTGTTTTTGCCCAGGGACCTGGGTCCACTTGGTGGGTGATGCAGAAAAACGGGGATGTTCAATGTGTACCACAAGGGAATTTGATGTTGGGGGAGTGCAGTCAGTAATTCCATGTGTGTGTATTTATATTTTTTTTTTATATATTAAGCATGATGTATTGATTTGTAATAAGGGATGTATTGTCATGGTTTAATGATTTTGAGTTATCGGTATTCCACATCATAACATCATGTAATGCACTGGGGGTTTGACTGCTGATGCTCAAGTTCCGGGTGCCTGTCCGGAGGAGAAGAAGAGCTACATTTCCCCAGGGGGCTTTGCGGTCAGCGAGGAGATATAACTCCCGGCAAGGTCACCTGATGCTCTCTTCTTTGCCTGCGCTTCTTCGCTTGCGCTTCT

>Short3#LTR R=325

AGTCATTGATTTAAAAGACTGTTTTTTTACCATTCAACTTCATCCAAAAGACGCACCACGATTCACTTTCTCAGTGCCCTCCATCAACCAACAAAGCCCTGCTCAGCGGTATCATTGGACTGTGTTACCACAAGGAATGCTTAATAGTCCAACAATATGTCAATTGACTGTAGCAAATGCATTACAACCAGTACGAAAAGCTAGACCTCACATCCTTATTTATCATTACATGGACGATATATTAATAGCAGCAGAACAAGAAGAGGGTCTAAAGGACGCTTTACTGCTCATCTGGCAGGCAGTCCAATCTGTCAGTTTACAAATAGCTGAAGAAAAGGTACAACTCAGTTCTCCATGGAAATATTTAGGATGGAAAATTACATCTCACACCATTCAGCTACAAATCCTCAAAATAGTACCTCACGTACAGACATTAAATGATCTGCAAAA

>Short2#LTR R=597_rev

GAATGGCCAAGTGACTGGCCATTGATTCCATCTTGTGCGGTGGTGGGTGTGGGAGGTTCTCTTCTAGCAAAGAGGGCAAGGGATGAAGTCGAAATCATGTCAATAAATAGGGACGGGACCGTTGAAAGACCTGTCCTGATAACTCCCTTTGTAGCCCAGGTGCCGGGTACGTTATTAGGGAGGGACTTCTTGAGGGGGCTTGGGGCACGCATTACAAATTTATGATTAGGGCCACTGTCTGCCAATTGTCTGCCACTCATGCCATTCCATTACGCTGGAGATGGGATGTCCGCCCCGTCTGGGTAGATCAGTGGTCCCTTTCACGTGATAAGCTTGCGGCCCTTAAATATTTGCTTGCTCAGGAGCTTCGCCTTGGGCATCTTGAACCCTCACTTAGTCCGTGGAACACCCCTGTGTTCGTCATTAAGAAACGGTCAGGGGCCTATCGCCTGCTACATGACCTACGTGCGGTTAATGCTCAATTGGTCCCCTTTGGAGCCGTGCAACAAGGTGGCCCTCTTTTATCAGCCATTCCCAGAGGATGGTCGTTAGTAGTAATTGATCTTAAGGATTGCTTCTTTTCTATTCCTCTGGCAGAGGAAGACCGGGAAGCGTTTGCTTTCACTGTTCCAACCGAGAATAATGCTGGGCCTGCCGAAAGGTATCAATGGCGGGTCCTGCCTCAAGGAATGGCA

>Kronos_Ic#LTR R=100_rev

TTGCCACTTGGTTACTTCGATGTTGGGACAATGGGGCCAACAGTGTGTGGCTAGATAGTAGAGAAGCTCGCCAACTGGGTGGCATTGCTAGGGACTCAGCCATTGACAGAGGTATTAGTACATGCCAGAACCAGGCCTTTACCCTCTGGAAGCGGATGTTGTTAGCTGTAAAAGAAAGATACCCCTTCAAAGACGATCTGATGCCTGAGAAAAAGAAATGGGCTGATATGGAAGAAGGCATCTGTTATTTAAGAGAATATGCTGTGGTGGAAATGCTACATAGCCCCAATTTCATTCCTAACGAGCCAGACCAAGAGCATGATCCTGAGAGAGTCAGGTGTACACCAAATATGTGGTGTACATTCACAAAGACTGCACCAGAAAGGTACGCCAGTACATTTGCAGCAATGTATGGCAGAGGGGAAAGAAGACCCCTTATAAATGAATTGGTTAATAAACTTCAAGACTTTGAGTTACATTTAAGCCCTCTACGAGCTTGTGTTTCAGCCATTACAAAAGTAGCTGAAAAGCTGGACAGAATGGAGAACAAACAAGAAGATATAATAGACAAACTATCAACCGTGCCTGATGCTGATGAATCCATGGTGGTAACAGATACATCAAATGGGGACCAGAATTCCCAAAACAGCCTACTGGAGGGGCTGATCAACTTGATTTCCTCCCAGCCGGTGGCATCCAATGTCTCAGCTATCAAAAGAAGACGTTCTCCTGCTCGAGCAAGTGACAACAGTAAGACTACATCACGTTTTGCCTTGTGGCATTACCTACGTGACCATGGAGAAGATATGAAGGAGTGGCATAAACAACCTACTCCTGCACTTCAAGCAAGGGTAAAAGAATTACAAGACAGATCAACCATCAAGGTATACTTCACCAGAAAGATGATTACTGCAGTTGCTCCAGACAATGCAGGTAACAAATAGAGGGGCCCTGCCCCCAGCCAGGGGGGGGAAAGGGATAATAGAGTTTATTGGACTGTGTGGATTAGATGGCCTGGCACATCAGAACCACAGAAATATAAGGCCCTGGTGGATACTGGTGCACAGTGCACTCTAATGCCCTCGAGTCACCAAGGGACAGAATCAATTTATATTCATGGGGTGACTGGGGGTTCCCAAGAGCTGACTATGTTGGAGGCCGAAATCAGCCTCACTGGTAAAGACTGGCAAAAGCACCCTATTGTGACTGGCCCAGGGGCTCCATGTATACTTGGTATTGATTACCTCAGAAGGGGATATTTTAAGGATCCCAAGGGGTATCGATGGGCCTTTGGAATAGCTGCTGTAGACACAGAAGGTGTTAAGCAGCTGTCTGTTTTGCCTGGCCTGTCAGAAGATCCATCTGTTGTGGGGTTGCTACGAGTGAAAGAGCAACAGGTACCGATTGCTACAAAAACGGTGCACAGACGGCAGTACCGCACCAACAGGGATTCCTTGCTCCCCATTCATAAGTTGATTCGTCAACTAGAGAGTCAGGGAGTGATCAGCAAAACTCACTCACCTTTTAACAGCCCCATATGGCCAGTGCGTAAAGCCAGTGGAGAATGGAGGCTGACGGTGGACTACCGTGGCCTGAATGAAGTCACACCCCCACTGAGTGCTGCTGTGCCAGATATGTTAGAACTCCAATATGAATTGGAGTCAAAAGCAGCCAAGTGGTATGCCACCACTGACATTGCTAATGCCTTTTTCTCCATTCCTTTGGCCACAGAATGTAGGCCACAGTTTGCTTTCACCTGGAGGGGCGTTCAGTATACCTGGAATCGTTTGCCCCAGGGGTGGAAACACAGCCCAACCATTTGCCATGGGTTGATCCAAGCTGCACTGGAACAGGGAGGTGCTCCTGAGCACTTACAGTACATTGATGACATTATTGTGTGGGGCAACACAGCAAAGGAAGTTTTTGAGAAAGGAGAGCAAATAATCCAGATCCTTCTGCGTGCTGGTTTTGCTATTAAGCGAAGCAAAGTGAAAGGACCTGCCCAGGAAATTCAGTTCCTAGGTATAAAGTGGCAAGATGGACGTCGTCACATCCCAACAGATGTGATCGACAAAATCACTGCCATGTCTCCACCCACTAATAAGAAAGAGACACAATCTTTTCTGGGTGTAGTGGGTTTTTGGAGAATGCATGTTCCAAACTACAGCCTCATTGTAAGCCCCCTTTATCAGGTGACGTGGAAGAAGAATGATTTTGTGTGGGGTCCTGAGCAGCAGCAGGCTTTTGAGCAGATTAAACAAG

>Kronos_Ia#LTR R=75_rev

TGATTACCTCAGAAGGGGATATTTTAAGGATCCCAAAGGGTATCGATGGGCCTTTGGAATAGCTGCTGTAGACACAGAAGGTGTTAAGCAGTTGTCTGTTTTGCCTGGCCTGTCAGAAGATCCATCTGTTGTGGGGTTGCTACGAGTGAAAGAGCAACAGGTACCGATTGCTACAAAAACGGTGCACAGACGGCAGTACCGCACCAACAGGGATTCCTTGCTCCCCATTCATAAGTTGATTCGTCAACTAGAGAATCAGGGAGTGATTAGCAAGACTCACTCACCTTTTAACAGCCCCATATGGCCAGTGCGTAAAGCCAGTGGAGAATGGAGGCTGACGGTGGACTACCGTGGCCTGAATGAAGTCACACCCCCATTGAGTGCTGCTGTGCCAGATATGTTAGAACTCCAATATGAATTGGAGTCAAAAGCAGCCAAATGGTACGCCACCACTGATATTGCTAATGCCTTTTTCTCCATTCCTTTGGCCAAAGAATCTAGGCCACAGTTTGCTTTCACCTGGAGGGGCATTCAGTATACCTGGAATCGTTTGCCCCAGGGGTGGAAACATAGCCCAACCATTTGCCATGGGTTGATCCAAGCTGCACTGGAACAGGGAGGTGCTCCTGAGCACTTACAGTATATTGATGACATCATTGTGTGGGGCAACACAGCAAAGGAAGTTTTTGAGAAAGGAGAGCAAATAATCCAGATCCTTCTGCATGCTGGTTTCGCTATTAAGCGAAGCAAAGTGAAAGGACCTGCCCAGGAAATTCAGTTCCTAGGTATAAAGTGGCAAGATGGCCGTCGTCACATCCCAACAGATGTGATCAACAAAATCACTGCCATGTCTCCACCCACTAATAAGAAAGAGACACAATCGTTTCTGGGTGTAGTGGGTTTTTGGAGAATGCATGTTCCAAACTACAGCCTCATTGTAAGCCCCCTTTATCAGGTGACACGGAAGAAGAATGATTTTATGTGGGGTCCTGAGCAGCAGCAGGCTTTTGAGCAGATTAAACAGGAGATAGCCCGTGCCGTGGCCCTGGGGCCAGTACGGATGGGACAGGATGTAAAGAACATCCTCTACACTGCTGCTGGAGAGAAAGGTCCCACTTGGAGTTTGTGGCAAAGAGCCTCAGGAGAGACCCGAGGCCGACCCCTGGGATTCTGGAGTCGGGCGTACAGAGGGTCTGAAGAGTGCTACACTCCAACTGAAAAGGAGATCTTAGCCGCGTATGAGGGGGTTCGGGCTGCTTCCGAAGTAATCGGTACTGAAACACAGCTCCTTCTGGCACCTCGACTGCCAGTGCTGAACTGGATGTTCAAGGGAAAGGTTCCCTCCACCCATCATGCTACTGATGCCACTTGGAGTAAGTGGATTGCGCTGATTACGCAGCGAGCGCGGATGGGGAACCTCAGCCGTCCAGGAATCCTAGAGGTGATCATGGACTGGCCTGAAGGTAAAAAGTTTGGAACATCACCAGCAGAAGAGGTATCACGTGCTAAAGAGGCCCCACCATACAATGAACTACCAGAAAATGAAAAGAAATATGCCCTGTTCACAGATGGATCGTGTTGTATTGTGGGGAAGCATCGCAGATGGAAAGCTGCTGTGTGGAGCCCCACACGACAAGTTGCAGAGGCCACTGAAGGGAAAGGAGAATCGAGCCAATTTGCAGAGGTAAAGGCTGTCCAACTGGCCTTAGATGTTGCTGAACGGGAGAGGTGGCCAATGCTTTATCTTTACACTGACTCATGGATGGTGGCAAATGCTCTATGGGGGTGGTTACAGCAGTGGGAGCAAAATAACTGGCAAAGAAGGGGTAAACCTATTTGGGCTGCTGAACTGTGGAAAGACATTGCTGCCCGAATAAAGAATATGGTTGTAAAGGTGCGCCATGTAGATGCTCATGTGCCCAAGAGTCGGGCTACTGAAGAACAGCAAAATAACCATCAGGTAGACCAAGCTGCCAAAATTGAGGTGGCTCAAATAGACTTGGACTGGCAGCACAAGAGTGAATTATTTCTAGCTCGGTGGGCCCATGAGACCTCAGGCCATCAAGGAAGAGATGCAACATACAAATGGGCTAGAGACCGAGGGGTGGACTTAACTATGGACGCTATTGCACAAGTCATTCATGACTGTGACACATGTGCCACAATTAAACAAGCCAAGAGGATGAAACCTCTCTGGGGGGAAGGGCGATGGCAAAAGTATAAATATGGGGAGGCATGGCAGGTTGATTATATCACCTTGCCA

>Birddawg_Id#LTR R=62 (inverted_half_and_half)

TTTATATCATGTCTTTCTTTGGAATTTCTATAAATGGGTCAAGGGGGAGAACATCACCCCCCTAGATAAAATGATCCCTGGGCAGATCCCAGGATTTCTGGGATCTCCTTATTATGAACTAGCAACTATAATTGAAAAATGGGGTCCCCTGCGTACTATGGGAAATATTTCCCCATTACGCATGACAGAATACTTTCAGGGACTGGACAAAGATATATTAACTACAAAAGAAAGGCAATTAGCTGCATCCACAGTGGCATGGCCACTATTAAATGCTTTAAATCAAGCTGAAATAAGAACAGATACTTTAGAAAATGAAAATCAGCTATTAAAAGCTCGTATTGAAAAATTAGAACAAGAACTTAATGTATTAACAGGGAAAAAAACAATTTTACAGTCTCTAGGTCAGGTCCGTAGTATTTCAATAGATGATAGTCATACCTCTGAAGTAGCGGACCTGGTTGAACAGGAAAACAGGAATTCTAAATCAGATCTAGAGGTTCCACTTATGGCGGATCCCCTGGAACTCCGTCCTATTATAACTCAAAAAACAAAAAAAGTACAAGATAGACCAGCAGGGGTGCCCCCTGACCAATGGCCCCCTGCCGAAACGCATTTGCATGTAACAGCCAGGCCATACACTCCAACTGAATTAATGGACATGGTGCAGCGATTTCGGCAGAGGCCGAGAGAGAGTATACCTGCATGGCTGCTCAGGTTATGGGATTCAGGGGCAGAAAGTGTTATGGTTAATGGGCCGGAAATGGCAAAGCTGGCCACCATAACATTACATCCTGCTGTCAGGCAGAGACTATACGCTGCTATCCAGTATACCAATGAGAACCACTCAATAATAGATTGGTTAATGGCAGCATGCCGGATGGTCTGGCCAAATAAAACAGATATTCCACTAAACACAGGACTATGGTCCTCCATGGAGGACCATAAACCTGTATTTAGGGGAATATCTGTTTTATTTGGCCAAACCATGCAGCATGCTGCCATTAACCAATCTATTATTGAGTGGTTCTCATTGGTATACTGGATAGCAGCATATAGTCTCTGCCTGACAGCAGGATGTAAAGTTATGGTGGCCAGCTTTGCCATTTCTGGCCCATTAACCATAACACTTTCTGCCCCTGAATCCCATAACCTGAGCAGCCATGCAGGTACACTCTCTCTCGGCCTCTGCCGAAATCGCTGCACCAAATCCATTAATTCAGTTGGAGTGTATGGCCTGGCTGTTACATGCAAATGCGTTTCGGCAGGGGGCCATTGGTCAGGGGGCACCCCTGCTGGTCTATCTTGTACTTTTTTTGTTTTTTGAGTTATAATAGGACGGAGTTCCAGGGGATCTGCCATAATTGGAACCTCTAGATCTAATTTAGAATTTTTGTTTTCCTGTTCAACCAGGTCTGTTAATTCAGAGGTATGATTATCATCTATTGAAATACTACAGACCTGACCTATAGGAAGTTGAAGAATTGGTTTCTTCCCTGTTAATATACCAAGTTCTTGCTCTAATTTTTCAATACAGTCTTTCAAAAGTTGGTTTTCATTCTCTAAAGTATTATTCCTCATTTCAGCATGATTTAAAGCATTTAATAGTGGCCATGCCACTGTGGAGGCAGCTAATTGTCTTTCTTTTGTGATCAGTATATCTTTGCTAAGTTCCTGAAAGTAATCTGCCATGCGATATGGGGAGATATTTCCCATAACACATAAGGGACCCCATTTTTCAATGATAACCACTAGTTCATAATAGGGAGATCCCACAAATCCTGGGATCTGCCCAGGAATCATTTTATCTAGGGGGGTAACGTTCTCCCCCTTAACCCATTTAAAGAAATTCCAAAGA

>Kronos_Ie#LTR R=153 (392 bp overlap with R=176)

TCTTTGAAGCGATACCTTTCCTTCACGGCTATTAACACTCGTTCCCAGAGGGTGGCAGACTCATCCAAACATCGACTAATTCCTCTGTCAATAGCTGGGTCTCTGGCAATGGCTCCTAGTTGGCGAGCTTCATTACCATCTAGAAACAAGCTATTGGCCCCACGGTCCCAACAACGAAGCAACCAGGAAACAAGAGTTTCATTCGGGCCCCGTGTGAACTCCTTTCTTGAGCCTTGGATTTCAGTCATCTTCAGAGGCCGACGAGTAGTAATAAAGACTTCCTCATCATCACTCTCTTCTGCATGTCTCTTAGTTTTTCCTTTTGCTTTTCGCGATGGTGGTGGTTCTGAGGAGGGCCCCTCGCCTGGATCTTCCTCCACCTCCTCCTCCGCTTCTTCTCTTTTCCGTTCTAGACGCGTGGACACTCGTTTCCATTTCTTGCCTTCCACAGGAGCAACTGATATTGTTACTGGTGTCTCCTGTAACTGATCAGATTTGACTTGGAGATTCTCCATTTTGGCTTGAACCTCAGCTCGGAAACTCTCTCTCTCCAGAATAGTATTATATAGGGCGCGGTAAGCACAAGCCAAACCCCAAATAAGTTGTACCTCCTTAGATTTATGTAAGCCGCGCTGTCCCTGACTCAAATACTGACTTAGTTTCTCAGGATCCCACAGGTGTTCAAGAGTAAAATCCCATGACATTGCGGGTCCCCATGCTTCTAAGATCTTTCCTAAACCTCTCCATATTCCCTGCCAGTCAGCATTCTCTGGTTTTGGAGGAGACTCCTTCCCTATATAATGAAATACAATCAAGGAAAGCAATAGCACGGACACCAACATCAGTATTAATTCGAGATTCAAAAACAGTCTGACAAACCGATAAGAAAGCCTGGAGACTGGTCTGAACATCGTGCTGTTCGTAAAATTAGCCATTCCATCTGGGATGTAAATGTCAAAATTCAAACCATACCACATTGCATACAGGTACCATGCAGGTGTCTCCGTCAATGCCCTTAGGTAGTTATATATGAACACAATTCCACAACAAGTAATTATGGCATTGATAATTTGCCAATAAGTTGAGAATAACATGACAGCTTGTAAGCCTTTACTCAACACCCTCACGATGAAAAGTGGGTTCATTGCTGGTGTCTATTAAAAAGGGGTTAATGAATTGAAACTGACAAGAGGAAGGAGGAAAAAAGAAAAAAAAAAAAAAAAAGGCACTACCCAGACTAAGCAGTGCTCAAAGTTTACAAATATCCCAGAACACTGGCAGCACGCCTAGTCTTTCAAGGTCAAGTTTTCCAGCACAGAAACCTAAACTACTGATAATGCTCCCTAACGAAGCTCTCTAAGAGCAAAGAGAGAGATTCGTTCTAAAAGCTAAAAAGCAGATTTGCCCGCATTCTCCACCAAAAAATCTGTCACAGAGTTAACAAGATCAATTTACGCC

>Soprano_Ia#LTR R=606

CTTTGTTTCCTGACTGCAGATGGTTAGGGGTTACAGTTCCTTCTAACACTGCCCAGGTTCTTTCTCCCTGAGACATAGCCTCTATTTTTCCTTGCAGTTGTATACCTATAGGTTTTAACGACTCTGGAAGCCCCCTAATTAAAGGAGTCATCCTTTCAGGATCAACAGGCATCATCATAGGTGATTCATGGTGTGGCTGCAGCTTTCTATCATACATCATTTGGAGACAAGCAGCCTTTTGAACACTCTCTACTAACTGATCAGTTGTTCCAGTAATGGCAAGAGGGTCTCCCCTTTCTAAAGGGTTGAGACCACCTGCCCAGTAGGCAGCCCTCTGTGTTAAGGA

>Soprano_Ib#LTR R=889

GAGAGCGGCTAAAATCTTTTTTCAATTTTGCTAGTTCAGTGGCTGAGAAGGGAATTTCCTTAACATTCATTTGAGGATCTAGATCCTCGCTGTTATCGAAGGTATATTCGGTTTTTATCAAAGGACGCAATGGGGCTATTGGAGTTTCTAATTTGTCTAGTCTTTCCTTTATTTCTTGCAATCCCTCGTGAGGAAAAGTTTTTTCTTGAGGCAAACTATGGGTGACAGTATCGCTAGTATCCTTACATGTTAACAGTTCCTTAAAAGCCATCTGCAACAAAGCCGAATTACGTTTCTCTCTTTCTAATTGTTCCTCAAGGTTCACTATCTGATTCTGCAGAGTTTTTACCAGTTCTTGTGTTACTTTAACCGATTCCTGCAGTGCTTGTATAGTTTGGGTTTCTGCCGAATGCTTTTCATCTCGGAA

>Soprano_Ic#LTR R=675

TGAGAGTGTTGTCCTTCCAGGTATAGTCTGAAACTTTATTTGTGCGTACCTCCTGGAGTTGCAAGGAGCGATATTTTTGAAAACTTAGACGTGAGTCCTTCTCCAGGAAGACCGCTCCATATTCTGAAACTTTACTGTTTATGTGTATACCTCTCGGGGACGTATGAATTAATTTTGAATACGTGTGTGCCTCCTCAGGAAGACCTCTCTGCATTTTGTGATTTAAGTATCTTGTAACTTAAGTGTGAAATTTGAACCTTTTTGTGTGCTTCCCTCAGAGAAAGGGAAGAGTGATTGGAGCATTTGCATTTGTT

>R=20LTR#LTR

AAAAAAAAAATAAAAATAAATATATATATATATATATATATATGTATATAACAAGTGATGCACAAGCAATTGCTCACCACCCCCTGACCGATGCCCAGCCAGCCCCCGAGCAGCAGAAGAGAGAGAGATGAACTCCCACCCCCTTCAAAACTCCTTCCACATGATGTCATATGGTATGGAATATCCCTTTGGCCAGTTTAAGTCAGCTGTCCTGATTCTGTCCCCTCCCAGCTCCTTGTGCCCTTCACTGCCAATGGCCTTGGCAGTGTACAACACTGATTAGCAGCAACTATAAACATCAGTGTGTTATCAACATTGTTTTTCTCCTAGAACCAAAACATAGCATCATACCAGACACTCTGAAGAAAACAATTCCATCCCAGCTGAAACTAAGACA

>R=25LTR#LTR

TGTAAGAGATTATTCTTTTACATCATTGTCTCAAAGCTTGCTGGGGACACAGATGGACAAGTCCAAGCAAGTCCTGGGCAAGGGTTGAGAAATCCTTTGTCTGTGGAACACAGATGGACAAGGCCAGGGGCAAGGAGAGAGAGATAAGCTGCAGCTTAATGGCCGTGAAGCAGTCTTTTGTGTGGGCTTATCTCAAGGAAGATGGCCATCTCAGGAGGTGCTGCACATGACTCTTACCCAAGCACCCAGGAATGCCACGTAGGCAGAAGAAGAAGGAGAATAAAAGGAGGAACAAAAATCAGGAGGAGGTGTTTTTGCCATCAGATTCCTCATGAAGAAGAAGATGCGTGTTGCCGGACTCGCCTCGCCATGCTCCTCTCTCCCTCCTGCTTCTGCTCTGATCTCCTCCGCGACTTCCTTCTGCCACCTGCTTGCTGCCCAAGGCCGGCCACGCTGCTTCTCTCTCCCCCCCCCCCTCCTTCTACCTCGGACCATCGAAACGTCGTGCAACGGGACCGCTGCTGGATCCTGGTGGTGACTATCCCCGCTTTACGCAATTCTTGCTTCTTTCCTCTCTTTTCTATCGCCCTCCTTCCCTTCCCCGTCTCCCTGATTAGGATTATAATAAACTGGTTGGACCAACATTTGAACCGTTGTTTCTTAATCTCACGCCGGGTATATAATATTAAAAGAACCTCATCTCCCTCCTATAAATCGGAGCGAGACA

>R=55LTR#LTR (overlap with R=25)

TGTAAGAGATTATTCTTTTACATCATTGTCTCAAAGCTTGCTGGGGACACAGATGAACAAGTCCAAGCAAGTCCTGGGCAAGGGTTGTGAAATCCTTTGTCTGAGGGACACAGATGGACAAGGCCAGGGGCAACGAGAGAGAGATAAGCTGCAGCTTAATGGCCATGAAGCAGTCTTTTGTGTGGGCTTATCTCAAGGAAGATGGCCATCTCAGGAGGTGCTGCACATGACTCTTACCCAAGCGCCCAGGAATGCCACGATGGCAGAAGAAGAAGGATAAAAAGGGGACCTACAACCAGGAGTTTAGGTTTTCTGGCAACAGATTCCTCATGAAGAAGAAGAGGTTTCTGCCATGAGAAGCCTCACGACCTGCCTCTCTCCCTCCTGGACTTGCTCTGCGCTCTACCAGCATGCTGAAGAAGAACAAAGGTGTCTGCCATCAGATTCCTCACTACGGAATTCCTGCATGCTGACGGACTCTCCTGGGCCTGCTACCTGAGTCCTGCTGCTTCCTCCCGGATTGGCTCTGCGACTTCTTCCTGCTACCTGCTTGCTGCCCAAGGCCGGCCACGCTGCTGCTCTCTCCCCCCGCTGCTTTTGCCTCGGACCTTCGAAACGTCGTGCAACGGGACCGCTGCTGGATCCTGGTGGTGACTATCCCCGCTTTACGCAATTCTTGCTTCTTTCCTATCTTTTCTATCGCCCTCCTTCCCTTCCCCGTCTCCCTGATTAGGATTTATAATAAACTGGTTGGACCAACATTTGAACCGTTGTTTCTTAATCTCACGCCGGGTATACAGATATTAAAAGAACCTCATCTCCCTCCTATAAATTGGAGCGAGACA

>R=265LTR#LTR

CTTCAGTTCAAAGTAGGGCTTTTGGTTTCAGAAACTCTCTCTCTTATTTTATTTGATTTATTTAACTCAATTTCAATTATATTGTATTATATTGTGTTATCTTGCATTCCAATATCATATTTAGTAAAATTAAGTTTCCTCCTTAGATCGTTGCTGCTGTTCTGTTTTT

>R=623LTR#LTR

CATGGACTTATATCTAGGGATAACCCCGTGACAACTATTGGTGGAGGATGCGGGCAACTATGGTTTTTTACTTCATCTTAAGAGCATTATATAGAGGACTAAGGACCCTTATGTTCCTTGGAACATATTGGTTCCTTTTTAAGCTCCTCATATTCTGTTTCGGAGTTGCACATATGTATAGTCAATTAAGGGCACTGATGGAGATACCTGCCTGGTTCCTATACATCCCGGCTAATCTAACAGACAATATGATGTTTGGACTGGTTTCCAGGTGCTCTTCCCAGTTCCTTATGCATGTTCAGGGTG

>R=773LTR#LTR

GGATAACCCCGTGACAACTATTGGTGGAGAATGCGGGCAACTATGGTTTTTTACTTCATCTTAAGAGCATTATATAGAGGACTAAGGACCCTTATGTTCCTTGGAACATACTGGTTCCTTTTTAAGCTCCTCATATTCTGTTTCGGAGTTGCACATATGTATAGTCAATTAAGGGCACTGATGGAGATACCTGCCTGGTTCCTATACATCCCGGCTAATCTAACAGACAATATGATGTTTGGACTGGTTTCCAGGTGCTCTTCCCAGTTCCTTATGCATGTTCAGGGTGCCAGCTTAGGTACTG

>R=675LTR#LTR

TGAGAGTGTTGTCCTTCCAGGTATAGTCTGAAACTTTATTTGTGCGTACCTCCTGGAGTTGCAAGGAGCGATATTTTTGAAAACTTAGACGTGAGTCCTTCTCCAGGAAGACCGCTCCATATTCTGAAACTTTACTGTTTATGTGTATACCTCTCGGGGACGTATGAATTAATTTTGAATACGTGTGTGCCTCCTCAGGAAGACCTCTCTGCATTTTGTGATTTAAGTATCTTGTAACTTAAGTGTGAAATTTGAACCTTTTTGTGTGCTTCCCTCAGAGAAAGGGAAGAGTGATTGGAGCATTTGCATTTGTT

>R=42LTR#LTR

TGTCACGGAGTTAACGAGATCAATCTACGCCCGTGAAAAGGGGGCGGTAGGCCCCTGCCCTCCCCTCCCGGCCCCACAAAATGGGAAGGAAGGGAGGAAAACAAAAAAAGAGGCAACAATCTATGGAGGAAAACTTATTTTACTAACTATGATATCGGAATGCAAGATAACACAATATAATACAATCTGATTGAAATTGAGACTAATAAATCAAATAAAATAGGAGAGAGAGAGTGCCCGAGACCGATTCAAACCTGAAAAGCTTGGAACGGCCAGGAAAGCACCCTCCAGCACCCACTGCCAGGCAGTAAGAGACCGCCCCACCCGGAAGGGCCAGATCCCATGACTTCTGTAATGTATAGGGATAGGTGCCTGGAATTGGAGCATTAACACTTTAACTCCCAGTACACTACATGATGTTTTGATGCGGAATACTGAGAACCAAAAACAAAAAAACAAAAAACCATGACA

>R=39#DNA

CACTGTCACCACTGCTGAAATGCACCACCCACTACCTCACTGTGCTCACATCCACTGTTTGGTCTCCATAAACATTCAGCAAGCATTAATGAATGTCAATGGGTGACATTTTTTCTGCATGGAGGAATTCAATGACACACCTTTGCTTCATATGCACTTCCATGTCAGACACCATTTTGTCAGACTGCCCCTCTGCTGCCATCTGTCACATGGCAACAAAATGTAATGGAATATTGGTGGGAAGGTTCAACCTCTACTGCCATACCACCAACATCCGCCTCTGATGTTGTGGGCCAACATAATAAAATAGGAGGCATTACTTTTGGAGCAGCTCTCATAAAATATATAATATATATAATATATATATAAAAAAAAAATTAATTTTTTTTATTTTTTTTTTTTAAAAAAAAAAAAAAAAAGAGCAACAAAAACATAAAACTAGTGGGATATTTGACCTTGTTTTTGTGAAACTAATGCATCAGTCTGGTTTGATGGCAGTGGTTGCAATTCTTAGTGAGTTCTCAAGGTGTTCATCAGAGATTTTTGATGAAATTTTACTCTTCCTGTACTTCATCCTTGAAAATAGTTGTTCACAAATGTACATACTGCCAAAAAGTGATGACATGAATAAGGTGTGATTGTGAAGTGAGGGATATTTTTCTCTGGTAAGAGAGGGCTTATAAAAGTCTGGTAAAGAGACATGATCAGATTTTTGAGTTGAATATCTGATTGCAACTCTATACATTCCATTTGAAAATTTGCAGGTAATGTATTTATATTAACTGAAAATGGAGTCACAAATATACAAAAAAAATTTTTTTTTTTTTCTTGAATCTTGAAACCTATTCTCAAATTCCTTTATCAAAATAGAAAGCATGGCTGCATATTTTTCACTGTTCACAGGACTGTGTTTAGCCAGTGTATCAAAATGCATAAAATTATTTGCCATAACTTGAGTTAGCACTGCACT

>R=235#DNA

ATGAAGCAAAGATGTGTCACTGAATTCCTCCATGTGAAAAAAATGGCACCCATTGACATTCATCGATGCTTGCTGAACATTTATGGAGACCAAACAGTGGATGTGAGCACAGTGAGGTAGTGGGTGGTGTGTTTCAGCAGTGGCAACAGCAATGTGAAAGACAAGCCATGTTCCAGA

>R=119#DNA

AATGCCTCCTATTTTATTATGTTGGCCCACAATGTCAGAGGCAGATGGTGGTGGTATGGCAGTAGAGGTTGAAACTTCCCACCAATATTCCATTACATTTTGTTGCTGTGTGACAGATGGCAGCAGAGGGGCAGTCTGACAAAATGGTATCTGACATGGAAGTGCATATGAAGCAAAGGTGTGTAACTGAATTCCTCCATGAGGAAAAAATGGCACCCATTGACATTCATTGACACTTGCTGAATGTTTATGGAGACCAAACAGTGGATGTGAGCACAGTGAGGCAGTGGGTGGTGCATTTCAGCAGTGGTGACAACAACAGTGGGTCACCTCCACTGGTGCAGATTTTTATGAGCATGGCATGCAGGCTCTTGTTCATTGCTGGTGAAAATGCATAGCTAATGGTGGTGACTATGTTGAAAAATAGTGTTTTGTAGCTGAGAATTTGCTCTATCAAATAGTGTTATTGTGCTCTTTGTATCTGTTGTAGTTTCCATGGAAATAAATAGGAGGCATTACTTTCAGAGTGACCTAC

>R=88#DNA

ATGTAGGTTGCTCCAAAAGTAATGCCTCCTATTTATTTCCATGGAAACTACAACAGATACAAAGAGCACAATAACACTATTTGATAGAGCAAATTCTCAGCTACAAAACACTATTTTTCAACATAGTCACCACCATTAGCTATGCATTTTTACCAGCGATGAACAAGAGCCTGCATGCCATGCTCATAAAAATCTGCA

>R=32#DNA

AAGAAAAAGTTCAAGATGCAGCCCTCAGCAGGTAAAGTGATGTGCACTGTCTTTTGGGATAGGAAAGGGGTGATCCTTCTGGATTTCCTGGAACCCAGACAAACCATCAACTCTGACCACTACATCACAATGCTGACTAAGCTGAAGGCTCAAACTTCCAGAGTCAGGCCAGAGAAGAAGACAACCTTTCTCTTGCAACATGATAACACCAGGCCCCATACCAGTTTGAAGACCATGGAGCACATTGCCAATCTTGGCTGGACTGTCCTACCACACCCACCATATAGTCTGGATTTGGTACCTTCTGACTTCCATCTGTTTAGGCCGATGAAAGATGGACTGCATGGGCAACATTTTCCTAGCAATGATGCCATCATAGCAGCTGTGAAACAGTGGGTCACCTCCACTGGTGCAGATTTTTATGAGCATGGCATGCAGGCTCTTGTTCATCGCTGGTGAAAATGCATAGCTAATGGTGGTGACTATGTTGAAAAATAGTGTTTTGTAGCTGAGAATTTGCTCTATCAAATAGTGTTATTGTGCTCTTTGTATCTGTTGTAGTTTCCATGGAAATAAATAGGAGGCATTACTTTCAGAGCAACCTATGTA

>R=26#DNA

TATGTAGGTTGCTCTGAAAGTAATGCCTCCTATTTATTTCCATGGAAACTACAACAGATACAAAGAGCACAATAACACTATTTGATAGAGCAAATTCTCAGCTACAAAACACTATTTTTCAACATAGTCATCATAATTTGCCAATTCATATGGATGAGCTGATCAAGACACTCTTCATTTTGTGGTGTGACAGCTGTGCATGGCCATCCAGAACATGGCTTGTCTTTCACATTGCTGTCACCACTGCTGAAATGCACCACCCACCACCTCACTGTGCTCACATCCACTGTTTGGTCTCCATAAATGTTCAGCAAGCATCAATGAATGTCAGTGGGTGCCATTTTTTCCACATGGAGGAATTCAGTGACACACCTTTGCTTCATATGCACTTCCATGTCAGATGCCATTTTGTCAGACTGCCCCTCTGCTGCCATCTGTCACACAGCAACAAAATGTAATGGAATATTGGTGGGAAGGTTCAACCTCTACTGCCATACCACCAACATCCGCCTCTGATGTTGTGGGCCAACATAATAAAATAGGAGGCATTACTTTTAGAGCAGCCCTTATAT

>R=396#DNA

CATGGGTGTCCAACCTTTTGGCTTGCCTGGGCTGCACTGAGTGAAGAGGAATTGTCTTGGGCTGCATATA

>R=208#DNA

CAGTGACAGTGGGTCACCTCTGCTGGTGCAGATTTTTATGAGCGTAGCATGCAGGCTCTTGTTCATTGCTGGCAAAAATGCATAGCTAATGGTGGTGACTATGTTGAAAAATAGTGTTTTGTAGCTGAGAATTTGCTCTATCAAATAGTGTTATTGTGCTCTTTGTATCTGTTGTAGTTTCCATGGAAATAAATAGGAGGCATTACTTTCAGAGTGACCTATGTA

>R=48#DNA

AAAGTAATGCCTCCTATTTTATTATGTTGGCCCACGACATCAGAGGCGGATGTTGGTGGTATGGCAGTAGAGGTTGAACCTTCCCACCAATATTCCATTACATTTTGTTGCCGTGTGACAGATGGCAGCAGAGGGGCAGTCTGACAAAATGGCATCTGACATGGAAGTGCATATGAAGCAAAGGTGTGTCACTGAATTCCTCCATGTGGAAAAAATGGCACCCATTGACATTCATTGATACTTGCTGAATATTTATGGAGACCAAACAGTGGATGTGAGCACAGTGAGGCGGTGGGTGGTGTGTTTCAGCAGTGGTGACAGTGACATGAAAGACAAGCCACATTCCAGACGGCCATGCACAGCTGTCACACCATGAAATGAAGAGTATCTCAATCAGCTCATCCACGCAAATTAGCAGATTATGACCAGGGAACTGTGTATAGAGCTGAATATCAGCTTCAATGCATTGGAAATGATGGTGGCAACATTGGAATATCACAAAGTTTGCACCAGGTGGGTCCCACGAATGCTCACACAGGAACAGAAAGAACACCATATGCAAGTTTGTCAGGACCTATTGAACCAATATGAGGCTGAAGGTGACAGTTTCCTGGATCACATCATTACTAGTGATGAGATGTGGTGTCACCACTATGAGCCAGAGTCAAAACAGCAGTCCATGGAGTGGTGACATGTGAATTCCCCATCAAAGAAAAAGTTCAAGATGCAGCCCTCAGCGGGTAAAGTGATGTGCACTGTCTTTTGGGATAGGAAAGGGGTGATCCTTCTGGATTTCCTGGAACCCAGACAAACCATCAACTCTGACTACTACATCATAACGCTGACTAAGCTGAAGGCTCAAACTTCCAGAGTCAGGCCAGAGAAGAAGACAACCTTTCTCTTGCAACATAATAATACCAGGCCCCATACCAGTTTGAAGACCATGGAGCACACTGCCAATCTTGGCTGGACTGTCCTACCACACCCACTGTATAGTCTAGATTTGGTGCCTTCTGACTTCCATCTGTTCAGGCTAATGAAAGATGGACTGCGTGGGCAACATTTTCCTAGCAACGATGCCATCATAGCAGCTGTGAAACAGTGGGTCACCTCCGCTGGTGCAGATTTTTACGAGTGCAGCATGCAGGCTCTTGTTCATCGCTGGTAAAAATGCATAGCTAATGGTGGTAACTAT

>R=3#DNA

TATGAGGGCTGCTCTGAAAGTAATGCCTCCTATTTTATTATGTTGGCCCACAATGTCAGAGGCAGATGTTGGTGGTATGGCAGTAGAGGTTGAACCTTCCCACCAATATTCCATTACATTTTGTTGCTGTGTGACAGATGGCAGCAGAGGGGCAGTCTGACAAAATGGCATCTGACATGGAAGTGCGTATGAAGCAAAGGTGTGTCACTGAATTCCTCCATGTGGAAAAAATGGCACCCACTGACATTCATTGATGCTTGCTGAACATTTATGGAGACCAAACAGTGGATGTGAGCACAGTGAGGTAGTGGGTGGTGTGTTTCAGCAGTAGCAACAGCAACAGTGGGTCACCTCCACTGGTGCAGATTTTTATGAGCATAGCATGCAGGCTCTTGTTCATTGCTGGTGAAAATGCATAGCTAATGGTGGTGACTATGTTGAAAAATAGTGTTTTGTAGCTGAGAATTTGCTCTATCAAATAGTGTTATTGTGCTCTTTGTATCTGTTGTAGTTTCCATGGAAATAAATAGGAGGCATTACTTT
