# Supplementary figures and images for: Multi-Platform Next-Generation Sequencing of the Domestic Turkey (Meleagris gallopavo): Genome Assembly and Analysis
Source: PLoS Biol. 2010 Sep 7;8(9):e1000475. doi: 10.1371/journal.pbio.1000475 (PMC2935454; doi:10.1371/journal.pbio.1000475)

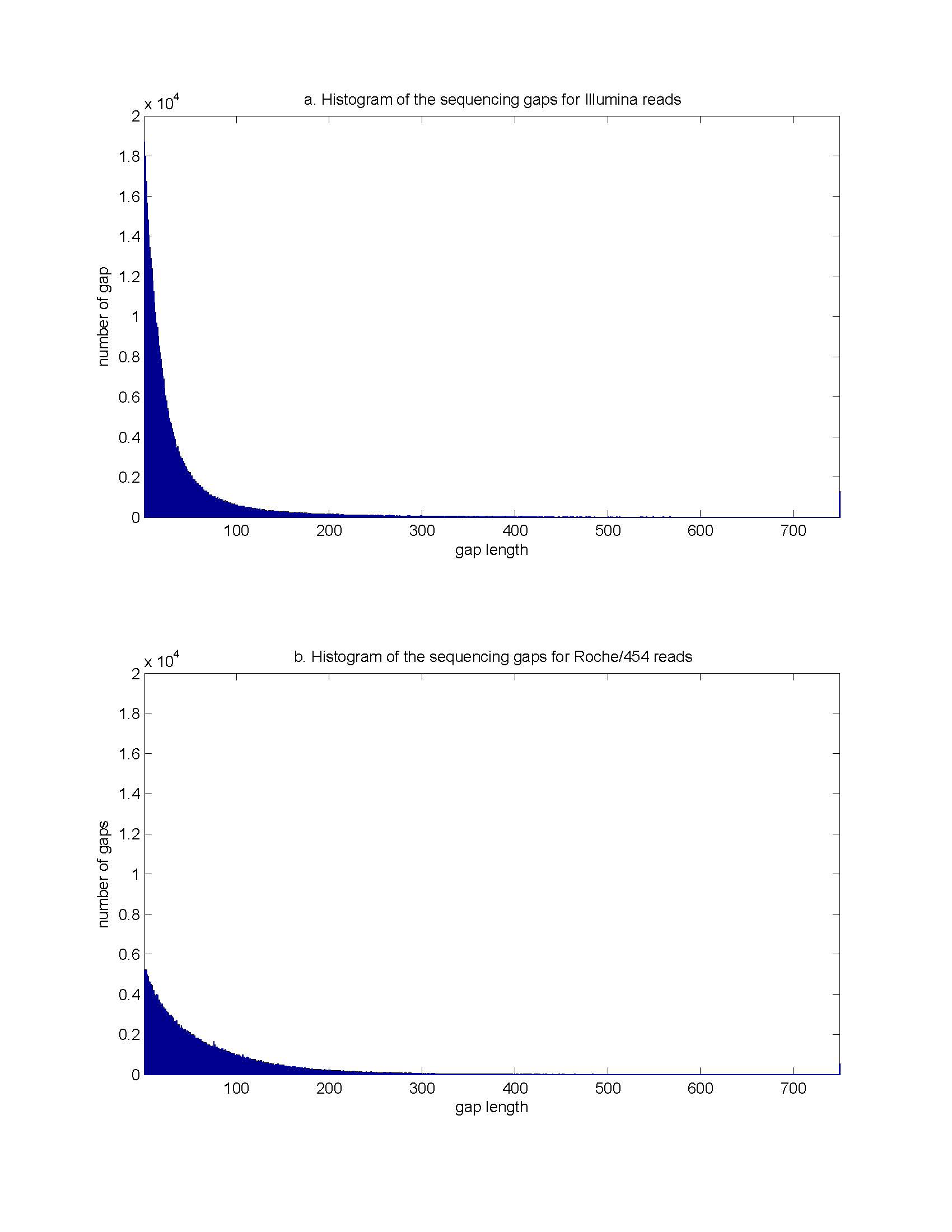

Supplement: Figure S1 — Distribution of regions of exclusive coverage for both sequencing platforms. Panel (a) shows a large number of short (<20 bp) gaps in coverage by Illumina sequencing, whereas the Roche/454 coverage gaps tended to be larger as shown in panel (b). The mean sequencing gap for Illumina reads was 46 bases compared to a 72 base mean gap for Roche/454 coverage. (0.53 MB TIF) [file pbio.1000475.s002.tif]

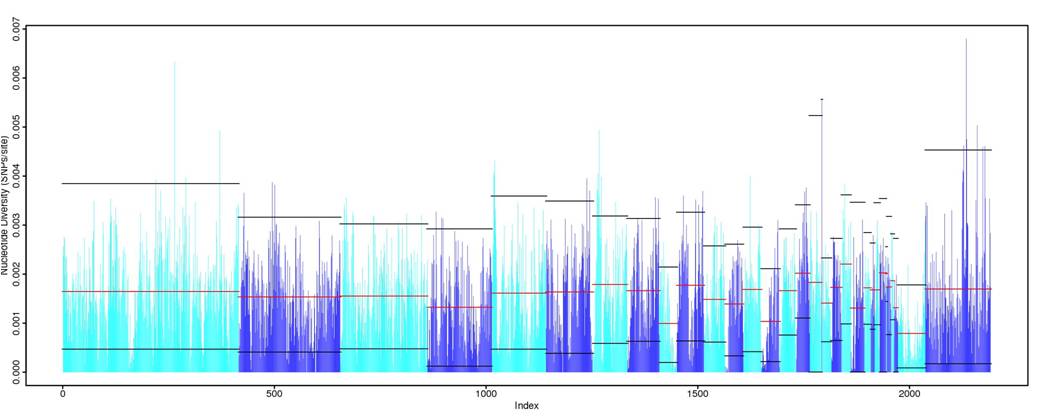

Supplement: Figure S2 — SNP identification and estimates of nucleotide diversity across the turkey genome. (0.05 MB JPG) [file pbio.1000475.s003.jpg]

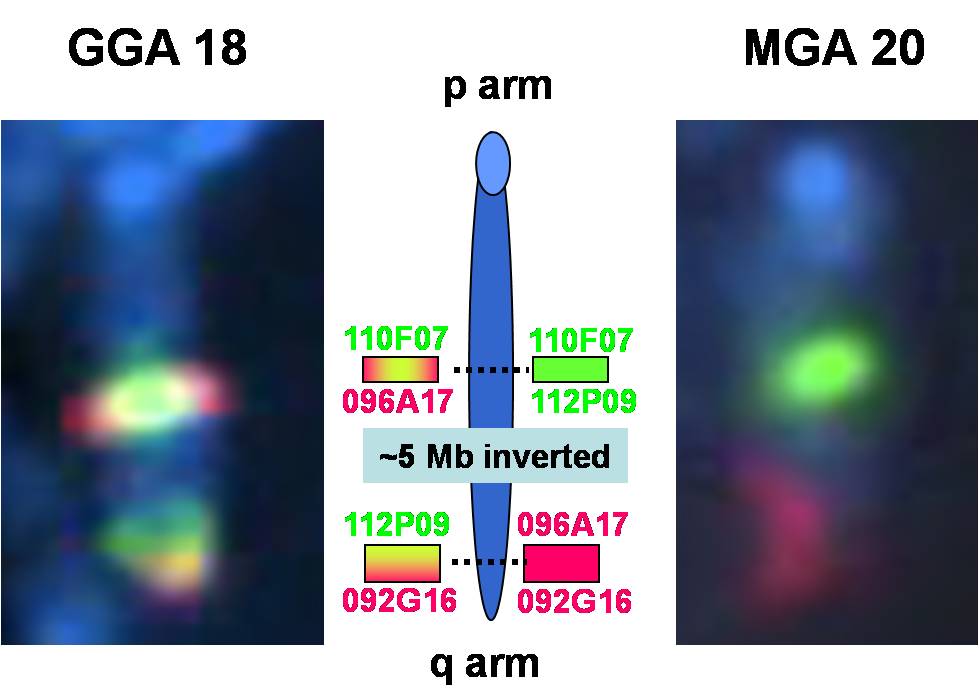

Supplement: Figure S3 — FISH confirmation of the turkey-chicken inversion rearrangement due to apparent unequal recombination between NME1 and NME2 orthologs on GGA18/MGA20. CHORI-260 BACs 110F07 (GGA18 end coordinates: 4,850,650–5,056,016) and 112P09 (9,665,995–9,865,995) were labeled in green (FITC), while 96A17 (5,087,535–5,266,203) and 92G16 (9,980,713–10,142,396) were labeled with red (Enzo Red) and used for FISH analysis of chicken and turkey pachytene chromosomes, which are 14–20× more extended than mitotic metaphase chromosomes allowing for greater resolution. A view of GGA18 (left frame) affirms the arrangement predicted by the BES alignments noted above, 110F07 and 96A17 signals co-localize to generate a yellow signal halfway along the chromosome q arm, as do 112P09 and 92G16 near the q terminus. Whereas for MGA20 (right frame), the 110F07 and 112P09 BAC probes co-localize (green) as do the two red probes, indicative of the 5 Mb inversion. (Prior FISH experiments utilized the BAC probes singly or in pairs of two to ensure all probes hybridized equally well.) This inversion was previously indicated by inconsistent BAC mate pairs: CHORI-260 111D05 (5,106,305–10,099,832), 95I22 (5,109,664–10,107,855), 89F20 (5,134,762–10,035,123), 94C02 (5,157,115–10,042,702), and 95H13 (5,268,786–9,982,916) and 78TKNMI 18A07 (5,109,437–10,066,115), all of which had BES that aligned with the same strand in the chicken sequence, as expected for BACs that cross inversion breakpoints. Additional FISH, overgo mapping, and fingerprint analyses confirm the inversion and narrow the breakpoint regions to sites near the NME1 and NME2 orthologs (unpublished data). (0.06 MB JPG) [file pbio.1000475.s004.jpg]

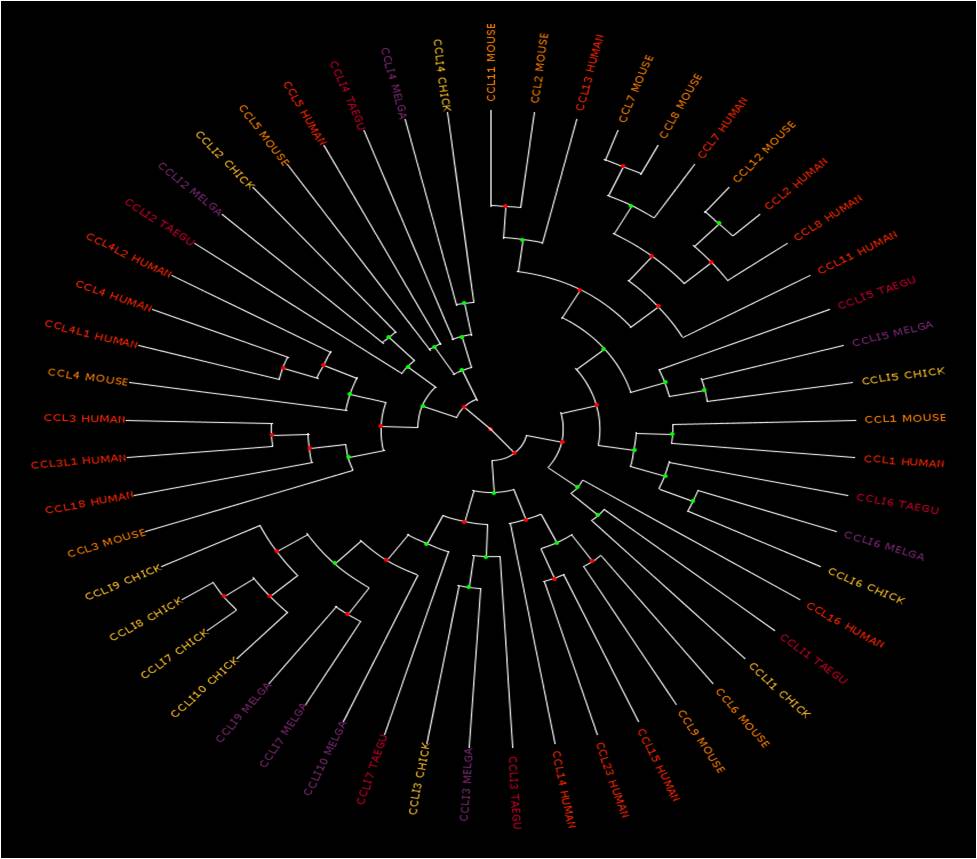

Supplement: Figure S4 — Evolution of the CCL gene family of chemokines. (0.08 MB JPG) [file pbio.1000475.s005.jpg]

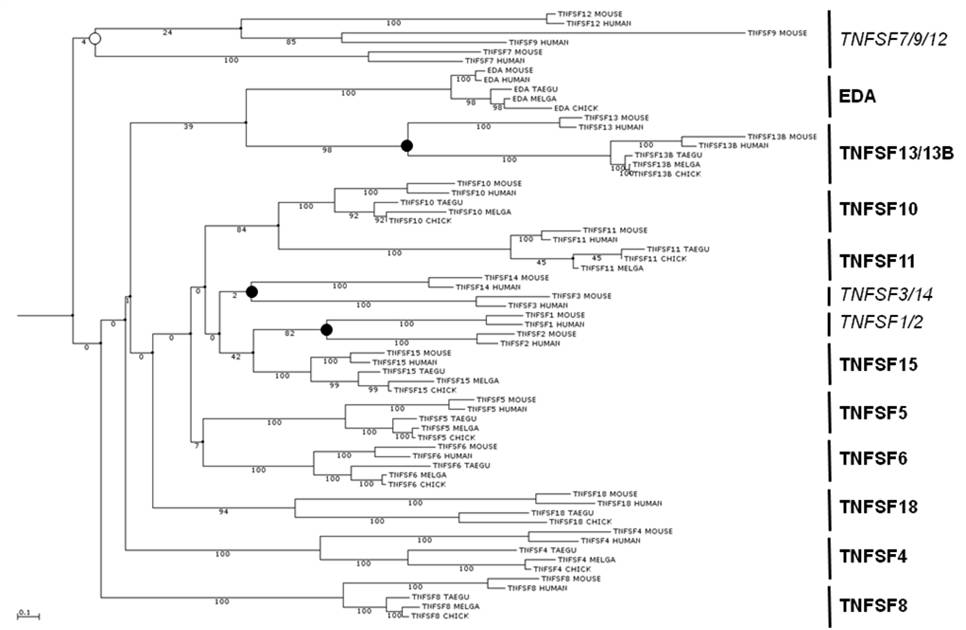

Supplement: Figure S5 — Evolution of TNF superfamily of ligands. (0.05 MB JPG) [file pbio.1000475.s006.jpg]

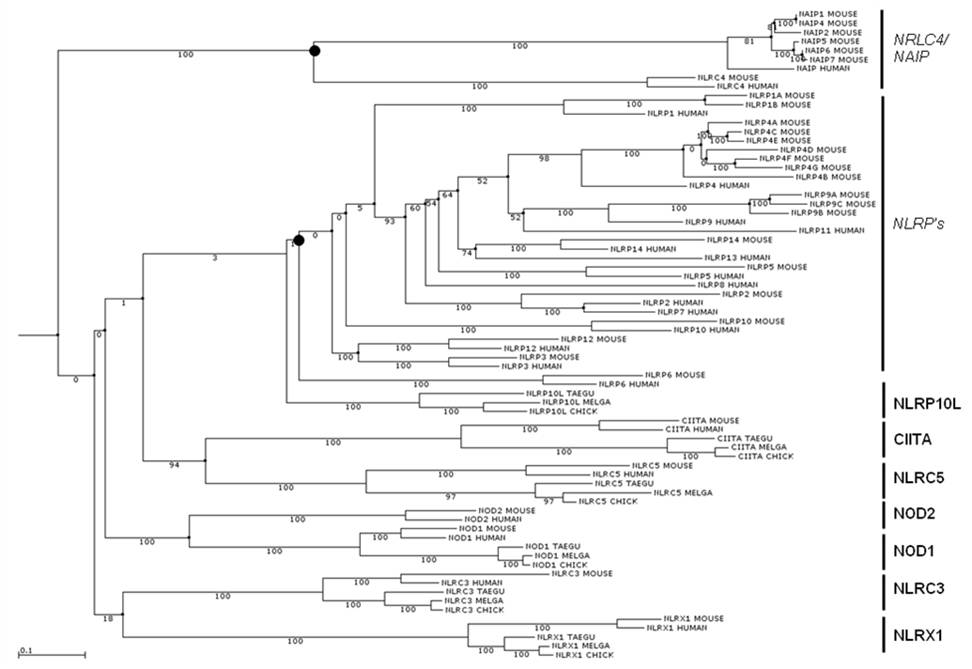

Supplement: Figure S6 — Evolution of NOD-like receptor gene families. (0.06 MB JPG) [file pbio.1000475.s007.jpg]

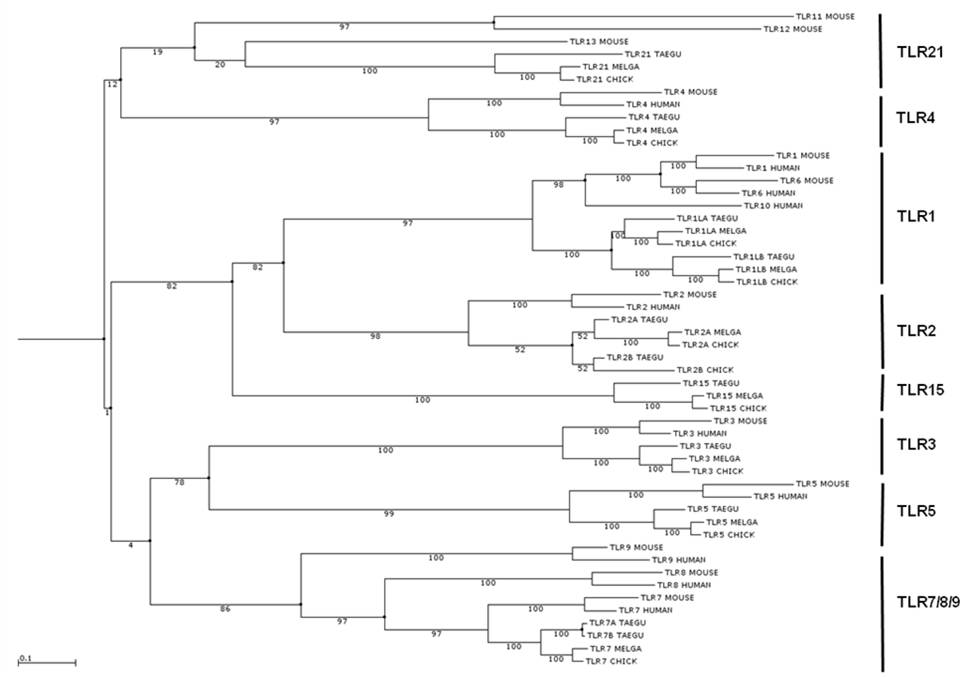

Supplement: Figure S7 — Evolution of the Toll-like receptor gene family. (0.05 MB JPG) [file pbio.1000475.s008.jpg]

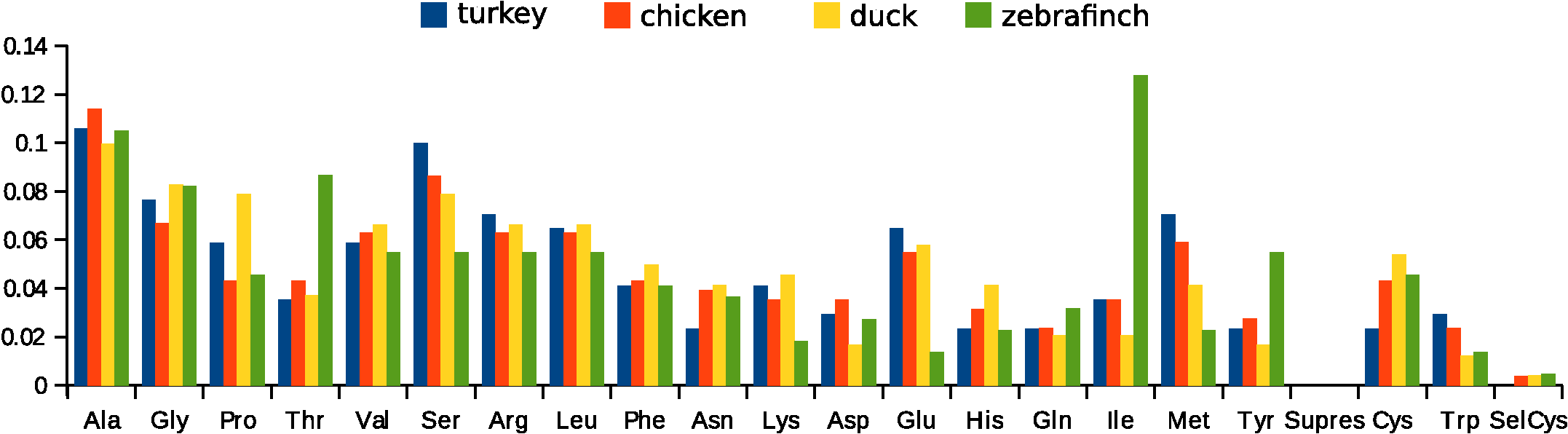

Supplement: Figure S8 — Codon usage in percent for M. gallopavo (turkey, blue), G. gallus (chicken, orange), A. platyrhynchos (duck, yellow), and T. guttata (zebra finch, green). (0.07 MB TIF) [file pbio.1000475.s009.tif]

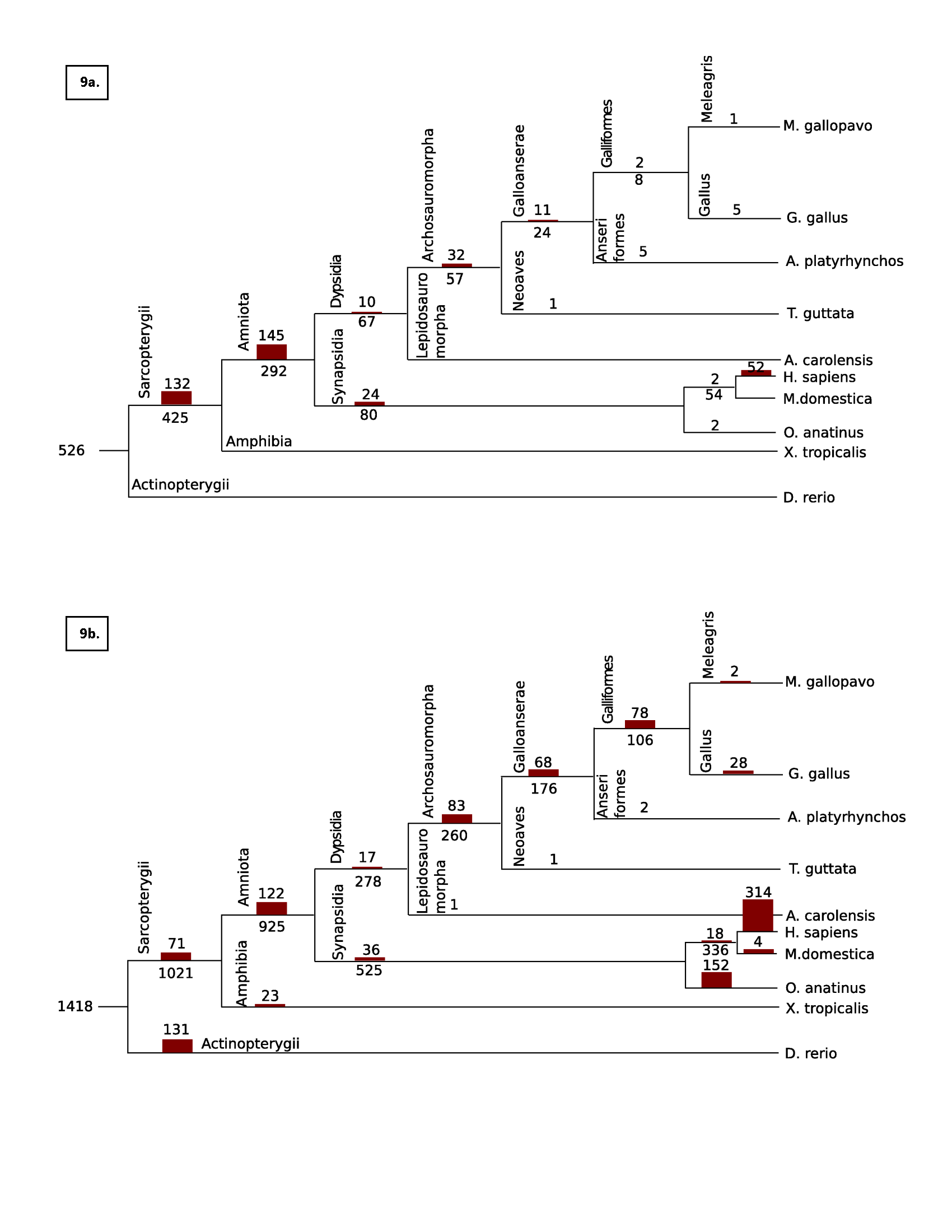

Supplement: Figure S9 — Lost and gained snoRNAs (a) and miRNAs (b) in different species. (0.24 MB TIF) [file pbio.1000475.s010.tif]

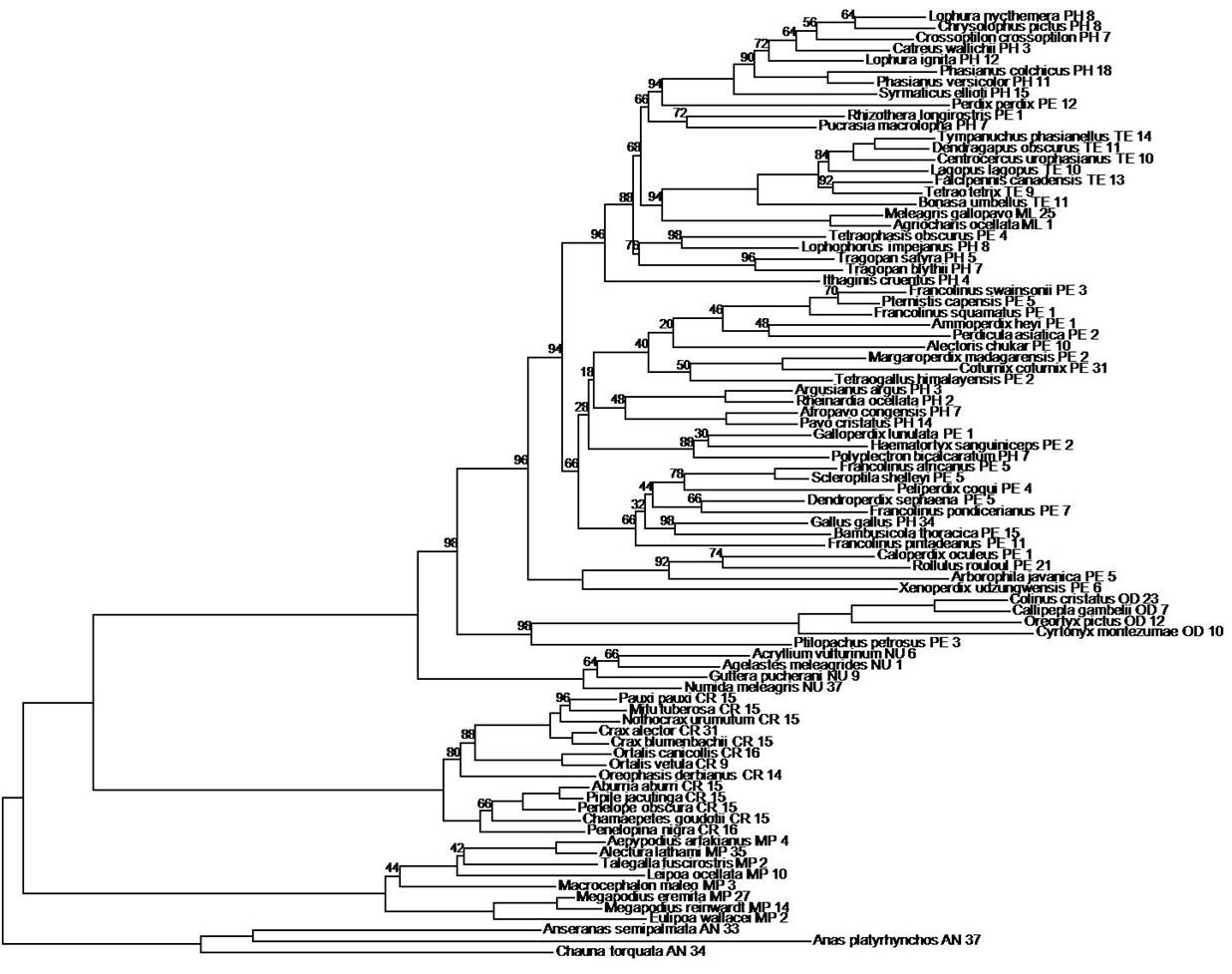

Supplement: Figure S10 — Turkey phylogeny: Maximum likelihood tree of Galliformes based on concatenated, partitioned alignment of DNA sequences for 42 loci (11 mitochondrial). Each species is marked with a two-letter abbreviation of its NCBI order (AN, Anseriformes outgroup), family (MP, Megapodiidae; CR, Cracidae; NU, Numididae; OD, Odontophoridae), or phasianid subfamily (PE, Perdicinae; PH, Phasianinae; TE, Tetraoninae; ML, Meleagridinae), followed by the number of loci used for the species. Bootstrap support percentages are shown if <100%. (0.15 MB JPG) [file pbio.1000475.s011.jpg]
