# Supplementary material for: Multi-Platform Next-Generation Sequencing of the Domestic Turkey (Meleagris gallopavo): Genome Assembly and Analysis
Source: PLoS Biol. 2010 Sep 7;8(9):e1000475. doi: 10.1371/journal.pbio.1000475 (PMC2935454; doi:10.1371/journal.pbio.1000475)
Supplement: Table S4 — Summary of gene family assignments. (0.03 MB DOC) [file pbio.1000475.s015.doc]

**Table S4.** Summary of gene family assignments.

|  | **Turkey** | **Chicken** |
| --- | --- | --- |
| Gene loci | 15093 | 16736 |
| Proteins | 16217 | 22194 |
| family | 8535 | 12188 |
| **In turkey** | | |
| Innovation family^1^ | | 485 |
| Extinction family^2^ | | 4138 |
| Expansion family^3^ | | 539 |
| Contraction family^4^ | | 780 |
| No change^5^ | | 6731 |

^1^Innovation family is the family found in turkey but not in chicken. ^2^Extinction family is the family found in chicken but not in turkey. ^3^Expansion: more members in turkey than in chicken. ^4^Contraction: more members in chicken than in turkey. ^5^No change: same number of members in chicken and turkey.
