# Supplementary material for: Multi-Platform Next-Generation Sequencing of the Domestic Turkey (Meleagris gallopavo): Genome Assembly and Analysis
Source: PLoS Biol. 2010 Sep 7;8(9):e1000475. doi: 10.1371/journal.pbio.1000475 (PMC2935454; doi:10.1371/journal.pbio.1000475)
Supplement: Table S5 — Gene family comparisons between turkey and chicken. (0.14 MB DOC) [file pbio.1000475.s016.doc]

**Table S5a.** Gene family expansion in turkey compared to chicken based on comparative genome alignments.

| **Family ID** | **Turkey** | **Chicken** | **Log ratio** | **Family description** |
| --- | --- | --- | --- | --- |
| ENSFM00500000273095 | 4 | 1 | 1.39 | Arginine/Serine Rich Coiled Coil 1 |
| ENSFM00560000775633 | 4 | 1 | 1.39 | Unknown |
| ENSFM00250000005228 | 3 | 1 | 1.1 | Conserved Oligomeric Golgi Complex Subunit 2 Cog Complex Subunit 2 Component of Oligomeric Golgi Complex 2 Low Density Lipoprotein Receptor Defect C Complementing |
| ENSFM00440000236854 | 3 | 1 | 1.1 | Anion Exchange 2 Anion Exchanger 2 Ae 2 Solute Carrier Family 4 Member 2 |
| ENSFM00560000771013 | 3 | 1 | 1.1 | Ubiquitin Carboxyl Terminal Hydrolase 2 Ec_3.1.2.15 Ubiquitin Thioesterase 2 Ubiquitin Specific Processing Protease 2 Deubiquitinating Enzyme 2.41 Kda Ubiquitin Specific Protease |
| ENSFM00250000002761 | 3 | 1 | 1.1 | Sec14 Domain And Spectrin Repeat Containing 1 |
| ENSFM00250000003026 | 3 | 1 | 1.1 | Non Specific Lipid Transfer Ec_2.3.1.176 Propanoyl Coa C Acyltransferase Nsl Tp Sterol Carrier 2 Scp 2 Sterol Carrier X Scp X Scp Chi Scpx |
| ENSFM00250000003114 | 3 | 1 | 1.1 | Regulatory Associated Of Mtor Raptor P150 Target Of Rapamycin Tor Scaffold |
| ENSFM00250000002520 | 3 | 1 | 1.1 | Type I Inositol 1 4 5 Trisphosphate 5 Phosphatase 5ptase Ec_3.1.3.56 |
| ENSFM00250000007415 | 3 | 1 | 1.1 | Thrombopoietin Receptor Precursor Tpo R Myeloproliferative Leukemia C Mpl Cd110 Antigen |
| ENSFM00250000005041 | 3 | 1 | 1.1 | Autophagy 5 |
| ENSFM00560000771032 | 3 | 1 | 1.1 | Cytoplasmic Dynein 2 Heavy Chain 1 Cytoplasmic Dynein 2 Heavy Chain Dynein Cytoplasmic Heavy Chain 2 Dynein Heavy Chain Isotype 1b Dynein Heavy Chain 11 |
| ENSFM00470000251449 | 3 | 1 | 1.1 | Caprin 1 Cytoplasmic Activation and Proliferation Associated 1 Gpi Anchored Membrane 1 Gpi Anchored P137 P137gpi Membrane Component Chromosome 11 Surface Marker 1 |
| ENSFM00410000138497 | 3 | 1 | 1.1 | Potassium Channel Subfamily K Member Acid Sensitive Potassium Channel Task Twik Related Acid Sensitive K + Channel Two Pore Potassium Channel Kt3 |
| ENSFM00250000004914 | 3 | 1 | 1.1 | Sodium/Bile Acid Cotransporter 7 Na + /Bile Acid Cotransporter 7 Solute Carrier Family 10 Member 7 |
| ENSFM00270000056583 | 3 | 1 | 1.1 | Wd Repeat Containing 25 |
| ENSFM00500000271652 | 3 | 1 | 1.1 | Transmembrane and Tpr Repeat Containing 1 |
| ENSFM00250000007061 | 3 | 1 | 1.1 | Polycomb Group Asxl3 Additional Sex Combs 3 |
| ENSFM00500000412127 | 3 | 1 | 1.1 | Unknown |
| ENSFM00500000379074 | 3 | 1 | 1.1 | Unknown |

#### Table S5b. Gene family contractions in the turkey compared to chicken based on comparative genome alignments.

| **Family ID** | **Turkey** | **Chicken** | **Log ratio** | **Family description** |
| --- | --- | --- | --- | --- |
| ENSFM00250000002130 | 2 | 38 | -2.94 | Gc Rich Sequence Dna Binding Factor Gcf Transcription Factor 9 Tcf 9 |
| ENSFM00500000271760 | 1 | 18 | -2.89 | Uncharacterized |
| ENSFM00540000717901 | 16 | 201 | -2.53 | Olfactory Receptor Fragment |
| ENSFM00500000270329 | 7 | 84 | -2.48 | Uncharacterized |
| ENSFM00500000269614 | 1 | 8 | -2.08 | Trypsin Precursor Ec_3.4.21.4 |
| ENSFM00250000000837 | 1 | 6 | -1.79 | Helicase Ec_3.6.1 |
| ENSFM00500000269683 | 1 | 5 | -1.61 | Udp Glucuronosyltransferase 1 Precursor Udpgt Ugt1 Ugt1 |
| ENSFM00500000269670 | 3 | 15 | -1.61 | Class I Histocompatibility Antigen F10 Alpha Chain Precursor B F Histocompatibility F10 Antigen B F Beta Iv B12 |
| ENSFM00560000772443 | 1 | 5 | -1.61 | Ambiguous |
| ENSFM00420000140541 | 1 | 4 | -1.39 | Olfactory Receptor Olfactory Receptor |
| ENSFM00250000001319 | 1 | 4 | -1.39 | Homeobox Hox |
| ENSFM00250000000852 | 1 | 4 | -1.39 | Guanylate Binding Guanine Nucleotide Binding Gtp Binding Gbp |
| ENSFM00250000001149 | 1 | 4 | -1.39 | Alpha N Acetylgalactosaminide Alpha 2 6 Sialyltransferase Ec_2.4.99.- Galnac Alpha 2 6 Sialyltransferase St6galnac Sialyltransferase |
| ENSFM00500000269599 | 2 | 8 | -1.39 | A Disintegrin And Metalloproteinase With Thrombospondin Motifs Precursor Adamts Adam Ts Adam Ec_3.4.24 |
| ENSFM00500000270739 | 1 | 4 | -1.39 | Arylacetamide Deacetylase Ec_3.1.1 |
| ENSFM00250000004844 | 1 | 4 | -1.39 | Trafficking Particle Complex Subunit 10 Transport Particle Subunit Tmem1 Trapp Subunit Tmem1 Trafficking Particle Complex Subunit Tmem1 |
| ENSFM00260000050690 | 3 | 12 | -1.39 | B G |
| ENSFM00500000288227 | 1 | 4 | -1.39 | Unknown |
| ENSFM00560000772457 | 2 | 7 | -1.25 | Unknown |
| ENSFM00500000269594 | 3 | 9 | -1.10 | Histone H2A |

**Table S5c.** Gene families absent in the turkey genome sequence compared to chicken.

| **Family ID** | **Turkey** | **Chicken** | **Difference** | **Family description** |
| --- | --- | --- | --- | --- |
| ENSFM00560000771494 | 0 | 26 | 26 | Unknown |
| ENSFM00500000275326 | 0 | 15 | 15 | Unknown |
| ENSFM00420000176277 | 0 | 11 | 11 | Chloramphenicol acetyltransferase |
| ENSFM00250000000317 | 0 | 10 | 10 | Olfactory receptor |
| ENSFM00500000279500 | 0 | 8 | 8 | Unknown |
| ENSFM00500000280223 | 0 | 7 | 7 | Unknown |
| ENSFM00500000281216 | 0 | 6 | 6 | Unknown |
| ENSFM00500000284433 | 0 | 5 | 5 | Unknown |
| ENSFM00450000242139 | 0 | 4 | 4 | Protocadherin beta precursor [PCDH beta] |
| ENSFM00500000286095 | 0 | 4 | 4 | Unknown |
| ENSFM00500000286415 | 0 | 4 | 4 | Unknown |
| ENSFM00500000279068 | 0 | 4 | 4 | Unknown |
| ENSFM00500000288837 | 0 | 4 | 4 | Unknown |
| ENSFM00250000004395 | 0 | 3 | 3 | Golgi phosphoprotein 3 |
| ENSFM00500000269866 | 0 | 3 | 3 | Homeobox HOX |
| ENSFM00250000002244 | 0 | 3 | 3 | Neurexophilin precursor |
| ENSFM00550000743191 | 0 | 3 | 3 | Uncharacterized |
| ENSFM00250000008915 | 0 | 3 | 3 | Precursor |
| ENSFM00250000001072 | 0 | 3 | 3 | Homeobox CUT 1 CCAAT displacement CDP |
| ENSFM00250000000881 | 0 | 3 | 3 | Neuroblast differentiation associated AHNAK desmoyokin |

**Table S5d.** Gene families absent in chicken genome sequence compared to turkey.

| **Family ID** | **Turkey** | **Chicken** | **Difference** | **Family description** |
| --- | --- | --- | --- | --- |
| ENSFM00500000274184 | 1 | 0 | 1 | Multiple myeloma tumor associated 2 |
| ENSFM00500000286535 | 1 | 0 | 1 | Signal recognition particle 9 kDa SRP9 |
| ENSFM00500000271985 | 1 | 0 | 1 | Zinc transporter 1 [ZnT 1] solute carrier family 30 member 1 |
| ENSFM00500000273777 | 1 | 0 | 1 | Serta domain containing 4 |
| ENSFM00440000236908 | 1 | 0 | 1 | CXC chemokine receptor type 7 [CXCR7], G coupled receptor RDC1, chemokine orphan receptor 1 |
| ENSFM00500000272368 | 1 | 0 | 1 | Phosphatidylinositol N acetylglucosaminyltransferase subunit C ec_2.4.1.198 phosphatidylinositol glycan biosynthesis class C pig C |
| ENSFM00250000006574 | 1 | 0 | 1 | Unknown |
| ENSFM00500000273639 | 1 | 0 | 1 | Brain 44 |
| ENSFM00500000270386 | 1 | 0 | 1 | Mannose 1 phosphate guanyltransferase alpha ec_2.7.7.13 GDP mannose 1 phosphate guanylyltransferase GDP mannose pyrophosphorylase A |
| ENSFM00500000272176 | 1 | 0 | 1 | DNAJ homolog subfamily b member |
| ENSFM00500000270580 | 1 | 0 | 1 | Nuclear receptor subfamily 1 group I member 3 constitutive androstane receptor [CAR] |
| ENSFM00250000004211 | 1 | 0 | 1 | WDrepeat containing |
| ENSFM00500000270183 | 1 | 0 | 1 | High affinity interleukin 8 receptor a [IL-8Ra], CXCR 1, CD181 antigen |
| ENSFM00250000001219 | 1 | 0 | 1 | T cell surface glycoprotein precursor antigen |
| ENSFM00500000270039 | 1 | 0 | 1 | T complex 1 subunit gamma [TCP 1-gamma] CCT gamma |
| ENSFM00250000008285 | 1 | 0 | 1 | Transmembrane 79 |
| ENSFM00250000002759 | 1 | 0 | 1 | Farnesyl pyrophosphate synthetase FPP synthetase FPS farnesyl diphosphate synthetase [includes dimethylallyltranstransferase ec_2.5.1.1; geranyltranstransferase ec_2.5.1.- 10] |
| ENSFM00250000007988 | 1 | 0 | 1 | Inducible T cell costimulator precursor activation inducible lymphocyte immunomediatory molecule CD278 antigen |
| ENSFM00500000270869 | 1 | 0 | 1 | Fad synthetase ec_2.7.7.2 FMN adenylyltransferase FAD pyrophosphorylase flavin adenine dinucleotide synthetase [includes molybdenum cofactor biosynthesis region; FAD synthetase region] |
| ENSFM00550000743770 | 1 | 0 | 1 | S100 A10, S100 calcium binding A10 calpactin 1 light chain, calpactin I light chain P10 P11 cellular ligand of Annexin II |

#### 
