# Supplementary material for: Multi-Platform Next-Generation Sequencing of the Domestic Turkey (Meleagris gallopavo): Genome Assembly and Analysis
Source: PLoS Biol. 2010 Sep 7;8(9):e1000475. doi: 10.1371/journal.pbio.1000475 (PMC2935454; doi:10.1371/journal.pbio.1000475)
Supplement: Table S6 — Summary of gene families in 16 animal species. (0.05 MB DOC) [file pbio.1000475.s017.doc]

**Table S6.** Summary of gene families in 16 animal species.

|  | **human** | **chimp** | **macaca** | **mouse** | **rat** | **opossum** | **dog** | **pig** |
| --- | --- | --- | --- | --- | --- | --- | --- | --- |
| **Genes*** | 22366 | 19816 | 21892 | 23104 | 22925 | 19453 | 19292 | 17480 |
| **Gene families^†^** | 15208 | 13588 | 14346 | 14721 | 14094 | 11993 | 12571 | 11242 |
| **Genes per family** | 1.47 | 1.46 | 1.53 | 1.57 | 1.63 | 1.62 | 1.53 | 1.55 |
| **Singleton gene families** | 2648 | 1393 | 2853 | 2970 | 2682 | 1960 | 1564 | 1765 |
| **Gene families^‡^** | 12560 | 12195 | 11493 | 11751 | 11412 | 10033 | 11007 | 9477 |
| **Genes per family** | 1.57 | 1.51 | 1.66 | 1.71 | 1.77 | 1.74 | 1.61 | 1.66 |
|  | **cow** | **zebrafish** | **fugu** | **lizard** | **fruit fly** | **chicken** | **turkey** | **zebra finch** |
| **Genes*** | 21035 | 24134 | 18510 | 17472 | 14063 | 16736 | 15093 | 17475 |
| **Gene families^†^** | 12810 | 12458 | 10414 | 11488 | 11556 | 12188 | 8535 | 11216 |
| **Genes per family** | 1.64 | 1.94 | 1.78 | 1.52 | 1.22 | 1.37 | 1.77 | 1.56 |
| **Singleton gene families** | 1591 | 3318 | 1879 | 2555 | 7745 | 2778 | 27 | 2291 |
| **Gene families^‡^** | 11219 | 9140 | 8535 | 8933 | 3811 | 9410 | 8508 | 8925 |
| **Genes per family** | 1.73 | 2.28 | 1.95 | 1.67 | 1.66 | 1.48 | 1.42 | 1.70 |

* excludes mitochondria genes. **^†^**includes singleton gene families. **^‡^** excludes singleton gene families
