# Supplementary material for: Multi-Platform Next-Generation Sequencing of the Domestic Turkey (Meleagris gallopavo): Genome Assembly and Analysis
Source: PLoS Biol. 2010 Sep 7;8(9):e1000475. doi: 10.1371/journal.pbio.1000475 (PMC2935454; doi:10.1371/journal.pbio.1000475)
Supplement: Table S7 — Species-specific RPG. (0.03 MB DOC) [file pbio.1000475.s018.doc]

**Table S7. Species-specific RPG***

| **Pattern (all 16 species)** | **# of families** |
| --- | --- |
| other species < turkey/chicken | 881 |
| turkey/chicken < other species | 70 |
| other species < turkey/chicken/zebra finch | 271 |
| turkey/chicken/zebra finch < other species | 33 |

*RPG: rate pattern group
