# Supplementary material for: Multi-Platform Next-Generation Sequencing of the Domestic Turkey (Meleagris gallopavo): Genome Assembly and Analysis
Source: PLoS Biol. 2010 Sep 7;8(9):e1000475. doi: 10.1371/journal.pbio.1000475 (PMC2935454; doi:10.1371/journal.pbio.1000475)
Supplement: Table S8 — Enrichment test of KEGG pathway. (0.04 MB DOC) [file pbio.1000475.s019.doc]

**Table S8. Enrichment test of KEGG pathway.**

|  | **Mg (Turkey)** | | |  | **Gg (Chicken)** | | |
| --- | --- | --- | --- | --- | --- | --- | --- |
| KEGG_PATHWAY | Count | % | *P* value |  | Count | % | *P* value |
| Regulation of actin cytoskeleton | 14 | 3.59% | 0.002 |  |  |  |  |
| Axon guidance | 9 | 2.31% | 0.016 |  |  |  |  |
| MAPK signaling pathway | 13 | 3.33% | 0.031 |  |  |  |  |
| Focal adhension | 11 | 2.82% | 0.060 |  |  |  |  |
| Long-term potentiation | 5 | 1.28% | 0.078 |  |  |  |  |
| Ubiquitin mediated proteolysis |  |  |  |  | 6 | 2.56% | 0.060 |
| Epithelial cell signaling in *Helicobacter* *pylori* infection |  |  |  |  | 4 | 1.71% | 0.081 |
