# Supplementary material for: Multi-Platform Next-Generation Sequencing of the Domestic Turkey (Meleagris gallopavo): Genome Assembly and Analysis
Source: PLoS Biol. 2010 Sep 7;8(9):e1000475. doi: 10.1371/journal.pbio.1000475 (PMC2935454; doi:10.1371/journal.pbio.1000475)
Supplement: Table S9 — Wilcoxon rank sum test between innate immune related genes and the other genes. (0.03 MB DOC) [file pbio.1000475.s020.doc]

**Table S9.** Wilcoxon rank sum test between innate immune related genes and the other genes.

| **Species** | **WRS^a^** | ***P* value** | **Adjusted *P* value^b^** |
| --- | --- | --- | --- |
| **Mg_Gg** | 2692365 | 6.89E-01 | 6.89E-01 |
| **Mg_Tg** | 2596200 | 2.04E-02 | 2.30E-02 |
| **Gg_Tg** | 2538682 | 5.25E-04 | 1.77E-03 |
| **Hs_Mm** | 2544205 | 7.87E-04 | 1.77E-03 |
| **Hs_Cf** | 2562329 | 2.74E-03 | 4.11E-03 |
| **Hs_Md** | 2543636 | 7.55E-04 | 1.77E-03 |
| **Mm_Cf** | 2574794 | 6.02E-03 | 7.74E-03 |
| **Mm_Md** | 2532023 | 3.18E-04 | 1.77E-03 |
| **Cf_Md** | 2555492 | 1.74E-03 | 3.13E-03 |

^a^ The Wilcoxon rank sum test statistic

^b^ Benjamini and Hochberg FDR correction
