# Supplementary material for: Multi-Platform Next-Generation Sequencing of the Domestic Turkey (Meleagris gallopavo): Genome Assembly and Analysis
Source: PLoS Biol. 2010 Sep 7;8(9):e1000475. doi: 10.1371/journal.pbio.1000475 (PMC2935454; doi:10.1371/journal.pbio.1000475)
Supplement: Table S10 — Summary of homology-based RNA annotations from the sequenced genomes of turkey ( M. gallopavo ), chicken ( G. gallus ), and zebra finch ( T. gutatta ). Where a range of numbers is given, it remains uncertain whether multiple copies in the genomic DNA are true copies of the gene or assembly artifacts. (0.06 MB DOC) [file pbio.1000475.s021.doc]

**Table S10.** Summary of homology-based RNA annotations from the sequenced genomes of turkey (*M*. *gallopavo*), chicken (*G*. *gallus*), and zebra finch (*T*. *gutatta*). Where a range of numbers is given, it remains uncertain whether multiple copies in the genomic DNA are true copies of the gene or assembly artifacts.

| **RNA class** | **Functional Category** | ***Turkey*** | ***Chicken*** | ***Zebra finch*** |
| --- | --- | --- | --- | --- |
| 7SK | Transcription regulation | 1 | 1 | 1 |
| Hammerhead ribozymes | Self-cleaving |  |  |  |
| miRNA | Translation control | 432 | 475 | 280 |
| potassium channel motif | RNA editing |  |  |  |
| RNase MRP | Mitochondrial replication, rRNA processing | 1 (3’part) | 1 | 1 |
| RNase P | tRNA processing | 0 | 1 | 1 |
| 5S rRNA | Polypeptide synthesis | 4 | 5 | 42 |
| SnoRNA U3  other snoRNAs | Nucleolar rRNA processing  other snoRNAs processing | 1  194 | 1  200 | 1  177 |
| SRP | Protein transportation | 3 | 7 | 0 |
| Telomerase | Telomerase | 0 | 1 | 0 |
| tRNA | Polypeptide synthesis | 156 | 254 | 364 |
| U1  U2  U4  U5  U6  U11  U12  U4atac  U6atac | Splicing  Splicing  Splicing  Splicing  Splicing  Splicing  Splicing  Splicing  Splicing | 3  2  2  1  2  1  1  1  1 | 1  1  1  2  4  1  1  1  1 | 2  5  2  3  2  1  1  0  1 |
| U7 | Histone maturation | 2 | 1 | 1 |
| vault RNA | Drug resistance | 1 | 1 |  |
| Y-RNA | DNA replication | 3 | 3 |  |
| SECIS | Selenocystein insertion | 0-2 | 2 (15) |  |
| Histone3 | mRNA transport | 0-26 | 25 (40) |  |
| IRE | Iron metabolism | 0-5 | 6(9) |  |
| Vimentin3 | mRNA localization | 1 | 1(4) |  |
| CAESAR | Gene expression regulation | 1 | 1(4) |  |
| Antizyme FSE | Frame shifting promotion | 2 | 3 |  |
| IRES_Cx43  IRES_APC | Cap independent translation  Apoptotic cascade | 0-1  0-1 | 1(2)  1(2) |  |
| HAR1F | Unknown | 1 | 1(1) |  |
| NRON | Immune response | 1 | 2(1) |  |
