# Supplementary material for: Multi-Platform Next-Generation Sequencing of the Domestic Turkey (Meleagris gallopavo): Genome Assembly and Analysis
Source: PLoS Biol. 2010 Sep 7;8(9):e1000475. doi: 10.1371/journal.pbio.1000475 (PMC2935454; doi:10.1371/journal.pbio.1000475)
Supplement: Table S11 — Significantly enriched GO terms ( p <0.0001) in the set of putative targets for the chicken specific miRNAs. (0.04 MB DOC) [file pbio.1000475.s022.doc]

**Table S11.** Significantly enriched GO terms (*P* < 0.0001) in the set of putative targets for the chicken specific miRNAs.

| **miRNA** | **GO (ID)** | **GO Description** | ***P*−value** |
| --- | --- | --- | --- |
| miR-1456 | GO:0006826 | iron ion transport | 3.7e-05 |
| miR-1456 | GO:0006879 | cellular iron ion homeostasis | 3.7e-05 |
| miR-1456 | GO:0055072 | iron ion homeostasis | 3.7e-05 |
| miR-1456 | GO:0006873 | cellular ion homeostasis | 6.1e-05 |
| miR-1456 | GO:0030003 | cellular cation homeostasis | 6.1e-05 |
| miR-1456 | GO:0030005 | cellular di-, tri-valent inorganic cation homeostasis | 6.1e-05 |
| miR-1456 | GO:0055066 | di-, tri-valent inorganic cation homeostasis | 6.1e-05 |
| miR-1456 | GO:0055082 | cellular chemical homeostasis | 6.1e-05 |
| miR-1566 | GO:0015674 | di-, tri-valent inorganic cation transport | 4.0e-06 |
| miR-1566 | GO:0006826 | iron ion transport | 1.0e-05 |
| miR-1815 | GO:0006302 | double-strand break repair | 4.0e-07 |
| miR-466 | GO:0046700 | heterocycle catabolic process | 6.1e-07 |
| miR-466 | GO:0009063 | cellular amino acid catabolic process | 5.7e-06 |
| miR-466 | GO:0009310 | amine catabolic process | 5.7e-06 |
| miR-466 | GO:0044270 | nitrogen compound catabolic process | 6.7e-06 |
| miR-466 | GO:0016054 | organic acid catabolic process | 7.3e-06 |
| miR-466 | GO:0046395 | carboxylic acid catabolic process | 7.3e-06 |
