# Supplementary material for: Multi-Platform Next-Generation Sequencing of the Domestic Turkey (Meleagris gallopavo): Genome Assembly and Analysis
Source: PLoS Biol. 2010 Sep 7;8(9):e1000475. doi: 10.1371/journal.pbio.1000475 (PMC2935454; doi:10.1371/journal.pbio.1000475)
Supplement: Table S14 — Summary of gene orthologs defined from sequence homology, gene trees, and conservation of synteny for Ensembl gene predictions. (0.03 MB DOC) [file pbio.1000475.s025.doc]

**Table S14.** Summary of gene orthologs defined from sequence homology, gene trees and conservation of synteny for Ensembl gene predictions.

| **Species Comparison** | **No. Homologs** | **1:1 Orthologs** | **1:M Orthologs** | **M:M Orthologs** |
| --- | --- | --- | --- | --- |
| Turkey/Chicken | 14,155 | 12,607 | 849 | 65 |
| Chicken/Human | 16,782 | 11,429 | 1,141 | 119 |
