# Supplementary material for: Multi-Platform Next-Generation Sequencing of the Domestic Turkey (Meleagris gallopavo): Genome Assembly and Analysis
Source: PLoS Biol. 2010 Sep 7;8(9):e1000475. doi: 10.1371/journal.pbio.1000475 (PMC2935454; doi:10.1371/journal.pbio.1000475)
Supplement: Text S1 — Turkey GBrowse and GenBank submission. (0.03 MB DOC) [file pbio.1000475.s026.doc]

**SUPPORTING INFORMATION**

**Turkey GBrowse**

Birdbase Turkey GBrowse is an internet accessible browser designed to view the turkey genomic sequence and associated annotation (http://birdbase.net/cgi-bin/gbrowse/turkeygenome/). This resource is similar to Gallus GBrowse [1] and uses the Generic Model Organism Database GBrowse module [2] and a MySQL database to store and retrieve information. The current Turkey GBrowse provides a graphical interface grouped by themes including the genomic map, genes, transcripts, markers and synteny and a BLAT server that can be used to search the turkey genome with either nucleotide or protein sequences (http://birdbase.net/cgi-bin/turkeyblat). The genomic map includes the current DNA sequence grouped by chromosome along with the concatenated unlinked sequence designated as ChrUn. This section also includes a mapping of the scaffolds and contigs used to build the complete map. Genes are grouped by functions including those encoding: proteins, microRNAs, ribosomal RNAs, small nuclear and nucleolar RNAs, pseudogenes, retrotransposons, and miscellaneous RNAs. Currently, syntenic relationships between the turkey and chicken can be displayed and additional relationships will be added as they are mapped.

**GenBank Submission**

The generated chromosome AGP files and contiguous sequences from the turkey genome sequence project version 2.01 were processed using NCBI software Sequin and tbl2asn available at http://www.ncbi.nlm.nih.gov/Genbank/wgs.html. This Whole Genome Shotgun project is deposited at DDBJ/EMBL/GenBank under the accession ADDD00000000. The version described in this paper is the first version, ADDD01000000.

1. Schmidt CJ, Romanov M, Ryder O, Magrini V, Hickenbotham M, et al. (2008) Gallus GBrowse: a unified genomic database for the chicken. Nucleic Acids Res 36: D719-D723.

2. Stein LD, Mungall C, Shu S, Caudy M, Mangone M, et al. (2002) The generic genome browser: a building block for a model organism system database. Genome Res 12: 1599-1610.
